# The Immunologic Effect of Early Intravenous Two and Four Gram Bolus Dosing of Tranexamic Acid Compared to Placebo in Patients With Severe Traumatic Bleeding (TAMPITI): A Randomized, Double-Blind, Placebo-Controlled, Single-Center Trial

**DOI:** 10.3389/fimmu.2020.02085

**Published:** 2020-09-08

**Authors:** Philip C. Spinella, Kimberly A. Thomas, Isaiah R. Turnbull, Anja Fuchs, Kelly Bochicchio, Douglas Schuerer, Stacey Reese, Adrian A. Coleoglou Centeno, Christopher B. Horn, Jack Baty, Susan M. Shea, M. Adam Meledeo, Anthony E. Pusateri, Jerrold H. Levy, Andrew P. Cap, Grant V. Bochicchio, for the TAMPITI Investigators, M.D. Enyo Ablordeppey

**Affiliations:** ^1^Division of Pediatric Critical Care Medicine, Department of Pediatrics, Washington University School of Medicine, St. Louis, MO, United States; ^2^Section of Acute and Critical Care Surgery, Department of Surgery, Washington University School of Medicine, St. Louis, MO, United States; ^3^Division of Biostatistics, Washington University School of Medicine, St. Louis, MO, United States; ^4^United States Army Institute of Surgical Research, Joint Base San Antonio-Fort Sam Houston, San Antonio, TX, United States; ^5^Department of Anesthesiology and Critical Care, Duke University School of Medicine, Durham, NC, United States

**Keywords:** tranexamic acid, monocyte activation, immunology, hemostasis, trauma

## Abstract

**Background:**

The hemostatic properties of tranexamic acid (TXA) are well described, but the immunological effects of TXA administration after traumatic injury have not been thoroughly examined. We hypothesized TXA would reduce monocyte activation in bleeding trauma patients with severe injury.

**Methods:**

This was a single center, double-blinded, randomized controlled trial (RCT) comparing placebo to a 2 g or 4 g intravenous TXA bolus dose in trauma patients with severe injury. Fifty patients were randomized into each study group. The primary outcome was a reduction in monocyte activation as measured by human leukocyte antigen-DR isotype (HLA-DR) expression on monocytes 72 h after TXA administration. Secondary outcomes included kinetic assessment of immune and hemostatic phenotypes within the 72 h window post-TXA administration.

**Results:**

The trial occurred between March 2016 and September 2017, when data collection ended. 149 patients were analyzed (placebo, *n* = 50; 2 g TXA, *n* = 49; 4 g TXA, *n* = 50). The fold change in HLA-DR expression on monocytes [reported as median (Q1–Q3)] from pre-TXA to 72 h post-TXA was similar between placebo [0.61 (0.51–0.82)], 2 g TXA [0.57 (0.47–0.75)], and 4 g TXA [0.57 (0.44–0.89)] study groups (*p* = 0.82). Neutrophil CD62L expression was reduced in the 4 g TXA group [fold change: 0.73 (0.63–0.97)] compared to the placebo group [0.97 (0.78–1.10)] at 24 h post-TXA (*p* = 0.034). The fold decrease in plasma IL-6 was significantly less in the 4 g TXA group [1.36 (0.87–2.42)] compared to the placebo group [0.46 (0.19–1.69)] at 72 h post-TXA (*p* = 0.028). There were no differences in frequencies of myeloid or lymphoid populations or in classical complement activation at any of the study time points.

**Conclusion:**

In trauma patients with severe injury, 4 g intravenous bolus dosing of TXA has minimal immunomodulatory effects with respect to leukocyte phenotypes and circulating cytokine levels.

**Clinical Trial Registration:**

www.ClinicalTrials.gov, identifier NCT02535949.

## Introduction

In the United States (US), trauma is the leading cause of death in the population from 1 to 46 years of age (1), and each year in the United States there are an estimated 30,000 preventable deaths after traumatic injury due to hemorrhage (2). Trauma-induced coagulopathy (TIC) occurs in approximately 25% of injured patients (3–5). One of the primary mechanisms of TIC is hyperfibrinolysis (6). Observational data in multiple studies indicate that hyperfibrinolysis following traumatic injury is independently associated with an increased risk of mortality (7–10). Tranexamic acid (TXA), an antifibrinolytic agent, has become a standard of care in severely bleeding trauma patients (11–13). This practice is primarily based on the results of the landmark Clinical Randomization of an Antifibrinolytic in Significant Hemorrhage 2 (CRASH-2) trial, which demonstrated TXA administration within 3 h of injury increased survival in patients with significant hemorrhage (14), and on results from the Clinical Randomization of an Antifibrinolytic in Significant Hemorrhage 3 (CRASH-3) trial showing improved survival for patients with mild to moderate brain injury (15). However, certain aspects of the CRASH-2 results suggest that the effect of TXA in trauma patients may not be purely due to the inhibition of fibrinolysis. In particular, the data indicating increased mortality due to bleeding when TXA was given after 3 h (16) suggest that TXA may have additional biologic properties in this population.

Tranexamic acid is a lysine analog which irreversibly binds plasminogen thereby inhibiting fibrin binding, thus decreasing fibrinolysis and increasing clot stability (17, 18). Through this mechanism, TXA reduces bleeding. However, plasmin is an enzyme with a broad range of substrates affecting both hemostatic and immune function (19, 20). Plasmin promotes monocyte (21, 22), macrophage (23), and dendritic cell (24) migration through proteolytic mechanisms and through induction of actin polymerization. Plasmin has also been shown to potentiate phagocytosis in dendritic cells (25) and macrophages (26). Moreover, plasmin is a potent activator of monocytes via NF-κB (27), JAK/STAT (28), and 5-lipoxygenase signaling (29, 30). These pathways are associated with the production of proinflammatory cytokines (such as TNFα and IL-1β) (27), MCP-1 release (28), and leukotriene synthesis (31), respectively. Lastly, plasmin is a potent activator of complement [complement component 3 (C3) and complement component 5 (C5) cleavage (32, 33)], and complement itself is immune activating (34). To this end, the use of anti-fibrinolytics which reduce plasmin function may also result in a dampening of the inflammation and immune functions associated with the pathophysiology of traumatic injury.

Interestingly, *in vivo* studies in small rodents have demonstrated TXA is anti-inflammatory in polytrauma (35), TBI (36), and ischemia reperfusion injury (37) models. In humans, the inflammatory nature of TXA is less well defined. In one study of orthopedic surgery patients, intravenous TXA administration both before and after surgery led to increased levels of circulating proinflammatory molecules, such as TNF-α, IL-1β, and IL-6 (38). Conversely, in another cohort of orthopedic surgery patients followed up to 48 h, it was found that an increased number of post-operative oral doses of TXA suppressed IL-6 levels (39). Moreover, prophylactic TXA use in cardiac surgery patients was shown to have an “immune-enhancing” effect, as leukocytes from patients receiving TXA had decreased expression of immunosuppressive surface markers (LAP and PD-L1), and increased expression of immune-activating markers (CD83 and CCR7) (40). This study also showed that IL-1β, but not other proinflammatory cytokines, was significantly increased in the TXA group compared to the placebo group. These contrasting immune phenotypes of TXA in rodent trauma models compared to humans with surgical bleeding highlights the need for further evaluation of the immunomodulatory effects of TXA in trauma patients.

Based on the role of plasmin as an immune activator, and the inhibition of plasmin by TXA, we hypothesized that TXA would (i) reduce monocyte and complement activation and (ii) decrease fibrinolysis in a dose-dependent manner in traumatically injured patients. To test these hypotheses, we performed a double-blinded randomized controlled trial (RCT) comparing placebo to 2 g or 4 g intravenous (i.v.) bolus TXA doses to examine the immunological consequences of TXA administration in severely injured patients. Given the pleiotropic effects of plasmin on immune and hemostatic function, we also performed extensive exploratory immune phenotyping on enrolled subjects.

## Materials and Methods

### Study Design and Treatment Protocols

This study was a prospective single-center double-blinded RCT, and was performed at Wash U, a tertiary care center. The trial was approved by the US FDA (IND 125420), and by the Institutional Review Board at Wash U, and the United States Army Medical Research and Materiel Command Human Research Protections Office. The trial was performed with exception from informed consent including community consultation with delayed patient or legally authorized representative consent. In addition, the trial was overseen by an independent research monitor and an external data and safety monitoring board. This study was conducted in accordance with Good Clinical Practice guidelines, the Declaration of Helsinki, and Title 21 of the Code of Federal Regulations. The full clinical trial protocol can be found in the Supplementary Material. The trial is registered at ClinicalTrials.gov (NCT02535949) and the trial is closed for accrual.

### Patients

Trained research staff consecutively screened all patients requiring a Level I trauma activation at a single American College of Surgeons-verified Level I academic trauma center for potential inclusion in the study. Patients were eligible for inclusion if they were 18 years of age or older, sustained a traumatic injury which required them to receive at least one unit of red blood cells (RBC) or required an emergent operation for possible bleeding control, and were able to receive the study medication (TXA or placebo) within 2 h of time of injury. Patients were excluded if they had a known past medical history of thrombotic events, seizures, or subarachnoid hemorrhage, or met other exclusion criteria (the full list of exclusion criteria can be found in Supplementary Table S1).

### Randomization and Masking

For patient randomization, SAS programs were written to generate the randomization order and the programs were implemented online using REDCap. Randomization was blocked using random block sizes. Concealment was accomplished by virtue of the online procedures that prevented group assignment from being known until the actual subject was ready to be randomized. There was no stratification. With the exception of the investigational drug service (IDS) staff preparing study drug, all participants, study team members, and investigators were blinded to the treatment arms. The IDS maintained the blind by placing study drug or placebo in a syringe that was labeled only with the TXA study number, and the study number was linked to the randomization group maintained only by IDS. The volume and color of the fluid in the syringe were identical for each study drug or placebo. Coordinators verified the TXA study number used for each subject in a log prior to dosing.

### Procedures and Clinical Data Collection

After patient enrollment, demographics, injury characteristics, baseline physiological parameters, surgical procedures, concomitant medications, and blood product(s) administration were collected. Eligible patients were randomized via fixed-allocation computer randomization to receive either placebo, 2 gram (g) of TXA, or 4 g of TXA in 40 milliliters (mL) of normal saline i.v. over 10 min. The doses of 2 g and 4 g i.v. were based on the TXA bolus dosing ranges from previous trials examining TXA that range from 15 to 100 milligram (mg)/kilogram (kg) i.v. bolus (40). A 2 and 4 g dose of TXA would be 25 mg/kg and 50 mg/kg, respectively, in an 80 kg patient. A 100 mg/kg dose of TXA in a 80 kg patient would be equal to an 8 g i.v. dose. A single bolus dose was implemented to accommodate a dosing regimen that would be simpler to implement in United States military deployed settings that are often in austere environments. Soft tissue oxygen tension was measured with each blood draw using InSpectra StO_2_ Spot Check device (Hutchinson Technology, Hutchinson, MN, United States). A baseline blood draw was performed prior to administration of study drug (0 h, T0) and at pre-specified intervals after study drug administration: 6 h (T6), 24 h (T24), and 72 h (T72). Patients were followed until discharge or study day 28, whichever came first. Study participants were assessed daily for length of stay (LOS) in the Intensive Care Unit (ICU), ventilator days, mortality, thromboembolic events, abnormal clinical laboratory values, and safety and adverse events.

### Outcomes

The primary trial outcome was a reduction in monocyte activation, measured by Human Leukocyte Antigen-DR isotype (HLA-DR) expression on monocytes, from zero to 72 h after TXA administration. Reduction in HLA-DR expression was chosen as the primary outcome as it has been associated with clinical outcomes, such as the risk of sepsis, in patients with traumatic injury (41). Secondary outcomes included other measures of immune function, hemostasis, and endothelial function. Secondary clinical outcomes included multiple organ dysfunction syndrome (MODS), thromboembolic events, and mortality at 28 days. MODS scores were assessed daily by calculation of the Marshall Score (42).

### Immunophenotyping

For immunophenotyping, blood was collected in K_2_EDTA Vacutainer Blood Collection Tubes. Blood was fixed within 2 h of sample collection by mixing 1.25 mL whole blood (WB) with 1.75 mL Proteomic Stabilizer (Smart Tube Inc., San Carlos, CA, United States) and incubating for 10 min at room temperature (RT). Subsequently, samples were frozen at −80°C. Flow cytometric immunophenotyping was performed in batch analysis, processing samples from all time points of two to four patients simultaneously. Samples were thawed, subjected to RBC lysis and washing steps per manufacturer’s recommendations. Cells were incubated with Fc receptor blocking reagent (TruStain FcX, Biolegend, San Diego, CA, United States) and stained with fluorochrome-conjugated antibodies (Panels 1, 2, and 3; see Supplementary Table S2). Samples were acquired on an LSR Fortessa flow cytometer using Diva software (both from BD Biosciences, San Jose, CA, United States) and analyzed with the FlowJo v10.2 software (Treestar, Ashland, OR, United States). Analysis of the individual peripheral blood leukocyte subsets was done as specified by gating strategies in Supplementary Table S3. Cell concentrations in stained samples were determined by adding counting beads to the samples immediately before flow cytometric acquisition (123count eBeads, eBioscience, San Diego, CA, United States). Cell concentrations in samples were calculated per manufacturer’s directions and were used to determine the cell counts per mL of WB.

### Soluble Inflammatory Mediator Levels and Complement Activation Assessment

Soluble inflammatory mediators (cytokines and chemokines) were analyzed in the Immunomonitoring Laboratory (Wash U) using the 384-well Human High Sensitivity T cell Milliplex assay, which detects 21 cytokines and chemokines (HSTCMAG384-PX21; EMD Millipore, Billerica, MA, United States). Platelet-poor plasma (PPP) samples (isolation protocol in Supplementary Methods) were thawed on ice, mixed, then centrifuged at 10,000*g* for 5 min at 4°C to remove particulates. PPP was then diluted 1:4 in assay buffer and tested in duplicate as per the manufacturer’s recommendations. A FLEXMAP3D Luminex analyzer was used to determine median fluorescence intensity (MFI) for 50 beads per analyte. MFIs were compared to the standard curves that generated pg/mL values for each analyte using a 5-parameter logistic regression analysis program (Milliplex Analyst 5.1.0, Vigene Tech Inc., Carlisle, MA, United States). For total hemolytic complement activation measurement (CH50, Iu/mL), PPP was thawed at RT and assessed using a CH50 assay (#A018, Quidel, San Diego, CA, United States) per manufacturer’s suggested protocol.

### Hemostatic and Endothelial Assessment

Stored PPP samples from each patient were assessed for hemostatic function. Specifically, clotting in response to kaolin activation via thromboelastography (TEG) was performed with PPP (43–49) according to manufacturer’s protocol (#07-004, Haemonetics, Braintree, MA, United States) using a TEG5000. R time (min), K time (min), alpha angle (°), and maximum amplitude (millimeters, mm) were reported. Both mechanical and immunoturbidimetric methods via STA-Compact Max or STA-R were used to measure PPP levels of fibrinogen (FIB, mg/deciliter (dL); STA-FIBRINOGEN 5, #00674, Diagnostica Stago, Parsippany, NJ, United States), von Willebrand factor (VWF,%; STA LIATEST VWF:AG, #00518, Diagnostica Stago), and D-dimer (micrograms (μg)/mL; STA LIATEST D DI, #00515, Diagnostica Stago). Manufacturer’s protocols were followed for FIB, VWF, and D-dimer. Enzyme-linked immunosorbent assays (ELISAs) were used for the following measurements, following the manufacturer’s suggested protocol for each assay: thrombin-antithrombin complex (TAT, #OWMG15, Siemens, Munich, Germany), tissue-type plasminogen activator (tPA, #BMS258-2, Thermo Fisher Scientific, Carlsbad, CA, United States), prothrombin fragment 1 + 2 (PF1.2, #027756, US Biological Life Sciences, Salem, MA, United States), kininogen (KNG1, #KA1040, Novus Biologicals, Littleton, CO, United States), plasmin/alpha-2-antiplasmin (PAP, #603, BioMedica Diagnostics, Windsor, Nova Scotia, Canada), syndecan-1 (SDC1, #EHSDC1, Thermo Fisher Scientific).

### Statistics and Power Calculations

This study was powered to assess the effect of TXA on HLA-DR expression on monocytes. A 10% decrease in the frequency of CD14^+^/HLA-DR^+^ monocytes was considered by the investigators to be potentially clinically relevant. Using a two-tailed alpha of 0.05, a power of 0.90, and accounting for a 20% drop-out rate, we estimated 47 patients per arm. To account for missing data, we aimed to enroll 50 patients per study group for a total of 150 patients. No interim analysis was planned, and the study was stopped upon reaching the planned sample size in all three study groups. Baseline demographics, and injury, physiological, and outcome variables were described and compared for participants in each study group. Percentages were used to describe categorical variables, and Pearson chi-square or Fisher’s Exact tests were used for tests for differences among groups. Continuous and ordinal variables were described by medians and interquartile ranges and differences tested with Kruskal–Wallis tests. Systolic blood pressure, Glasgow Coma Score (GCS), abbreviated injury score (AIS) and injury severity score (ISS), were dichotomized and analyzed with chi-squared tests. For immune and hemostatic phenotyping laboratory-based tests, results at a given time point (T0, T6, T24, and T72) between treatment groups (placebo, 2 g TXA, 4 g TXA) were compared by non-parametric analysis of variance (ANOVA; Kruskal–Wallis test), and if found significant, further analyzed using a Dunn’s multiple comparison test to determine which groups were significantly different from the placebo group. For all kinetic analyses, only patients with full sets of data [samples at each of the four time points (T0, T6, T24, and T72)] were analyzed in order to accurately assess changes over time. To do these analyses, a fold change methodology was employed, whereby values acquired at T6, T24, and T72 were normalized to baseline (T0) values for all patients with samples available at all four time points (n of patients for each graph and paired analysis are delineated in figure legends). Specifically, fold change was calculated as [(frequency/MFI/concentration of a given population/analyte at the given time | | T6,T24,T72| |) ÷ (frequency/MFI/concentration of the same population/analyte at T0)]. For analyses pertaining to data from all patients with available samples, results at a given time point (T0, T6, T24, and T72) between treatment groups (placebo, 2 g TXA, 4 g TXA) were compared by non-parametric analysis of variance (ANOVA; Kruskal–Wallis test) and are located in Supplementary Tables S4–S6. SAS (v9.4 for Windows, SAS Institute Inc., Cary, NC, United States) and Prism 8 (for Windows 64-bit (v8.1.0), GraphPad Software, San Diego, CA, United States) were used for statistical analyses and correlation matrices calculations and visual representations. Principal component analysis (PCA) was performed using R version 3.6.2 (R Core Team, 2019, R Foundation for Statistical Computing, Vienna, Austria), *stats* (used prcomp() function; 3.6.2, R Core Team, 2019), and *ggplot2* (3.3.0, Wickham H, 2016) packages. Code used can be found in Supplementary Methods.

## Results

**FIGURE 1:**
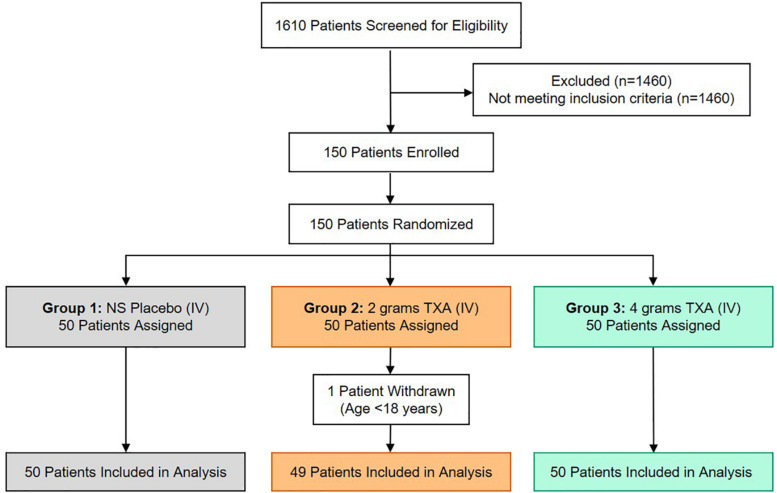
TAMPITI Patient Screening, Enrollment, Randomization, and Analysis Diagram. This Consolidated Standards of Reporting Trials (CONSORT) diagram displays the number of patients screened for eligibility, excluded based on not meeting study inclusion criteria, patients enrolled and randomized, and the patients analyzed per study group.

There were 150 patients enrolled in the trial between March 2016 and September 2017. One patient was withdrawn from the 2 g TXA group when it was determined that they were less than 18 years of age and in violation of eligibility criteria. As a result, 149 patients were included in the analysis, with 50 patients in the placebo group, 49 patients in the 2 g TXA group, and 50 patients in the 4 g TXA group (Figure 1 and Supplementary Table S1). There were no differences in baseline patient demographics or in measures of severity of injury between the three study groups (Table 1). The time in minutes from injury to administration of TXA was similar between placebo, 2 g TXA, and 4 g TXA study groups [median (Q1–Q3): 95.5 min (81.0–103) vs. 97.0 min (85.0–111) vs. 96.5 min (77.0–106), respectively; *p* = 0.47]. The median time in minutes from injury to T0 immune assessment was 57.5 (46.8–74.3) for placebo, 67.0 (46.5–85.0) for 2 g TXA, and 60.0 (50.0–82.8) for the 4 g TXA treatment group (Table 1). The amount of blood products given before TXA administration was also similar between the three study groups (Table 1). There were no differences in clinical outcomes, to include mortality, or the amount of blood products given after TXA administration between the three study groups (Table 2). However, there was a difference in the incidence of thromboembolic events (TE) between study groups that approached significance [placebo, 12.0%; 2 g (26.5%); 4 g (32.0%)], (*p* = 0.05; Table 2).

**TABLE 1:** Patient demographics, baseline characteristics, and clinical outcomes in each study group. Values are reported as median (Q1–Q3) unless otherwise indicated.

Variable	n	Placebo	n	2 g TXA	n	4 g TXA	p-value
**Demographics**							
Age (years)	50	27.0(22.0−34.0)	49	26.0(22.0−40.0)	50	31.0(25.0−44.0)	0.13
Male [n, (%)]	50	45(90.0%)	49	44(90.0%)	50	42(84.0%)	0.58
BMI	47	26.5(22.8−30.0)	46	25.4(23.1−29.5)	50	25.2(23.7−29.9)	0.34
Hispanic [n, (%)]	50	1(2.00%)	49	2(4.08%)	50	1(2.00%)	0.76
African American [n, (%)]	49	44(88.0%)	49	42(85.7%)	49	43(86.0%)	0.94
Injury to admission time (min)	50	30.0(22.0−39.0)	49	28.0(20.0−35.0)	50	28.0(21.0−38.0)	0.44
Injury to T0 blood sample (min)	50	57.5(47.0−74.0)	49	67.0(48.0−85.0)	50	60.0(50.0−82.0)	0.46
Injury to TXA administration (min)	50	95.5(81.0−103)	49	97.0(85.0−111)	50	96.5(77.0−106)	0.48
**Injury severity**							
Penetrating Injury [n, (%)]	50	41(82.0%)	49	38(77.6%)	50	39(78.0%)	0.83
Heart Rate (BPM)	49	97.0(83.0−120)	49	102(90.0−128)	50	100.5(80.0−117.0)	0.39
SBP (mmHg)	48	120(98.0−141)	49	119(90.0−142)	50	118(98.0−135)	0.98
SBP ≤ 90 mmHg [n, (%)]	49	8(16.0%)	49	14(28.6%)	50	7(14.0%)	0.15
Temperature (°C)	26	36.5(35.8−36.7)	21	36.5(36.3−36.7)	27	36.5(36.3−36.7)	0.17
GCS	50	15.0(12.0−15.0)	49	15.0(11.0−15.0)	50	15.0(14.0−15.0)	0.26
GCS ≤ 8 [n, (%)]	50	10(20.0%)	49	12(24.5%)	50	5(10.0%)	0.16
NIRS (%)	26	87.5(80.0−90.0)	22	88.0(84.0−91.0)	20	87.5(78.0−93.0)	0.50
Creatinine (mg/dL)	33	1.30(1.13−1.61)	45	1.17(1.03−1.37)	48	1.22(0.99−1.39)	*0.03*
Platelet count (X10^6^/mL)	50	215(179−273)	48	229(166−287)	47	217(185−258)	0.78
Fibrinogen (mg/dL)	45	180(146−251)	39	190(143−222)	44	185(149−245)	0.41
INR	46	1.12(1.05−1.23)	43	1.12(1.06−1.26)	44	1.10(1.03−1.16)	0.18
Lactate (mmol/L)	44	4.70(3.50−8.80)	43	4.50(3.30−8.70)	40	4.45(2.70−7.15)	0.57
APACHE II	50	19.0(10.0−27.0)	49	16.0(11.0−24.0)	50	16.0(9.00−25.0)	0.74
ISS	46	19.5(14.0−34.0)	43	22.0(16.0−34.0)	44	19.0(14.0−29.0)	0.47
AIS Head ≥ 3 [n, (%)]	50	7(14.0%)	49	1(2.00%)	50	7(14.0%)	0.08
AIS Face ≥ 3 [n, (%)]	50	0(0%)	49	0(0%)	50	0(0%)	n/a
AIS Chest ≥ 3 [n, (%)]	50	22(44.0%)	49	27(55.1%)	50	20(40.0%)	0.30
AIS Abdomen ≥ 3 [n, (%)]	50	29(58.0%)	49	28(57.1%)	50	25(50.0%)	0.68
AIS Extremity ≥ 3 [n, (%)]	50	20(40.0%)	49	22(44.9%)	50	22(44.0%)	0.87
AIS External ≥ 3 [n, (%)]	50	1(2.00%)	49	1(2.04%)	50	4(8.00%)	0.22
**Pre-TXA blood product administration - Number transfused and volume received**							
RBC Recipients [n, (%)]	50	36(72.0%)	49	36(73.5%)	50	34(68.0%)	0.82
RBC Volume (mL/Kg)	47	8.73(0.00−31.2)	46	13.4(0.00−31.78)	50	9.50(0.00−36.5)	0.79
Plasma Recipients [n, (%)]	50	20(40.0%)	49	25(51.0%)	50	24(48.0%)	0.52
Plasma Volume (mL/Kg)	47	0.00(0.00−16.0)	46	0.00(0.00−15.9)	50	0.00(0.00−18.8)	0.84
Platelet Recipients [n, (%)]	50	17(34.0%)	49	18(36.7%)	50	13(26.0%)	0.49
Platelet Volume (mL/Kg)	47	0.00(0.00−2.53)	46	0.00(0.00−2.42)	47	0.00(0.00−2.00)	0.69
Cryo Recipients [n, (%)]	50	0(0%)	49	1(2.04%)	50	0(0%)	0.36
Cryo Volume (mL/Kg)	47	0.00(0.00−0.00)	46	0.00(0.00−0.00)	50	0.00(0.00−0.00)	0.35

**P-*values reported are for comparisons between all three study groups as described in the methods. BMI, body mass index; TXA, tranexamic acid; BPM, beats per minute; SBP, systolic blood pressure; mmHg, millimeters mercury; GCS, glasgow coma score; NIRS, near-infrared spectroscopy; INR, international normalized ratio APACHE II, acute physiology, age, chronic health evaluation II; ISS, injury severity score; AIS, abbreviated injury scale; Pre-TXA, prior to TXA administration; RBC, red blood cell; Cryo, cryoprecipitate.*

**TABLE 2:** Blood product usage and clinical outcomes in all three treatment groups.

Variable	n	Placebo	n	2 g TXA	n	4 g TXA	p-value
**Outcomes**							
ICU admission [n, (%)]	50	37(74.0%)	49	36(73.5%)	50	38(76.0%)	0.96
Mechanical ventilation [n, (%)]	50	30(60.0%)	48	28(58.3%)	49	30(61.2%)	0.96
ICU-free Days	50	27.3(17.4−28.6)	45	27.1(24.0−29.4)	49	27.1(24.3−29.0)	0.77
Max MODS in 7 days	49	4.00(1.00−7.00)	49	4.00(1.00−6.00)	50	4.00(1.00−8.00)	0.79
Seizure [n, (%)]	49	0(0.00%)	44	1(2.27%)	48	2(4.17%)	0.42
TE [n, (%)]	50	6(12.0%)	49	13(26.5%)	50	16(32.0%)	0.05
28-day mortality [n, (%)]	49	6(12.2%)	44	5(11.4%)	48	4(8.33%)	0.81
**Post-TXA blood product administration - Number transfused and volume received**							
RBC Recipients [n, (%)]	50	22(44.0%)	49	25(51.0%)	50	24(46.0%)	0.78
RBC Volume (mL/Kg)	47	0.00(0.00−13.2)	46	0.00(0.00−11.4)	50	0.00(0.00−19.3)	0.92
Plasma Recipients [n, (%)]	50	17(34.0%)	49	12(24.5%)	50	15(30.0%)	0.58
Plasma Volume (mL/Kg)	47	0.00(0.00−6.83)	46	0.00(0.00−0.00)	50	0.00(0.00−6.73)	0.99
Platelet Recipients [n, (%)]	50	15(30.0%)	49	13(26.5%)	50	13(26.0%)	0.89
Platelet Volume (mL/Kg)	47	0.00(0.00−2.96)	46	0.00(0.00−0.00)	50	0.00(0.00−2.42)	0.85
Cryo Recipients [n, (%)]	50	7(14.0%)	49	7(14.3%)	50	8(16.0%)	0.95
Cryo Volume (mL/Kg)	47	0.00(0.00−0.00)	46	0.00(0.00−0.00)	50	0.00(0.00−0.00)	0.43

*Values are reported as median (Q1-Q3) unless otherwise indicated. *P*-values reported are for comparisons between all three study groups as described in the section “Materials and Methods.” ICU, intensive care unit; RBC, red blood cell; Cryo, cryoprecipitate; Post-TXA, after TXA administration; MODS, multiple organ dysfunction score; TE, thromboembolic event.*

**FIGURE 2:**
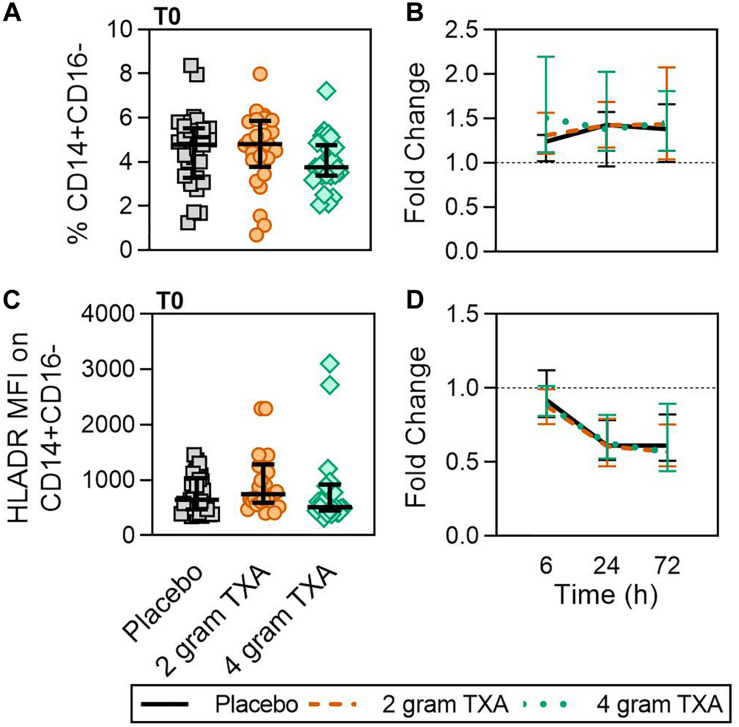
TXA administration does not alter monocyte expression of HLA-DR. For all kinetic data, fold change was calculated as [(frequency/MFI/concentration of a given population/analyte at the given time | | T6,T24,T72| |) ÷ (frequency/MFI/concentration of the same population/analyte at T0)]. Data are displayed as line graphs (fold change, median + IQR). The dashed line placed at *y* = 1 represents T0, or baseline. Placebo, *n* = 29; 2 g TXA, *n* = 24; 4 g TXA, *n* = 25. Statistical differences between the 4 g TXA and placebo groups at each given time point, as calculated via Kruskal-Wallis, are noted as follows: *p* > 0.05, not significant and not denoted on the graphical representation. **(A)** The frequency of CD14^+^CD16^–^ monocytes found in total peripheral blood leukocytes at T0 in all three treatment groups; data are represented as individual points with median + IQR for error bars. **(B)** Fold change in frequency of CD14^+^CD16^–^ monocytes found in total peripheral blood leukocytes over the study period of 72 h. **(C)** HLA-DR MFI on CD14^+^CD16^–^ monocytes in all three treatment groups at T0. **(D)** Fold change in HLA-DR MFI on CD14^+^CD16^–^ monocytes over the study period of 72 h.

At T0, there was no difference in the frequency of circulating CD14^+^CD16^–^ monocytes between the three treatment groups (Figure 2A). In addition, by 72 h post-TXA administration, the frequency of CD14^+^CD16^–^ monocytes in all three treatment groups had increased roughly 1.4-fold (median (Q1-Q3): placebo, 1.38 (1.01–1.66); 2 g TXA, 1.44 (1.04–2.08); 4 g TXA, 1.45 (1.14–1.81); Figure 2B). Despite this increase seen in frequency, HLA-DR expression on CD14^+^CD16^–^ monocytes was reduced in all treatment groups, suggesting TXA dose had no effect on monocyte HLA-DR expression at any time point (Figure 2C). The fold change of HLA-DR expression on monocytes from pre-TXA to 72 h post-TXA was similar between placebo [0.61 (0.51–0.82)], 2 g TXA [0.57 (0.47–0.75)], and 4 g TXA [0.57 (0.44–0.89)] study groups (*p* = 0.82, Figure 2D). These data demonstrate that neither 2 g nor 4 g TXA dosing differentially alters monocyte frequency or HLA-DR expression as compared to placebo treatment. In addition, these data show that traumatic injury leads to increased frequencies of circulating monocytes with decreased HLA-DR levels by 3 days post injury. In addition to HLA-DR, we also measured surface expression of CD86 and CD16. We found that CD86 expression on classical monocytes initially decreased, but changes in expression were similar across all three treatment groups (data not shown). These changes were not seen in non-classical monocytes, and there were little to no alterations in CD16 expression on both monocyte populations (data not shown).

**FIGURE 3:**
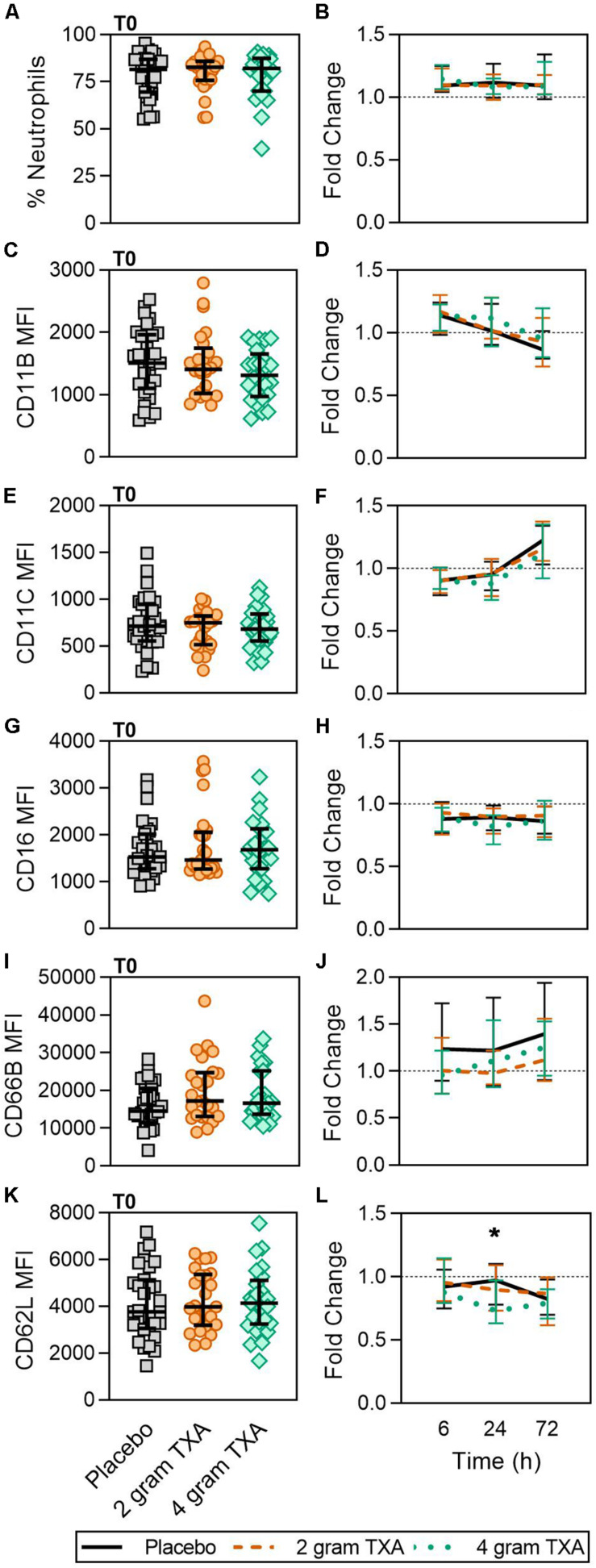
TXA administration does not alter neutrophil frequencies or phenotype. Placebo, *n* = 29; 2 g TXA, *n* = 24; 4 g TXA, *n* = 25. Statistical differences between the 4 g TXA and placebo groups at each given time point, as calculated via Kruskal-Wallis, are noted as follows: *p* < 0.05, *; *p* > 0.05, not significant and not denoted on the graphical representation. **(A)** The frequency of neutrophils found in total peripheral blood leukocytes at T0 in all three treatment groups; data are represented as individual points with median + IQR for error bars. **(B)** Fold change in neutrophil frequency over the study period of 72 h. **(C)** CD11B MFI on neutrophils in all three treatment groups at T0. **(D)** Fold change in CD11B MFI on neutrophils over the study period of 72 h. **(E)** CD11C MFI on neutrophils in all three treatment groups at T0. **(F)** Fold change in CD11C MFI on neutrophils over the study period of 72 h. **(G)** CD16 MFI on neutrophils in all three treatment groups at T0. **(H)** Fold change in CD16 MFI on neutrophils over the study period of 72 h. **(I)** CD66B MFI on neutrophils in all three treatment groups at T0. **(J)** Fold change in CD66B MFI on neutrophils over the study period of 72 h. **(K)** CD62L MFI on neutrophils in all three treatment groups at T0. **(L)** Fold change in CD62L MFI on neutrophils over the study period of 72 h.

As TXA inhibits the production of the pleiotropic immune activator plasmin, we performed extensive immune and hemostatic phenotyping in this study in order to identify if TXA administration would lead to either a global or a unique immune phenotype in traumatically injured patients. Looking at other circulating myeloid cell types, we found no difference in neutrophil frequencies at the time of or after TXA administration (Figures 3A,B). Surface phenotyping of neutrophils revealed little to no changes in surface expression of CD11B, CD11C, CD16, or CD66B (Figures 3C–J). However, while CD62L MFI was similar between all three groups at T0 (Figure 3K), there was a statistically significant reduction in CD62L expression in the 4 g TXA group [fold change: 0.73 (0.63–0.97)] compared to the placebo group [0.97 (0.78–1.10)] at 24 h post treatment (*p* = 0.034, Figure 3L). There were no profound differences in eosinophil, basophil, or conventional dendritic cell (cDC) populations either between treatment groups at T0, or between treatment groups over the course of the study period (data not shown, Supplementary Table S4).

**FIGURE 4:**
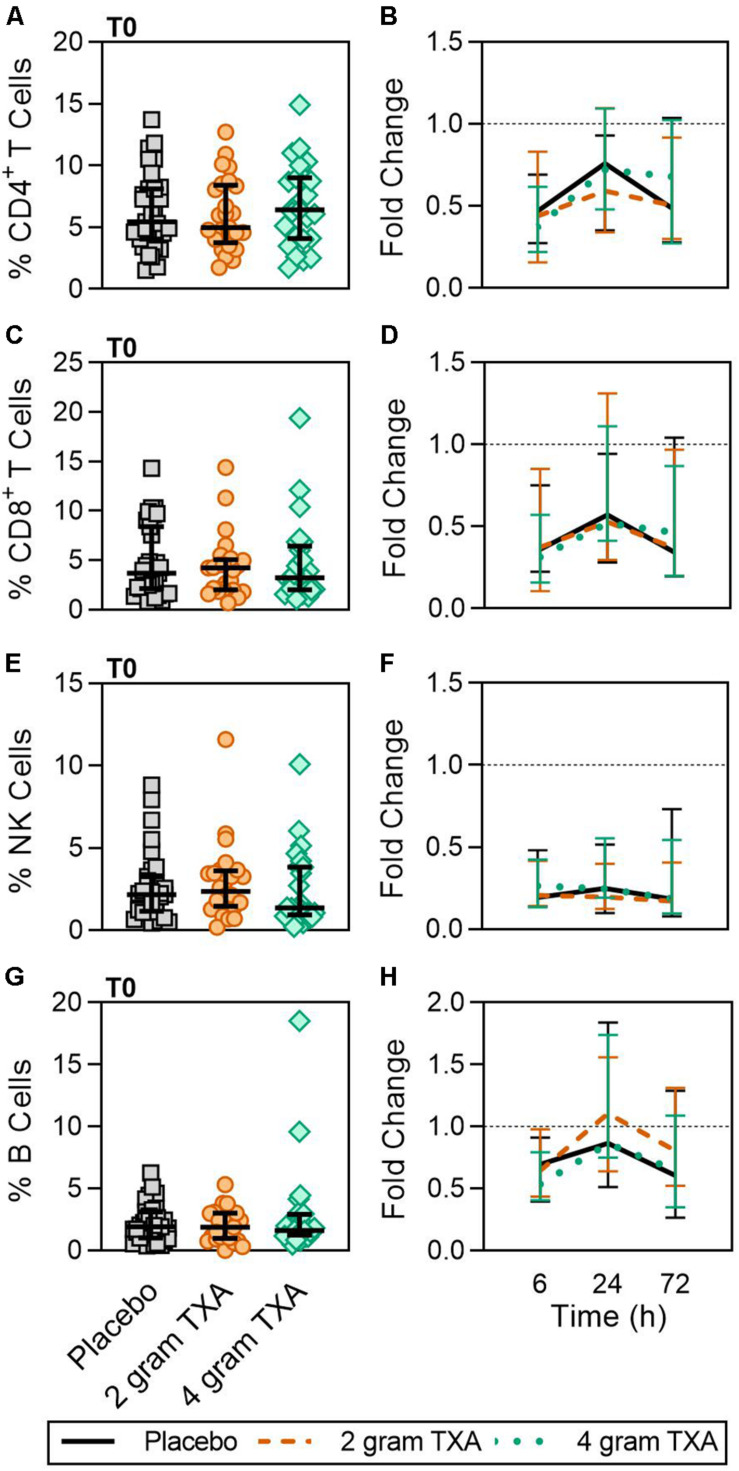
Traumatic injury, not TXA, decreases circulating lymphoid populations. For T0, data are represented as individual points with median + IQR for error bars. For all kinetic data, fold change was calculated as [(frequency/MFI/concentration of a given population/analyte at the given time | | T6,T24,T72| |) ÷ (frequency/MFI/concentration of the same population/analyte at T0)]. Data are displayed as line graphs (fold change, median + IQR). The dashed line placed at *y* = 1 represents T0, or baseline. Placebo, *n* = 29; 2 g TXA, *n* = 24; 4 g TXA, *n* = 25. Statistical differences between the 4 g TXA and placebo groups at each given time point, as calculated via Kruskal–Wallis, are noted as follows: *p* > 0.05, not significant and not denoted on the graphical representation. **(A)** The frequency of CD4 + T cells found in total peripheral blood leukocytes at T0 in all three treatment groups. **(B)** Fold change in frequency of CD4 + T cells found in total peripheral blood leukocytes over the study period of 72 h. **(C)** The frequency of CD8 + T cells found in total peripheral blood leukocytes at T0 in all three treatment groups. **(D)** Fold change in frequency of CD8 + T cells found in total peripheral blood leukocytes over the study period of 72 h. **(E)** The frequency of NK cells found in total peripheral blood leukocytes at T0 in all three treatment groups. **(F)** Fold change in frequency of NK cells found in total peripheral blood leukocytes over the study period of 72 h. **(G)** The frequency of B cells found in total peripheral blood leukocytes at T0 in all three treatment groups. **(H)** Fold change in frequency of B cells found in total peripheral blood leukocytes over the study period of 72 h.

**FIGURE 5:**
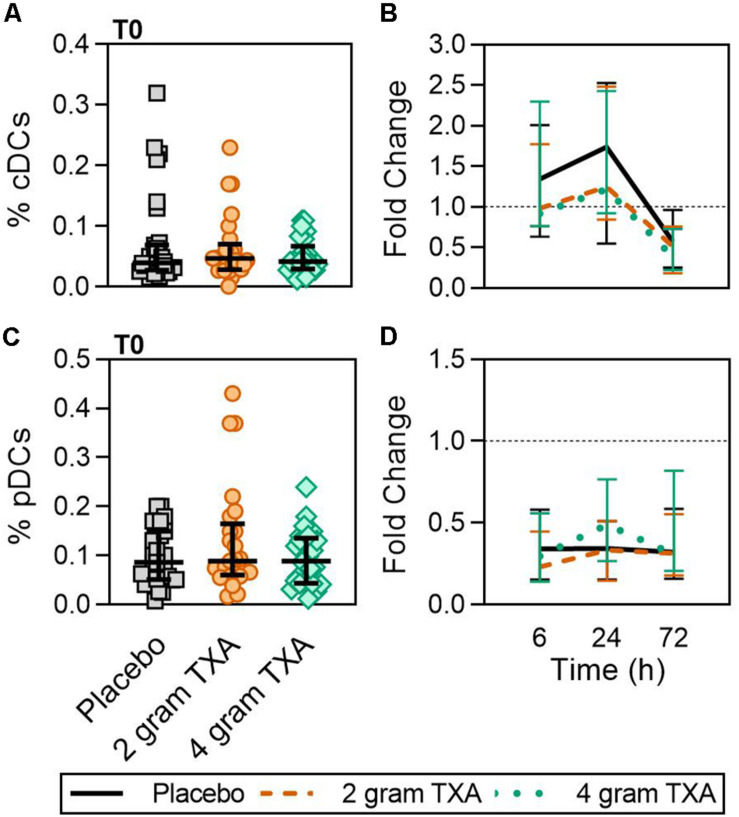
TXA administration does not alter conventional or plasmacytoid DC population changes in response to trauma. For T0, data are represented as individual points with median + IQR for error bars. For all kinetic data, fold change was calculated as [(frequency/MFI/concentration of a given population/analyte at the given time | | T6,T24,T72| |) ÷ (frequency/MFI/concentration of the same population/analyte at T0)]. Data are displayed as line graphs (fold change, median + IQR). The dashed line placed at *y* = 1 represents T0, or baseline. Placebo, *n* = 29; 2 g TXA, *n* = 24; 4 g TXA, *n* = 25. Statistical differences between the 4 g TXA and placebo groups at each given time point, as calculated via Kruskal-Wallis, are noted as follows: *p* > 0.05, not significant and not denoted on the graphical representation. **(A)** The frequency of conventional dendritic cells (cDCs) found in total peripheral blood leukocytes at T0 in all three treatment groups. **(B)** Fold change in frequency of cDCs found in total peripheral blood leukocytes over the study period of 72 h. **(C)** The frequency of plasmacytoid dendritic cells (pDCs) found in total peripheral blood leukocytes at T0 in all three treatment groups. **(D)** Fold change in frequency of pDCs found in total peripheral blood leukocytes over the study period of 72 h.

**FIGURE 6:**
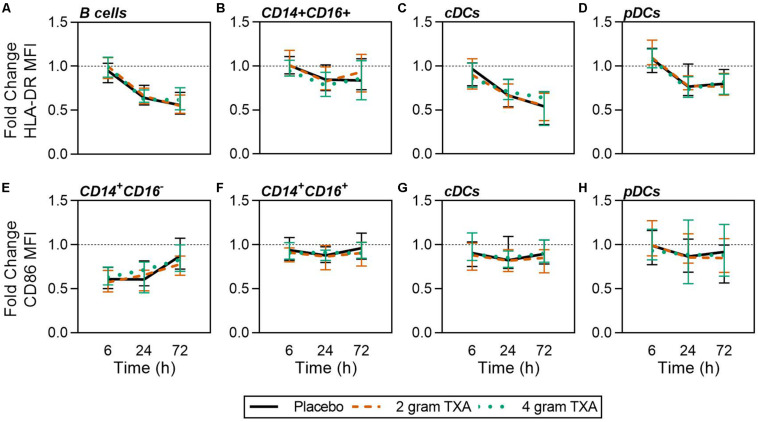
Global reduction in HLA-DR and not CD86 expression in response to traumatic injury. For all kinetic data, fold change was calculated as [(frequency/MFI/concentration of a given population/analyte at the given time | | T6,T24,T72| |) ÷ (frequency/MFI/concentration of the same population/analyte at T0)]. Data are displayed as line graphs (fold change, median + IQR). The dashed line placed at *y* = 1 represents T0, or baseline. Placebo, *n* = 29; 2 g TXA, *n* = 24; 4 g TXA, *n* = 25. Statistical differences between the 4 g TXA and placebo groups at each given time point, as calculated via Kruskal–Wallis, are noted as follows: *p* > 0.05, not significant and not denoted on the graphical representation. Fold change in HLA-DR MFI on **(A)** B cells, **(B)** CD14 + CD16 + non-classical monocytes, **(C)** cDCs, and **(D)** pDCs over the 72 h study period. Fold change in CD86 MFI on **(E)** CD14 + CD16- monocytes, **(F)** CD14 + CD16 + non-classical monocytes, **(G)** cDCs, and **(H)** pDCs over the 72 h study period.

A decrease in circulating lymphocytes resulted after traumatic injury, and TXA administration did not change this effect (Figure 4). CD4^+^ T cell frequencies were similar between treatment groups at T0 (Figure 4A), and by T6 had decreased roughly 50% in all three treatment groups (Figure 4B). This reduction was maintained throughout the study period of 72 h (Figure 4B). CD8^+^ T cells phenocopied CD4^+^ T cells, such that there was a significant reduction (>50%) in CD8^+^ T cell frequencies by T6, and this was similar between all treatment groups, and maintained through the duration of the study period (Figures 4C,D). Natural killer (NK) cell frequencies were similar between treatment groups at T0, and by T6, NK cell frequencies were reduced by roughly 80% (Figures 4E,F). These profiles were similar between all three treatment groups at T6, T24, and T72. Lastly, while B cell frequencies were reduced from T0 to T72 (Figures 4G,H), the reduction was not as severe as seen in both T and NK cell compartments, and TXA administration did not significantly alter B cell frequencies. In the first 24 h, there was an increase in the frequency of circulating cDC, yet by 72 h post treatment cDC levels had decreased to below baseline (T0) levels (Figures 5A,B). There were no statistically significant differences in cDC frequencies between treatment groups. Plasmacytoid dendritic cell (pDC) populations were also reduced by roughly two thirds within 6 h from baseline [placebo, 0.34 (0.15–0.58); 2 g TXA, 0.23 (0.14–0.45); 4 g TXA, 0.30 (0.14–0.56)], mimicking the pattern seen in the T cell and NK cells compartments and with little difference between placebo and TXA groups (Figures 5C,D). Interestingly, we found a global reduction in HLA-DR on B cells, CD14^+^CD16^+^ monocytes, cDCs, and pDCs (Figures 6A–D) – similar to the phenotype of CD14^+^CD16^–^ monocytes seen in Figure 2. This reduction on HLA-DR was independent of TXA dose. In contrast, CD86 expression (MFI) was relatively maintained over the 72 h study period, with only CD14^+^CD16^–^ monocytes reducing CD86 levels by roughly half at T6 and T24, but returning to T0 levels by T72 (Figures 6E–H).

**FIGURE 7:**
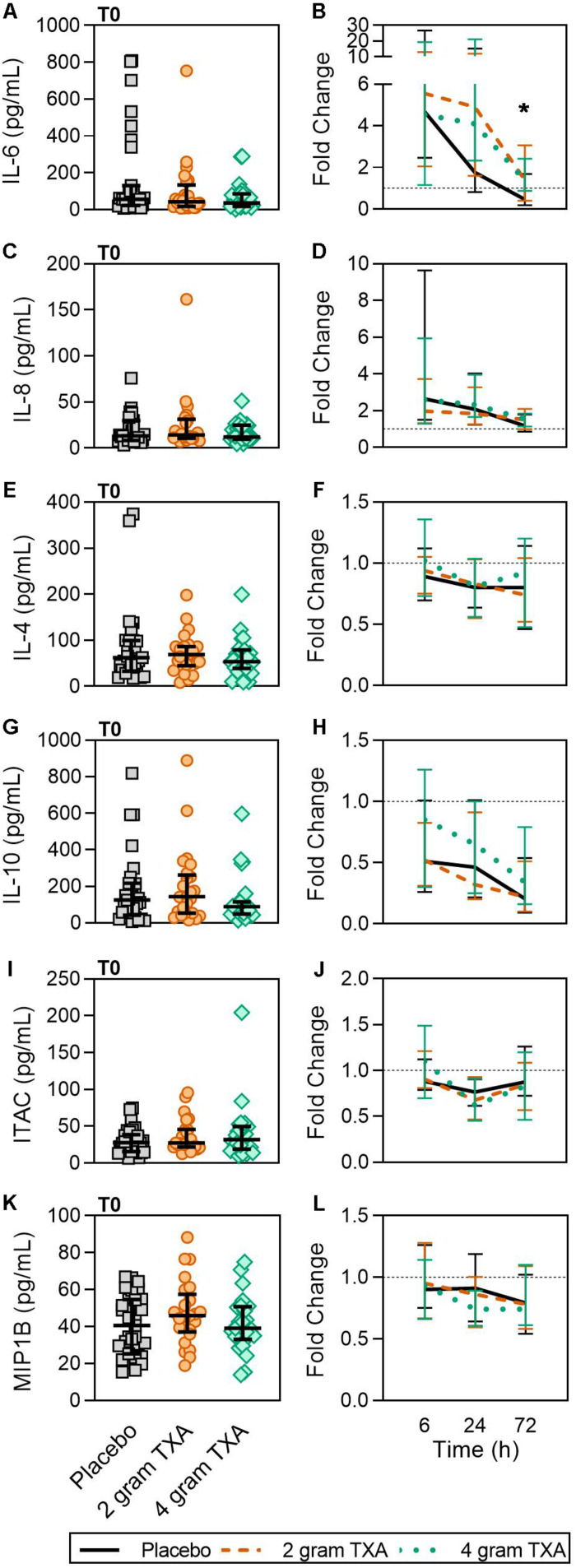
TXA administration increases circulating levels of IL-6. For T0, data are represented as individual points with median + IQR for error bars. For all kinetic data, fold change was calculated as [(frequency/MFI/concentration of a given population/analyte at the given time | | T6,T24,T72| |) ÷ (frequency/MFI/concentration of the same population/analyte at T0)]. Data are displayed as line graphs (fold change, median + IQR). The dashed line placed at *y* = 1 represents T0, or baseline. Placebo, *n* = 29; 2 g TXA, *n* = 24; 4 g TXA, *n* = 25. Statistical differences between the 4 g TXA and placebo groups at each given time point, as calculated via Kruskal-Wallis, are noted as follows: *p* < 0.05, *; *p* > 0.05, not significant and not denoted on the graphical representation. **(A)** IL-6 levels at T0 in all three treatment groups. **(B)** Fold change in IL-6 levels over the study period of 72 h. **(C)** IL-8 levels at T0 in all three treatment groups. **(D)** Fold change in IL-8 levels over the study period of 72 h. **(E)** IL-4 levels at T0 in all three treatment groups. **(F)** Fold change in IL-4 levels over the study period of 72 h. **(G)** IL-10 levels at T0 in all three treatment groups. **(H)** Fold change in IL-10 levels over the study period of 72 h. **(I)** Interferon-inducible T-cell alpha chemoattractant (ITAC/CXCL11) levels at T0 in all three treatment groups. **(J)** Fold change in ITAC levels over the study period of 72 h. **(K)** MIP1B levels at T0 in all three treatment groups. **(L)** Fold change in MIP1B levels over the study period of 72 h.

Only a small fraction of the cytokines/chemokines assayed for (6 of 21, see “Materials and Methods” section for full listing) were found to be substantially altered from the baseline timepoint (T0). IL-6 initially increased by ∼5-fold at 6 h after baseline, then decreased until 72 h post administration for all treatment groups (Figure 7A). Notably, the fold decrease in IL-6 was significantly less in the 4 g TXA group [1.36 (0.87–2.42)] compared to the placebo group [0.46 (0.19–1.69)] at the 72 h time point (*p* = 0.028; Figure 7B). While there were some differences in absolute concentrations at certain time points, (Supplementary Table S5), none of the other cytokines evaluated had a significant fold change between study groups (data not shown). Lastly, we found no differences in overall complement activation between treatment groups; however, by T72, there was an approximate doubling in classical complement activation (as determined by CH50) in all three treatment groups Supplementary Figure S1).

Our results also indicate that a 4 g dose of TXA was associated with a relative reduction in neutrophil expression of CD62L (Figure 3), which is important for neutrophil adhesion and migration across endothelium (54, 55). CD62L keeps neutrophils in the circulation as they cannot egress (56), and is known to be shed almost instantaneously upon cellular activation (54, 55). As CD62L levels in our study did not significantly differ until 24 h post-TXA, and we did not measure shed/soluble CD62L, we cannot directly determine whether the TXA-induced differences in CD62L were indicative of neutrophil activation. One study in healthy humans receiving i.v. bolus injections of lipopolysaccharide (LPS, 4 ng/kg) established that a 2 g bolus of TXA 30 min prior to LPS injection did not alter neutrophil activation, CD62L expression, or degranulation (57). In contrast, other studies in trauma populations have demonstrated that CD62L expression is decreased in response to traumatic injury alone. In 2017, Hazeldine et al. found CD62L expression was decreased by 15% at 72 h after traumatic injury (52). More recently, machine learning was used to identify molecular signatures associated with MODS in traumatically injured patients. In this study, they found that decreases in CD62L expression on fMLF-activated neutrophils, neutrophil CD63 expression, and monocyte frequency were associated with MODS development (58). These data again highlight that the immune modulating effects of TXA are most likely highly dependent on disease etiology and timing of TXA administration (pre- or post-insult). Moreover, assessment of neutrophil function, such as respiratory burst in response to priming and degranulation, would be more informative regarding the true immunomodulatory nature of TXA.

Our results revealed increased plasma levels of IL-6 at 72 h post TXA administration compared to placebo (Figure 7). A similar increase in inflammatory markers was found in orthopedic surgery patients receiving TXA during active bleeding (38). In cardiac surgery patients receiving TXA prophylactically, Draxler et al. demonstrated that IL-6 levels were similar between placebo and TXA treated patients on post-operative day 1 (40). These data suggest that both the baseline immunologic state of the patient and timing of TXA administration may dictate TXA-mediated IL-6 induction or suppression. For example, when TXA is given prophylactically, before bleeding occurs, the patient is at less risk of being in a hyperinflammatory state, and leukocytes are not “primed” to respond to a second stimulus. Conversely, when TXA is given to an actively bleeding patient, the patient’s immune system is activated and may respond with increased IL-6 production in this setting.

**FIGURE 8:**
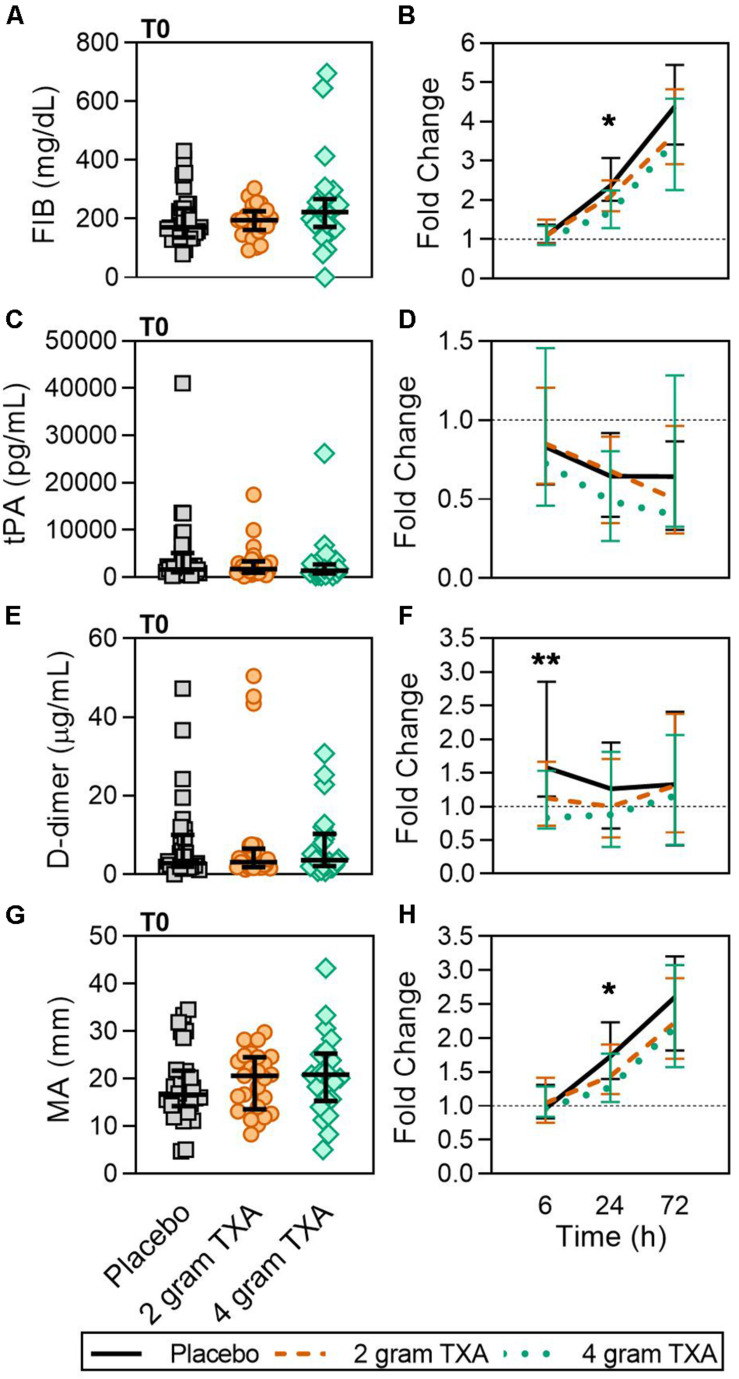
A higher dose of TXA decreases circulating D-dimer levels early after administration. For T0, data are represented as individual points with median + IQR for error bars. For all kinetic data, fold change was calculated as [(frequency/MFI/concentration of a given population/analyte at the given time | | T6,T24,T72| |) ÷ (frequency/MFI/concentration of the same population/analyte at T0)]. Data are displayed as line graphs (fold change, median + IQR). The dashed line placed at *y* = 1 represents T0, or baseline. Placebo, *n* = 29; 2 g TXA, *n* = 24; 4 g TXA, *n* = 25. Statistical differences between the 4 g TXA and placebo groups at each given time point, as calculated via Kruskal-Wallis, are noted as follows: *p* < 0.01, **; *p* < 0.05, *; *p* > 0.05, not significant and not denoted on the graphical representation. **(A)** Fibrinogen (FIB, mg/dL) levels at T0 in all three treatment groups (placebo, *n* = 31; 2 g TXA, *n* = 25; 4 g TXA, *n* = 25). **(B)** Fold change in FIB levels over the study period of 72 h (placebo, *n* = 17; 2 g TXA, *n* = 13; 4 g TXA, *n* = 10). **(C)** Tissue plasminogen activator (tPA, pg/mL) levels at T0 in all three treatment groups (placebo, *n* = 31; 2 g TXA, *n* = 25; 4 g TXA, *n* = 25). **(D)** Fold change in tPA levels over the study period of 72 h (placebo, *n* = 28; 2 g TXA, *n* = 24; 4 g TXA, *n* = 20). **(E)** D-dimer (D-Di, μg/mL) levels at T0 in all three treatment groups (placebo, *n* = 31; 2 g TXA, *n* = 25; 4 g TXA, *n* = 25). **(F)** Fold change in D-Di levels over the study period of 72 h (placebo, *n* = 30; 2 g TXA, *n* = 25; 4 g TXA, *n* = 25). **(G)** Maximum amplitude (MA, mm) levels at T0 in all three treatment groups (placebo, *n* = 30; 2 g TXA, *n* = 25; 4 g TXA, *n* = 22). **(H)** Fold change in MA over the study period of 72 h (placebo, *n* = 30; 2 g TXA, *n* = 23; 4 g TXA, *n* = 19).

Tranexamic acid inhibits plasminogen activation and plasmin activity to prevent clot lysis. In our trial, there were clinically relevant differences in D-dimer concentrations at 6 h (Figure 8 and Supplementary Table S3), reflecting the antifibrinolytic effect of TXA between study groups. Fibrinogen concentrations between study groups at 24 h were statistically different, but the differences do not appear to be clinically significant upon examination of the absolute values for each of these parameters (Figure 8 and Supplementary Table S3). Measures of procoagulant activity, including thrombin-antithrombin complex (TAT), prothrombin fragment 1 and 2 (PF 1 and 2), and viscoelastic testing with thromboelastography (TEG) clot time (R time) and plasma clot strength were all similar between groups. While the median fold change in maximum amplitude, or MA, in the 4 g TXA group was significantly decreased compared to the placebo group at 24 h post-TXA (Figure 8), the median MA values at 24 h were not significantly different between groups. The transiently lower MA in the 4 g TXA group may be explained by the lesser increase in FIB at 24 h. Lastly, there was a trend that approached significance regarding the incidence of TE with respect to TXA dose. Previous trials of TXA use in trauma populations have not reported an increased risk of TE (14, 15). In contrast to our study, these trials did not use active Duplex screening for TE, thereby potentially underreporting TE events. However, our results are consistent with the HALT-IT study, an RCT on TXA use in 12,000 adults with gastrointestinal bleeding that reported an increase in TE when TXA is used in this population (59).

Endothelial effects of TXA have been examined in *in vitro* models of reperfusion injury indicating preservation of the glycocalyx (60, 61). The field of clinical monitoring of endothelial dysfunction has been limited to assessing reactive hyperemia (62), therefore the use of SDC1 as a potential biomarker of endothelial injury has been of great interest. Our study indicates that there was no difference in circulating VWF (a sign of endothelial activation and Weibel-Palade body mobilization) or SDC1 (shed in response to endothelial activation or damage) concentrations between TXA-treated and placebo groups during the 72 h post TXA administration (data not shown, Supplementary Table S6). Lastly, there were no differences in mortality among treatment groups, although there was a dose-dependent numerical reduction in 28-day mortality for patients treated with TXA. Previous studies reporting reduced mortality when TXA was administered after severe bleeding included far more patients than our study (14, 63, 64).

This trial is the first trauma-based RCT to have blood drawn prior to TXA administration, and as such provides a unique insight into the immune and hemostatic profiles early after trauma but prior to TXA administration. However, there are several limitations of this trial. First, it was not possible to assess leukocyte function by *ex vivo* stimulation, cytokine production, migration, or other functional readouts due to the lack of 24 h availability of laboratory-based research staff. This would have allowed for a more thorough analysis of the immune consequences of TXA in this population. Second, the sample size of the trial was inadequate to assess sex as a biological variable, as well as effects on clinical outcomes such as organ failure and mortality. Third, the high frequency of penetrating injury may reduce generalization of these results to populations with blunt injury. Fourth, ICU free days were calculated using a 30 day model, even though follow-up was only through hospital discharge. Fifth, the inability to serially obtain viscoelastic measures at this trauma center from WB samples impaired the ability to examine hemostatic function in full. Lastly, we were only able to measure classical complement activation, and not activation of the alternative or lectin pathways.

We assessed multiple hemostatic parameters between the study groups. Of all of the measurements performed, we found a small number of significant and clinically relevant differences related to TXA dosing. While fibrinogen (FIB) concentrations were similar between groups at T0 (Figure 8A), by 24 h post-treatment the fold change in FIB was significantly reduced in the 4 g TXA study group [1.69 (1.29–2.25)], compared to placebo [2.38 (1.98–3.07)] (*p* = 0.023; Figure 8B). Moreover there was a marked roughly 4-fold increase in FIB over the entire study period for all three treatment groups (Figure 8B). We found no difference in tPA between groups at T0, but that overall there was an approximate 50% reduction in tPA levels over the course of the study period, no matter the treatment group (Figures 8C,D). The fold change seen in D-dimer levels was significantly smaller in the 4 g TXA study group [0.83 (0.68–1.53)] compared to the placebo group [1.58 (1.15–2.86)] at T6 (*p* = 0.006; Figures 8E,F). We found that the clot maximum amplitude (MA), as measured in PPP by TEG, was no different between treatment groups at T0 (Figure 8G). By T24, the placebo group had a greater increase in MA as demonstrated by a 1.73-fold increase (Q1-Q3, 1.40–2.23) in MA from T0, while the 4 g TXA group had only a 1.27-fold increase (1.06–1.77) from T0 (*p* = 0.048, Figure 8H). However, the median MA values at 24 h were not significantly different between groups [placebo, 31.9 (27.5–35.4); 2 g TXA, 30.25 (24.8–33.6); 4 g TXA, 28.4 (22.2–32.8); *p* = 0.283]. There were no additional differences in hemostatic and endothelial parameters measured between treatment groups (Supplementary Table S6).

**FIGURE 9:**
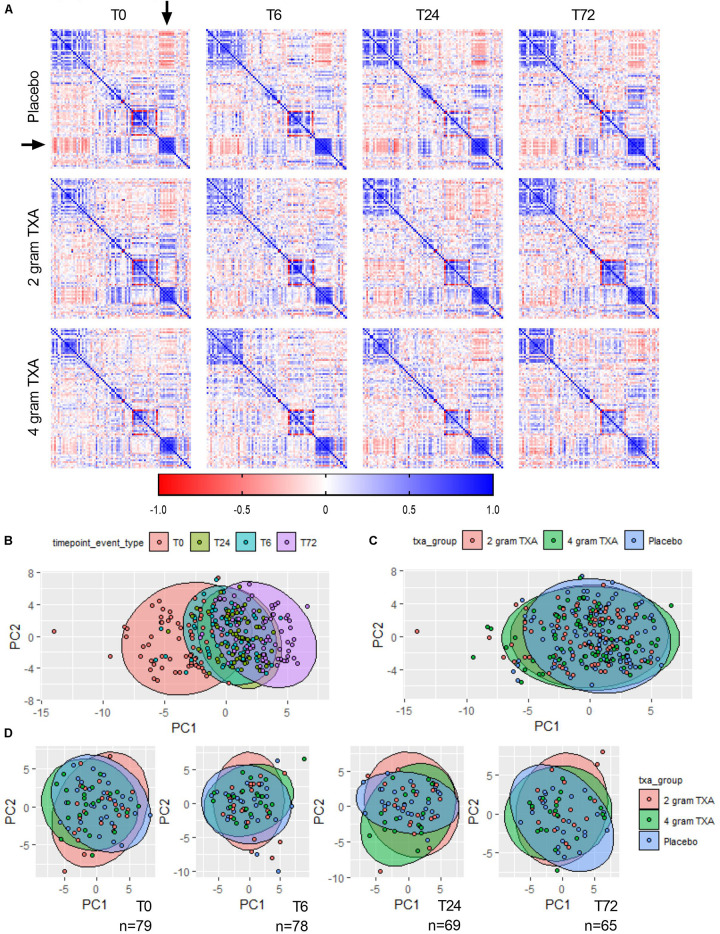
The effects of trauma, and not of TXA administration, impart the greatest impact on immune and hemostatic phenotypes in traumatically injured bleeding patients. **(A)** Correlation matrices at each time point for each study group were built using the variables listed in Supplementary Table S7. Positive correlation is denoted in blue, while a negative correlation is denoted in red. Principal component analysis (PCA) labeled by **(B)** sampling time point (“timepoint_event_type”; T0, T6, T24, and T72; *n* = 283), **(C)** treatment group (“txa_group”; Placebo, 2 g TXA, 4 g TXA; *n* = 283), or **(D)** at a given time point by treatment group. For PCA, confidence ellipses are set at 95%.

Due to the comprehensive immune and hemostatic phenotyping performed in this trial, we leveraged the entire data set to see if we could identify signatures or trends that were associated with TXA administration. We used all of the variables listed in Supplementary Table S7 to generate correlation matrices and subsequent heat maps (Figure 9A). We found that in patients receiving placebo, the MFIs of CD86 and HLA-DR on B cells, monocytes (CD14^+^CD16^–^ and CD14^+^CD16^+^) and DCs inversely correlated with the circulating levels of GM-CSF, Fractalkine, IFNγ, IL-12p70, IL-17a, IL-1β, IL-2, IL-21, IL-23, IL5, and IL-7 (arrowheads, Figure 9A, Supplementary Figure S2). However, this correlation was diminished in patients receiving 2 g or 4 g doses of TXA at early time points (T0, T6). We also performed principal component analysis (variables used in Supplementary Table S8) to determine the greatest source of variance in our dataset. We first found that the kinetics of trauma played an important role in dictating the immune and hemostatic phenotype, as indicated by the time point clustering seen in Figure 9B. Alternatively, the variance in the dataset could not be explained by the use of TXA, as the 95% confidence bands for each group in Figure 9C are relatively overlapping. We subsequently examined TXA use at each distinct time point, as time itself proved to impart a large degree of variance. We observed again that the use of TXA was not informative as to the variance in the immune and hemostatic profiles in these trauma patients (Figure 9D).

## Discussion

Our trial was designed to determine the immunologic effects of two different i.v. bolus doses of TXA in patients with severe traumatic injury. This RCT is novel as it provides early (<2 h from injury) immune phenotype information in traumatically injured patients, and immune and hemostatic phenotyping for up to 72 h following administration of TXA or placebo. Our results indicate limited immunomodulatory effects of TXA during the 72 h period post-treatment (as determined by the phenotypic metrics we were able to assess), and reduced fibrinolysis in response to higher doses of TXA. There were no differences between treatment groups in biomarkers of endothelial activation, the amount of blood products transfused post-intervention, the incidence of MODS, or mortality. Plasmin has been shown to have multiple effects on hemostatic, immune, and endothelial function. However, our data demonstrate that the immune and hemostatic phenotype induced by traumatic injury greatly outweighs changes in function due to plasmin inhibition by TXA.

The data from our trial indicates that HLA-DR expression on monocytes was reduced approximately 40% (Figure 2), and this was irrespective of the treatment group. TXA inhibition of plasminogen has been shown to reduce the activation of monocytes, neutrophils, and complement in animal trauma models (50, 51). There is also evidence that when TXA is used prophylactically for patients requiring cardiac surgery, it enhances immune function, as evidenced by decreased immune suppression and post-surgical infection rates (40). These opposing data suggest that there may be a temporal and a disease etiology component to TXA function, both of which dictate the degree of the immune modulating effects of TXA. Draxler et al. showed that in healthy volunteers, HLA-DR expression was reduced on monocytes 24 h after TXA administration (40). With no underlying pathophysiology to bias the immune status, perhaps it is easier to see the differences TXA can impart on immune phenotype. However, after traumatic injury, there is a dramatic reduction in HLA-DR expression on monocytes (52, 53). Moreover, our data show that there is a global reduction in HLA-DR expression in response to traumatic injury, irrespective of immune cell type (Figure 6), and that time after injury and not TXA dose is responsible for variability in patient immune phenotypes (Figure 9). Collectively, this evidence suggests that perhaps the phenotypes incurred by trauma are more severe, and outweigh any immune phenotype that would be induced by TXA administration.

## Conclusion

In conclusion, in this RCT in patients with primarily penetrating traumatic injuries, 2 and 4 g i.v. bolus dosing of TXA had minimal immunomodulatory and hemostatic effects.

## Data Availability Statement

The datasets presented in this article are not readily available because, individual participant data, including data dictionaries, will be made available to the community. These data are to include the individual participant data collected during the trial, after de-identification. The study protocol, statistical analysis plan, and human protections information are all available in the Supplementary Material. Data will be made available beginning 9 months and ending 36 months following article publication for anyone who wishes to access the data. To gain access, data requestors will need to sign a data access agreement. Data will be available at Mendeley Data (link will be provided upon completion of data access agreement). There will be no available biospecimens associated with this clinical trial. Requests to access the datasets should be directed to Philip C. Spinella.

## Ethics Statement

The studies involving human participants were reviewed and approved by the United States Army Medical Research and Materiel Command, Human Research Protections Office Washington University in St. Louis Institutional Review Board, and US FDA IND 125420. Written informed consent for participation was not required for this study in accordance with the national legislation and the institutional requirements.

## Author Contributions

PS designed the study, analyzed the data, provided funding and reagents, and wrote the manuscript. KT conducted the experiments, acquired and analyzed the data, and wrote the manuscript. IT helped to design the study, conducted the experiments, acquired and analyzed the data, and edited the manuscript. AF, SS, and MM conducted the experiments, acquired and analyzed the data, and edited the manuscript. KB designed the study, supervised the implementation of the trial, and edited the manuscript. DS and SR supervised the implementation of the trial and edited the manuscript. AC and CH acquired and analyzed the data, and edited the manuscript. JB managed the database and performed statistical analyses, and edited the manuscript. AP helped to design the study, analyzed the data, and edited the manuscript. JL designed the study, analyzed the data, and edited the manuscript. AC and GB designed the study, analyzed the data, provided funding and reagents, and edited the manuscript. All authors contributed to the article and approved the submitted version.

## Disclaimer

The opinions or assertions contained herein are the private views of the author and are not to be construed as official or as reflecting the views of the Department of the Army or the Department of Defense.

## Conflict of Interest

All authors report grant support from the United States Department of Defense during the conduct of this study. JL reports personal fees from CSL Behring, Instrumentation Labs, Janssen, Leading Biosciences, and Octapharma outside the submitted work.

## Abbreviations

CRASH-2: clinical randomization of an antifibrinolytic in significant hemorrhage
fMLF: N-formylmethionyl -leucyl-phenylalanine
IDS: investigational drug service
MA: maximal amplitude
RBC: red blood cells
RCT: randomized controlled trial
SAS: statistical analysis software
TBI: traumatic brain injury
TIC: trauma induced coagulopathy
TXA: tranexamic acid
Wash U: Washington University in St. Louis
WB: whole blood.
