# Supplementary material for: The Immunologic Effect of Early Intravenous Two and Four Gram Bolus Dosing of Tranexamic Acid Compared to Placebo in Patients With Severe Traumatic Bleeding (TAMPITI): A Randomized, Double-Blind, Placebo-Controlled, Single-Center Trial
Source: Front Immunol. 2020 Sep 8;11:2085. doi: 10.3389/fimmu.2020.02085 (PMC7506112; doi:10.3389/fimmu.2020.02085)
Supplement: Supplementary file 1 [file Table_1.DOCX]

*The immunologic effect of early intravenous two and four gram bolus dosing of tranexamic acid compared to placebo in patients with severe traumatic bleeding (TAMPITI): a randomized, double-blind, placebo-controlled, single-center trial*

Spinella et al.

**Supplementary Information**

**Trial Registration**

Trial Registration Number: NCT02535949

**Conflict of Interest/Disclosure Statements**

ICMJE disclosure statements from each author can be found at the end of this document in Appendix B.

**CONSORT Checklist**

| Section/Topic | Item No | Checklist item | Reported on page No |
| --- | --- | --- | --- |
| Title and abstract | | | |
|  | 1a | Identification as a randomised trial in the title | 1 |
|  | 1b | Structured summary of trial design, methods, results, and conclusions (for specific guidance see CONSORT for abstracts) | 2 |
| Introduction | | | |
| Background and objectives | 2a | Scientific background and explanation of rationale | 3 |
|  | 2b | Specific objectives or hypotheses | 3 |
| Methods | | | |
| Trial design | 3a | Description of trial design (such as parallel, factorial) including allocation ratio | 3-4 |
|  | 3b | Important changes to methods after trial commencement (such as eligibility criteria), with reasons | n/a |
| Participants | 4a | Eligibility criteria for participants | 3-4 |
|  | 4b | Settings and locations where the data were collected | 3-4 |
| Interventions | 5 | The interventions for each group with sufficient details to allow replication, including how and when they were actually administered | 4 |
| Outcomes | 6a | Completely defined pre-specified primary and secondary outcome measures, including how and when they were assessed | 4-5 |
|  | 6b | Any changes to trial outcomes after the trial commenced, with reasons | n/a |
| Sample size | 7a | How sample size was determined | 5-6 |
|  | 7b | When applicable, explanation of any interim analyses and stopping guidelines | 5-6 |
| Randomisation: |  |  |  |
| Sequence generation | 8a | Method used to generate the random allocation sequence | 4 |
|  | 8b | Type of randomisation; details of any restriction (such as blocking and block size) | 4 |
| Allocation concealment mechanism | 9 | Mechanism used to implement the random allocation sequence (such as sequentially numbered containers), describing any steps taken to conceal the sequence until interventions were assigned | 4 |
| Implementation | 10 | Who generated the random allocation sequence, who enrolled participants, and who assigned participants to interventions | 4 |
| Blinding | 11a | If done, who was blinded after assignment to interventions (for example, participants, care providers, those assessing outcomes) and how | 4 |
|  | 11b | If relevant, description of the similarity of interventions | n/a |
| Statistical methods | 12a | Statistical methods used to compare groups for primary and secondary outcomes | 5-6 |
|  | 12b | Methods for additional analyses, such as subgroup analyses and adjusted analyses | 5-6 |
| Results | | | |
| Participant flow (a diagram is strongly recommended) | 13a | For each group, the numbers of participants who were randomly assigned, received intended treatment, and were analysed for the primary outcome | 6 and Figure 1 |
|  | 13b | For each group, losses and exclusions after randomisation, together with reasons | 6 |
| Recruitment | 14a | Dates defining the periods of recruitment and follow-up | 6 |
|  | 14b | Why the trial ended or was stopped | 4 |
| Baseline data | 15 | A table showing baseline demographic and clinical characteristics for each group | Table 1 |
| Numbers analysed | 16 | For each group, number of participants (denominator) included in each analysis and whether the analysis was by original assigned groups | 6-7, Tables 1 and 2 |
| Outcomes and estimation | 17a | For each primary and secondary outcome, results for each group, and the estimated effect size and its precision (such as 95% confidence interval) | 6-7 |
|  | 17b | For binary outcomes, presentation of both absolute and relative effect sizes is recommended | n/a for primary outcome |
| Ancillary analyses | 18 | Results of any other analyses performed, including subgroup analyses and adjusted analyses, distinguishing pre-specified from exploratory | n/a |
| Harms | 19 | All important harms or unintended effects in each group (for specific guidance see CONSORT for harms) | 6-7 |
| Discussion | | | |
| Limitations | 20 | Trial limitations, addressing sources of potential bias, imprecision, and, if relevant, multiplicity of analyses | 10 |
| Generalisability | 21 | Generalisability (external validity, applicability) of the trial findings | 10 |
| Interpretation | 22 | Interpretation consistent with results, balancing benefits and harms, and considering other relevant evidence | 10 |
| Other information | | |  |
| Registration | 23 | Registration number and name of trial registry | 4 |
| Protocol | 24 | Where the full trial protocol can be accessed, if available | Supple-ment Appendix A |
| Funding | 25 | Sources of funding and other support (such as supply of drugs), role of funders | 11 |

**Supplementary Methods**

Blood samples for immunophenotyping were collected in K_2_EDTA Vacutainer Blood Collection Tubes (#367863, Beckton Dickinson, Franklin Lakes, NJ), transported to the laboratory at room temperature (RT), and processed as indicated in the section labeled "Immunophenotyping". Blood samples for hemostatic, endothelial, and cytokine assays were collected in buffered sodium citrate (9NC) blood collection tubes (#369714, #363083, Beckton Dickinson) and transported to the laboratory on ice. For platelet poor plasma (PPP) isolation, whole blood (WB) was centrifuged at 3000*g* for 20 min 4°C. PPP aliquots were stored at -80°C prior to analysis.

For immunophenotyping, blood was collected in K_2_EDTA Vacutainer Blood Collection Tubes. Blood was fixed within two hours of sample collection by mixing 1·25 mL whole blood (WB) with 1·75 mL Proteomic Stabilizer (Smart Tube Inc., San Carlos, CA, US) and incubating for 10 minutes at room temperature (RT). Subsequently, samples were frozen at -80°C. Flow cytometric immunophenotyping was performed in batch analysis, processing samples from all four time points (T0, T6, T24, T72) of two to four patients simultaneously. Samples were thawed, subjected to red blood cell (RBC) lysis and washing steps per manufacturer's recommendations. Cells were incubated with Fc receptor blocking reagent (TruStain FcX, Biolegend, San Diego, CA, US) and stained with fluorochrome-conjugated antibodies (panels 1, 2, and 3; see supplemental table 2). Samples were acquired on an LSR Fortessa using Diva software (both from BD Biosciences, San Jose, CA, US) and analyzed with the FlowJo v10·2 software (Treestar, Ashland, OR, US). Analysis of the individual peripheral blood leukocyte subsets was done as specified in supplemental table 3. Cell concentrations in stained samples were determined by adding counting beads to the samples immediately before flow cytometric acquisition (123count eBeads, eBioscience, San Diego, CA, US). Cell concentrations in samples were calculated per manufacturer's directions and were used to determine the cell counts per mL of WB.

Code used for PCA:

library(prcomp)

myPr<- prcomp(data[,4:85], scale = TRUE)

summary(myPr)

plot(myPr, type = "l")

biplot(myPr, scale = "0")

str(myPr)

myPr$x

dataPr<- cbind(data, myPr$x[,1:2])

library(ggplot2)

ggplot(dataPr, aes(PC1, PC2, col = timepoint_event_type, fill = timepoint_event_type)) +

stat_ellipse(geom = "polygon", col = "black", alpha = 0.5) +

geom_point(shape = 21, col = "black")

ggplot(dataPr, aes(PC1, PC2, col = txa_group, fill = txa_group)) +

stat_ellipse(geom = "polygon", col = "black", alpha = 0.5) +

geom_point(shape = 21, col = "black")

**Supplementary Acknowledgments**

*TAMPITI Trial Investigators:* Obeid Ilahi, M.D.; Stephen Jarman, R.N., B.S.N.; John Kirby, M.D.; Tiffany Osborn, M.D., M.P.H.; Bryan Sato, R.N., B.S.N.; Eden Nohra, M.D.; Laurie Punch, M.D.; Jarot Guerra, M.D.; Maya Sorini, B.S.; James McMullen, B.S.; Nicholas Fiore, B.S.; Rohit Rasane, M.B.B.S.; Brendan Wesp, B.S.; Jason Snyder, M.D.; Qiao Zhang, M.S.; Shin-Wen Hughes, B.S.; Sarbani Ghosh, M.H.S.

**Supplementary Figures**

**Supplementary Figure 1. Complement Activity (CH50) in treatment groups over the study period.** For T0, data are represented as individual points with median + IQR for error bars. For all kinetic data, fold change was calculated as [(frequency/MFI/concentration of a given population/analyte at the given time ||T6,T24,T72|| ) ÷ (frequency/MFI/concentration of the same population/analyte at T0)]. Data are displayed as line graphs (fold change, median + IQR). The dashed line placed at y=1 represents T0, or baseline. Placebo, n=30; 2g TXA, n=25; 4g TXA, n=25. Statistical differences between the 4g TXA and placebo groups at each given time point, as calculated via Kruskal-Wallis, are noted as follows: p<0.01, ** ; p<0.05, * ; p>0.05, not significant and not denoted on the graphical representation. (A) CH50 levels at T0 in all three treatment groups. (B) Fold change in CH50 over the study period of 72 hours.

**Supplementary Figure 2. Heat Map labels.** Each row and column in the correlation matrix is labeled with the variable used for creating the matrix. Full definitions of each label can be found in Supplemental Table 7.

**
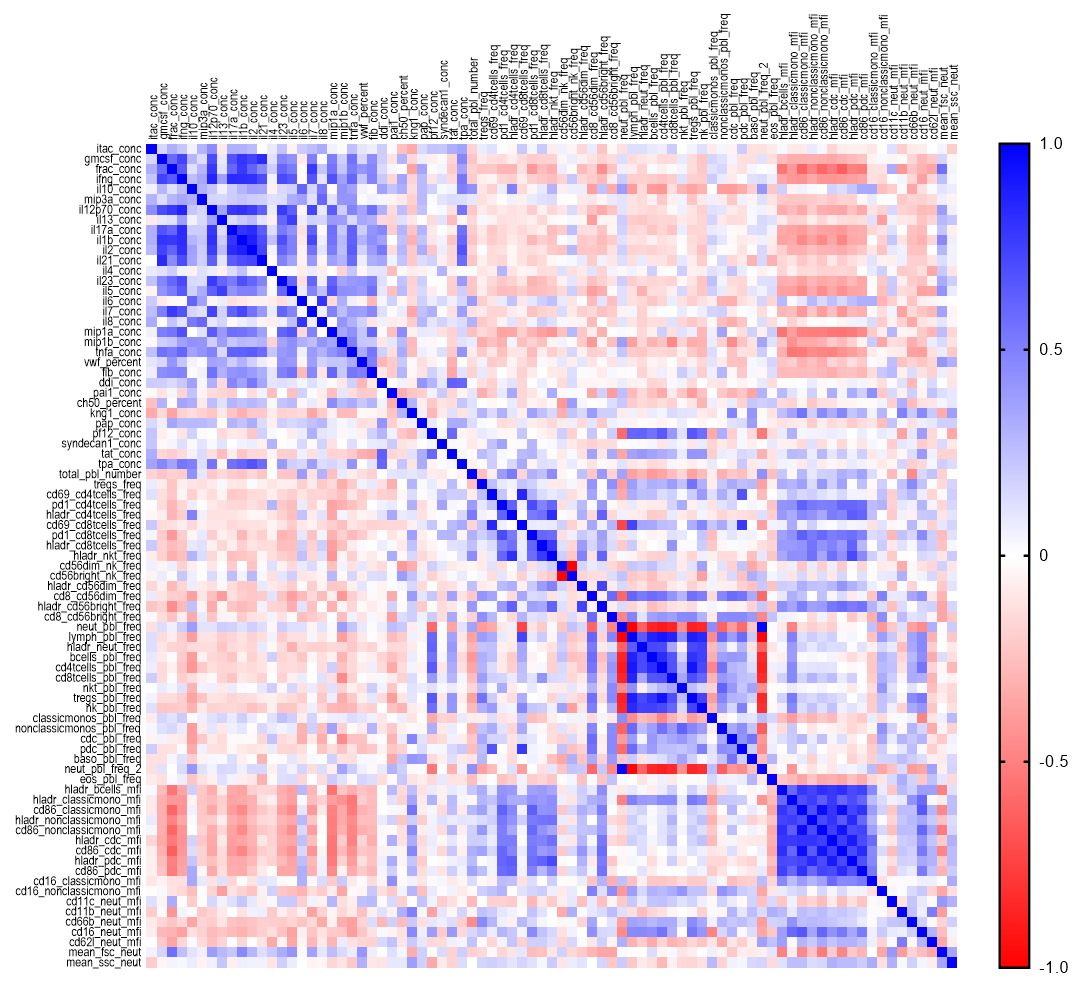
**

**Supplementary Tables**

**Supplementary Table 1. Exclusion Criteria.** Past medical history of hemorrhagic stroke is permitted, but not current admission with hemorrhagic stroke. Abbreviations: DVT, deep vein thrombosis; PE, pulmonary embolism; MI, myocardial infarction; ED, emergency department.

| **All Possible Exclusion Criteria** |
| --- |
| Patients known to be < 18 years of age |
| Suspected acute MI or stroke (thromboembolic and/or hemorrhagic) on admission |
| Known inherited coagulation disorders |
| Known past medical history of thromboembolic events (DVT, PE, MI, Thromboembolic Stroke) |
| Known history of seizures and/or seizure after injury/on admission related to this hospitalization |
| Suspected or known pregnancy |
| Futile care |
| Known current state of immunosuppression (i.e. on high dose steroids, chemotherapeutics, etc.) |
| Unknown estimated time of injury |
| Patients wearing an “Opt Out” TAMPITI Study bracelet |
| Known presence of subarachnoid hemorrhage |
| Isolated injuries to hands and/or feet (distal) |
| Administration of antifibrinolytics pre-hospital and/or during this ED admission prior to enrollment |
| **Top 10 Exclusion Criteria Used** |
| No Blood Products or Transfer to the Operating Room, n = 629 |
| Time of Injury > 2h, n = 183 |
| Futile Care, n = 45 |
| Unknown Time of Injury, n = 25 |
| Subarachnoid Hemorrhage, n = 21 |
| Known History of Seizure, n = 15 |
| Known Prisoner, n = 9 |
| Minor, n = 4 |
| Known Pregnancy, n = 3 |
| Known History of Stroke, n = 3 |

**Supplementary Table 2. Antibodies used for identification of leukocyte subsets within peripheral blood.** AF, Alexa Fluor; APC, allophycocyanin; BV, Brilliant Violet; PE, phycoerythrin; PerCP, Peridinin-Chlorophyll-Protein.

| **Marker** | **Fluorochrome** | **Clone** | **Supplier** |
| --- | --- | --- | --- |
| *Panel 1: T, B, NK cells* | | | |
| CD3 | PerCP-Cy5.5 | UCHT1 | BD Biosciences |
| CD4 | FITC | SK3 | Biolegend |
| CD8 | AF700 | HIT8a | Biolegend |
| CD19 | PE/Dazzle 594 | HIB19 | Biolegend |
| CD25 | PE-Cy7 | 2A3 | BD Biosciences |
| CD127 | BV605 | A019D5 | Biolegend |
| CD56 | PE | HCD56 | Biolegend |
| CD69 | APC | FN50 | Biolegend |
| HLA-DR | BV510 | L243 | Biolegend |
| PD-1 | BV421 | EH12.2H7 | Biolegend |
| *Panel 2: Monocytes, DCs* | | | |
| CD3 | PerCP-Cy5.5 | UCHT1 | BD Biosciences |
| CD19 | PerCP-Cy5.5 | HIB19 | BD Biosciences |
| CD14 | BV605 | M5E2 | Biolegend |
| CD15 | PE/Dazzle 594 | W6D3 | Biolegend |
| CD16 | FITC | 3G8 | Biolegend |
| HLA-DR | BV510 | L243 | Biolegend |
| CD11c | AF700 | Bu15 | Biolegend |
| CD86 | BV421 | 2331 | BD Biosciences |
| CD1c | PE-Cy7 | L161 | Biolegend |
| BDCA-2 | PE | 201A | Biolegend |
| CD123 | APC | 6H6 | Biolegend |
| *Panel 3: Granulocytes* | | | |
| CD66b | AF647 | G10F5 | Biolegend |
| CD11b | A700 | M1/70 | Biolegend |
| CD11c | PerCP-Cy5.5 | Bu15 | Biolegend |
| CD14 | BV605 | M5E2 | Biolegend |
| CD16 | FITC | 3G8 | Biolegend |
| CD62L | PE-Cy7 | DREG-56 | Biolegend |
| CD125 | PE | A14 | BD Biosciences |
| HLA-DR | BV510 | L243 | Biolegend |
| CD54 | Pacific Blue | HA58 | Biolegend |

**Supplementary Table 3. Surface markers used to identify immune cell subsets within peripheral blood.**

Abbreviations: Tregs, regulatory T cells; DC, dendritic cell.

| **Subset** | **Surface markers used for identification** |
| --- | --- |
| B cells | CD19^+^ HLA-DR^+^ |
| CD4^+^ T cells | CD19^–^CD56^–^ CD3^+^ CD4^+^ CD8^–^ |
| CD8^+^ T cells | CD19^–^CD56^–^ CD3^+^ CD4^–^ CD8^+^ |
| Tregs | CD19^–^CD56^–^ CD3^+^ CD4^+^ CD8^–^ CD25^+^ |
| NK T cells | CD19^–^CD56^+^ CD3^+^ |
| NK cells | CD19^–^CD56^+^ CD3^–^ |
| Monocytes | CD3^–^ CD19^–^ CD15^–^ CD14^+^ |
| Myeloid (classical) DC | CD3^–^ CD19^–^CD15^–^ CD11c^+^ HLA-DR^hi^ CD1c^+^ |
| Plasmacytoid DC | CD3^–^ CD19^–^CD15^–^ CD123^+^ BDCA-2^+^ |
| Basophils | CD3^–^ CD19^–^CD15^–^ CD123^+^ BDCA-2^–^ |
| Neutrophils | CD14^–^ CD16^+^ CD125^–^ CD11b^+^ CD66b^+^ |
| Eosinophils | CD14^–^ CD16^low^ CD125^+^ |

**Supplementary Table 4. Peripheral blood immunophenotyping data in each study group over time.** Data from all patients with available samples, results at a given time point (T0, T6, T24, T72) between treatment groups (placebo, 2 gram TXA, 4 gram TXA). (A) Frequencies (% of total peripheral blood leukocytes) and (B) numbers (cells/mL) of each subset are reported, with median (Q1-Q3) listed. Kruskal-Wallis test was performed for each subset at the given time point across treatment groups; p < 0.05 was considered significant. Abbreviations: Tregs, regulatory T cells; DC, dendritic cell.

| **Subset** | **Table 4A** | **T0 (Median (IQR))** |  |  |
| --- | --- | --- | --- | --- |
|  | *Placebo* | *2 gram TXA* | *4 gram TXA* | *p-value* |
| *B cells (%)* | 2.03 (1.14-3.07) | 2.19 (1.03-3.16) | 1.80 (1.13-3.11) | 0.988 |
| *CD4^+^ T cells* | 6.45 (4.49-8.14) | 6.32 (4.34-9.71) | 6.79 (5.04-8.87) | 0.690 |
| *CD8^+^ T cells* | 4.21 (2.26-6.39) | 4.37 (2.18-7.26) | 4.23 (2.43-6.19) | 0.892 |
| *Tregs* | 0.36 (0.20-0.57) | 0.41 (0.27-0.73) | 0.34 (0.28-0.53) | 0.439 |
| *NK T cells* | 0.62 (0.18-2.28) | 0.74 (0.31-2.36) | 0.98 (0.40-2.28) | 0.615 |
| *NK cells* | 2.26 (1.32-4.49) | 3.25 (1.75-4.50) | 2.80 (1.07-4.58) | 0.571 |
| *Monocytes* | 4.26 (3.01-5.49) | 4.34 (2.88-5.29) | 3.67 (2.69-4.49) | 0.189 |
| *Myeloid (classical) DC* | 0.04 (0.03-0.08) | 0.05 (0.03-0.10) | 0.04 (0.03-0.08) | 0.834 |
| *Plasmacytoid DC* | 0.09 (0.05-0.15) | 0.09 (0.06-0.18) | 0.10 (0.07-0.14) | 0.654 |
| *Basophils* | 0.16 (0.10-0.27) | 0.19 (0.11-0.29) | 0.16 (0.11-0.27) | 0.866 |
| *Neutrophils* | 71.4 (62.8-79.3) | 72.0 (58.0-79.2) | 72.3 (58.0-78.8) | 0.858 |
| *Eosinophils* | 0.91 (0.58-1.41) | 0.90 (0.57-1.84) | 0.85 (0.56-1.74) | 0.902 |
|  |  | **T6 (Median (IQR))** |  |  |
| *B cells* | 1.05 (0.74-1.58) | 1.19 (0.59-1.87) | 0.99 (0.62-1.74) | 0.867 |
| *CD4^+^ T cells* | 2.71 (1.60-4.96) | 2.00 (1.30-4.45) | 2.33 (1.31-4.18) | 0.671 |
| *CD8^+^ T cells* | 1.27 (0.81-3.16) | 1.40 (0.72-3.11) | 1.25 (0.70-2.25) | 0.581 |
| *Tregs* | 0.20 (0.09-0.38) | 0.15 (0.09-0.28) | 0.14 (0.08-0.25) | 0.450 |
| *NK T cells* | 0.11 (0.02-0.37) | 0.10 (0.06-0.36) | 0.09 (0.04-0.40) | 0.688 |
| *NK cells* | 0.49 (0.27-0.98) | 0.57 (0.34-0.95) | 0.48 (0.27-0.78) | 0.486 |
| *Monocytes* | 5.84 (4.57-6.82) | 5.62 (4.52-7.32) | 5.99 (4.98-7.51) | 0.652 |
| *Myeloid (classical) DC* | 0.05 (0.04-0.10) | 0.05 (0.03-0.08) | 0.06 (0.04-0.11) | 0.723 |
| *Plasmacytoid DC* | 0.03 (0.01-0.05) | 0.03 (0.02-0.05) | 0.03 (0.01-0.04) | 0.638 |
| *Basophils* | 0.05 (0.03-0.09) | 0.07 (0.03-0.12) | 0.04 (0.02-0.08) | 0.456 |
| *Neutrophils* | 85.1 (78.7-87.4) | 83.4 (78.6-87.0) | 82.9 (80.1-87.6) | 0.915 |
| *Eosinophils* | 0.25 (0.11-0.34) | 0.28 (0.17-0.40) | 0.15 (0.08-0.36) | 0.173 |
|  |  | **T24 (Median (IQR))** |  |  |
| *B cells* | 1.57 (1.19-2.08) | 1.83 (0.96-2.72) | 1.97 (1.36-2.80) | 0.081 |
| *CD4^+^ T cells* | 3.70 (2.42-4.55) | 3.13 (2.40-4.50) | 4.70 (2.66-6.90) | 0.064 |
| *CD8^+^ T cells* | 1.89 (1.27-2.98) | 2.13 (1.32-3.14) | 2.48 (1.33-3.81) | 0.345 |
| *Tregs* | 0.27 (0.16-0.41) | 0.23 (0.17-0.42) | 0.33 (0.21-0.50) | 0.139 |
| *NK T cells* | 0.17 (0.04-0.38) | 0.17 (0.09-0.45) | 0.25 (0.08-0.51) | 0.321 |
| *NK cells* | 0.48 (0.29-0.73) | 0.50 (0.35-0.74) | 0.53 (0.27-0.86) | 0.994 |
| *Monocytes* | 6.09 (4.30-7.46) | 5.94 (4.98-7.87) | 6.00 (4.41-7.12) | 0.777 |
| *Myeloid (classical) DC* | 0.07 (0.05-0.11) | 0.06 (0.04-0.08) | 0.06 (0.05-0.12) | 0.594 |
| *Plasmacytoid DC* | 0.03 (0.01-0.05) | 0.03 (0.02-0.05) | 0.04 (0.02-0.05) | 0.244 |
| *Basophils* | 0.12 (0.07-0.19) | 0.09 (0.04-0.19) | 0.10 (0.06-0.17) | 0.683 |
| *Neutrophils* | 81.0 (77.7-84.3) | 82.3 (77.7-84.8) | 79.3 (71.8-84.0) | 0.161 |
| *Eosinophils* | 0.30 (0.16-0.66) | 0.36 (0.18-0.60) | 0.32 (0.11-1.03) | 0.890 |
|  |  | **T72 (Median (IQR))** |  |  |
| *B cells* | 1.20 (0.60-1.70) | 1.35 (1.00-2.10) | 1.36 (0.86-1.92) | 0.314 |
| *CD4^+^ T cells* | 3.23 (1.95-4.55) | 2.99 (2.00-4.58) | 3.27 (2.27-5.92) | 0.793 |
| *CD8^+^ T cells* | 1.44 (0.84-2.42) | 1.51 (0.75-2.68) | 1.55 (0.78-2.60) | 0.955 |
| *Tregs* | 0.26 (0.15-0.37) | 0.22 (0.15-0.40) | 0.27 (0.18-0.40) | 0.888 |
| *NK T cells* | 0.12 (0.06-0.38) | 0.20 (0.08-0.46) | 0.18 (0.07-0.59) | 0.449 |
| *NK cells* | 0.46 (0.21-0.87) | 0.51 (0.29-0.74) | 0.42 (0.19-0.73) | 0.577 |
| *Monocytes* | 5.59 (4.70-7.81) | 6.16 (4.52-8.56) | 5.58 (4.26-6.98) | 0.655 |
| *Myeloid (classical) DC* | 0.02 (0.01-0.04) | 0.03 (0.01-0.04) | 0.02 (0.01-0.04) | 0.940 |
| *Plasmacytoid DC* | 0.03 (0.02-0.04) | 0.03 (0.02-0.06) | 0.03 (0.03-0.05) | 0.592 |
| *Basophils* | 0.14 (0.08-0.21) | 0.12 (0.08-0.19) | 0.14 (0.08-0.23) | 0.858 |
| *Neutrophils* | 82.2 (76.1-85.4) | 82.5 (74.0-86.1) | 78.9 (75.5-85.4) | 0.934 |
| *Eosinophils* | 0.90 (0.54-2.53) | 0.81 (0.40-1.88) | 0.96 (0.35-2.42) | 0.650 |

| **Subset** | **Table 4B** | **T0 (Median (IQR))** |  |  |
| --- | --- | --- | --- | --- |
|  | *Placebo* | *2 gram TXA* | *4 gram TXA* | *p-value* |
| *B cells* | 155491 (99211 - 230289) | 136697 (80837 - 189488) | 159602 (86023 - 234028) | 0.377 |
| *CD4^+^ T cells* | 514512 (345261 - 660623) | 407650 (304199 - 513876) | 470054 (360472 - 663742) | 0.065 |
| *CD8^+^ T cells* | 308864 (190131 - 483935) | 241360 (163202 - 394438) | 297005 (178295 - 471854) | 0.535 |
| *Tregs* | 28398 (18908 - 46386) | 28602 (15412 - 38422) | 26995 (18998 - 34305) | 0.819 |
| *NK T cells* | 43322 (9976 - 184643) | 44781 (26506 - 104801) | 69296 (24789 - 137021) | 0.696 |
| *NK cells* | 192902 (104584 - 320654) | 209431 (121872 - 325805) | 168139 (84273 - 342413) | 0.829 |
| *Monocytes* | 329382 (212880 - 502844) | 296724 (127847 - 418892) | 279569 (164490 - 381940) | 0.201 |
| *Myeloid (classical) DC* | 3633 (2331 - 7687) | 3510 (1963 - 4845) | 3305 (1732 - 5832) | 0.331 |
| *Plasmacytoid DC* | 7728 (3772 - 10581) | 6733 (4337 - 8738) | 6880 (3991 - 11960) | 0.537 |
| *Basophils* | 12117 (8779 - 21257) | 13088 (6649 - 21253) | 13531 (7578 - 18901) | 0.779 |
| *Neutrophils* | 5555519 (3288728 - 8827508) | 4402878 (2090844 - 7689032) | 5996580 (3300409 - 8357401) | 0.240 |
| *Eosinophils* | 69310 (46298 - 107886) | 60063 (37718 - 91667) | 66874 (39311 - 126741) | 0.426 |
|  |  | **T6 (Median (IQR))** |  |  |
| *B cells* | 74559 (49356 - 111365) | 85724 (48826 - 170570) | 88881 (59293 - 120660) | 0.271 |
| *CD4^+^ T cells* | 169421 (98576 - 311671) | 173748 (114149 - 306136) | 180483 (107139 - 337795) | 0.856 |
| *CD8^+^ T cells* | 93943 (52106 - 185417) | 99405 (64174 - 191534) | 105205 (53611 - 181492) | 0.658 |
| *Tregs* | 11674 (6476 - 24621) | 11572 (6618 - 22750) | 11675 (6943 - 18799) | 0.978 |
| *NK T cells* | 6660 (1486 - 21981) | 9135 (4082 - 31429) | 8417 (4079 - 25883) | 0.234 |
| *NK cells* | 27737 (21924 - 44883) | 38481 (26226 - 68773) | 34312 (19334 - 65462) | 0.346 |
| *Monocytes* | 387016 (236598 - 544262) | 490621 (231735 - 715565) | 475204 (347308 - 763971) | 0.178 |
| *Myeloid (classical) DC* | 3924 (2412 - 6365) | 3727 (2202 - 7311) | 4336 (2580 - 9051) | 0.592 |
| *Plasmacytoid DC* | 1938 (732.2 - 3832) | 2190 (1022 - 4220) | 1760 (1077 - 3330) | 0.800 |
| *Basophils* | 2892 (1703 - 5324) | 4653 (1745 - 10102) | 3448 (1798 - 7057) | 0.493 |
| *Neutrophils* | 6478407 (4130654 - 7982005) | 7506838 (4243688 - 9613146) | 6798336 (4938644 - 9819886) | 0.246 |
| *Eosinophils* | 14283 (6447 - 27708) | 23121 (9270 - 29201) | 12804 (7460 - 31878) | 0.265 |
|  |  | **T24 (Median (IQR))** |  |  |
| *B cells* | 101476 (84122 - 178928) | 137733 (97298 - 188703) | 143649 (110028 - 229279) | ***0.013*** |
| *CD4^+^ T cells* | 244637 (191130 - 305410) | 244827 (198010 - 330289) | 401040 (242500 - 491995) | ***0.001*** |
| *CD8^+^ T cells* | 124971 (90950 - 173846) | 155010 (99487 - 219260) | 213990 (104831 - 262992) | ***0.011*** |
| *Tregs* | 18129 (11386 - 21306) | 18797 (12068 - 28056) | 29055 (16235 - 34221) | ***0.007*** |
| *NK T cells* | 13251 (2791 - 24532) | 13079 (6363 - 39958) | 19275 (6393 - 34836) | 0.132 |
| *NK cells* | 30650 (23578 - 51791) | 37557 (23421 - 63733) | 36317 (20373 - 66985) | 0.786 |
| *Monocytes* | 459845 (286029 - 596464) | 463420 (317238 - 704070) | 438580 (331692 - 598346) | 0.682 |
| *Myeloid (classical) DC* | 5446 (3157 - 7490) | 5122 (2319 - 7265) | 5355 (2773 - 9994) | 0.638 |
| *Plasmacytoid DC* | 1980 (1044 - 2793) | 2163 (1265 - 3258) | 2687 (1517 - 3948) | 0.112 |
| *Basophils* | 8955 (4631 - 13768) | 7340 (3172 - 13429) | 7854 (4678 - 13596) | 0.792 |
| *Neutrophils* | 5995468 (3844604 - 7242316) | 5942920 (4197549 - 9487819) | 5898534 (4613837 - 7775440) | 0.770 |
| *Eosinophils* | 22311 (10573 - 39389) | 28474 (15841 - 40681) | 22974 (10823 - 71902) | 0.588 |
|  |  | **T72 (Median (IQR))** |  |  |
| *B cells* | 57095 (32431 - 106379) | 93763 (54815 - 141967) | 82538 (56629 - 126853) | 0.076 |
| *CD4^+^ T cells* | 140155 (128575 - 255857) | 153205 (124906 - 375687) | 244802 (137372 - 363346) | 0.062 |
| *CD8^+^ T cells* | 73562 (47541 - 105075) | 90675 (41607 - 208585) | 107133 (45274 - 164659) | 0.094 |
| *Tregs* | 12883 (6281 - 19697) | 14761 (7654 - 31048) | 19633 (13094 - 26695) | 0.099 |
| *NK T cells* | 7774 (3250 - 15072) | 10880 (4658 - 19179) | 15287 (3752 - 36495) | 0.105 |
| *NK cells* | 17510 (10795 - 39689) | 32171 (11521 - 48752) | 30336 (13147 - 48601) | 0.384 |
| *Monocytes* | 276297 (169077 - 427655) | 348939 (266019 - 520132) | 356425 (250513 - 553656) | 0.178 |
| *Myeloid (classical) DC* | 1018 (592.2 - 2060) | 1257 (381.6 - 3296) | 1147 (925.5 - 2550) | 0.690 |
| *Plasmacytoid DC* | 1417 (738.9 - 2428) | 1770 (857.3 - 2811) | 2267 (1548 - 2810) | 0.056 |
| *Basophils* | 6782 (3096 - 14279) | 7164 (4366 - 12295) | 8368 (5082 - 16364) | 0.327 |
| *Neutrophils* | 3850136 (2641309 - 6682313) | 5481548 (2559977 - 6832421) | 5092148 (4050487 - 7601703) | 0.145 |
| *Eosinophils* | 49579 (22065 - 133066) | 49298 (25018 - 103801) | 62169 (27264 - 134127) | 0.705 |

**Supplementary Table 5. Soluble inflammatory mediator levels and complement activation in peripheral blood in each study group over time.** Data from all patients with available samples, results at a given time point (T0, T6, T24, T72) between treatment groups (placebo, 2 gram TXA, 4 gram TXA). For analytes cytokines and chemokines, units are in pg/mL. For CH50, units are as U Eq/mL. Concentrations of each analyte are reported, with median (Q1-Q3) listed. Kruskal-Wallis tests were performed for each analyte at the given time point across treatment groups; p < 0.05 was considered significant, and bolded and italicized.

| **Analyte** |  | **T0 (Median (IQR))** |  |  |
| --- | --- | --- | --- | --- |
|  | *Placebo* | *2 gram TXA* | *4 gram TXA* | *p-value* |
| *ITAC* | 29.34 (16.87-44.73) | 34.24 (24.36-56.26) | 32.75 (21.19-49.80) | 0.334 |
| *GM-CSF* | 64.69 (39.15-98.04) | 67.79 (44.85-112.00) | 67.58 (43.84-128.00) | 0.841 |
| *Fractalkine* | 136.90 (75.83-229.90) | 223.60 (93.70-336.50) | 146.20 (102.40-211.60) | 0.215 |
| *IFNgamma* | 17.47 (9.30-27.32) | 23.46 (13.14-38.28) | 17.59 (13.43-39.21) | 0.147 |
| *IL-10* | 105.10 (38.99-257.50) | 116.00 (41.68-241.80) | 84.39 (45.30-149.30) | 0.561 |
| *MIP-3a* | 22.95 (15.28-31.96) | 22.95 (13.79-29.49) | 21.94 (17.39-28.07) | 0.991 |
| *IL-12p70* | 9.12 (4.63-14.38) | 9.50 (6.64-17.93) | 8.64 (5.96-12.82) | 0.480 |
| *IL-13* | 9.08 (5.25-16.70) | 11.80 (5.60-28.81) | 11.11 (5.28-26.29) | 0.291 |
| *IL-17A* | 18.73 (10.83-29.56) | 21.51 (9.88-30.47) | 15.08 (9.89-24.99) | 0.339 |
| *IL-1beta* | 3.90 (2.74-5.81) | 4.17 (2.32-7.36) | 3.75 (2.59-6.20) | 0.842 |
| *IL-2* | 4.01 (2.90-5.66) | 4.17 (2.71-7.65) | 4.29 (2.60-7.63) | 0.659 |
| *IL-21* | 7.02 (3.02-11.47) | 6.28 (3.48-12.70) | 6.13 (3.47-9.91) | 0.885 |
| *IL-4* | 58.16 (37.44-98.26) | 69.45 (45.12-96.88) | 58.24 (41.15-80.14) | 0.726 |
| *IL-23* | 406.50 (242.10-779.20) | 724.50 (435.40-1,142.00) | 428.30 (234.60-879.60) | ***0.024*** |
| *IL-5* | 9.06 (5.59-13.47) | 9.30 (3.97-15.82) | 9.48 (5.10-15.96) | 0.824 |
| *IL-6* | 63.56 (26.61-117.90) | 54.48 (22.18-194.00) | 39.70 (18.07-101.50) | 0.284 |
| *IL-7* | 16.52 (9.86-23.83) | 18.53 (12.44-31.42) | 15.33 (11.31-24.99) | 0.427 |
| *IL-8* | 13.78 (7.86-27.74) | 15.25 (10.20-32.57) | 13.59 (9.61-24.89) | 0.773 |
| *MIP-1alpha* | 27.85 (21.63-42.26) | 32.57 (22.52-42.59) | 30.98 (20.04-43.32) | 0.813 |
| *MIP-1beta* | 35.44 (24.36-50.61) | 44.91 (30.29-54.74) | 37.66 (31.06-49.42) | 0.174 |
| *TNFa* | 16.40 (12.10-21.22) | 19.16 (13.23-23.71) | 16.40 (13.25-21.13) | 0.256 |
| *CH50* | 80.04 (53.71-105.70) | 70.32 (49.96-98.81) | 88.42 (56.30-116.90) | 0.277 |
|  |  | **T6 (Median (IQR))** |  |  |
| *ITAC* | 23.83 (17.01-35.16) | 28.24 (21.27-48.70) | 34.16 (27.14-43.55) | ***0.026*** |
| *GM-CSF* | 78.89 (44.47-97.39) | 69.91 (57.06-133.30) | 96.97 (55.42-147.40) | 0.195 |
| *Fractalkine* | 157.70 (106.90-240.80) | 222.40 (121.10-347.00) | 174.50 (133.60-256.80) | 0.201 |
| *IFNgamma* | 19.59 (12.79-29.22) | 26.39 (18.19-41.48) | 31.39 (19.19-40.64) | ***0.012*** |
| *IL-10* | 73.61 (40.30-147.80) | 84.08 (44.97-132.20) | 80.28 (49.72-136.50) | 0.755 |
| *MIP-3a* | 36.79 (24.79-58.04) | 34.23 (21.56-81.47) | 37.33 (22.54-67.65) | 0.881 |
| *IL-12p70* | 8.47 (4.56-14.93) | 11.87 (7.70-16.40) | 12.11 (7.70-14.19) | 0.086 |
| *IL-13* | 13.19 (5.98-18.16) | 15.29 (7.80-27.08) | 18.96 (7.92-26.84) | 0.106 |
| *IL-17A* | 15.29 (10.43-24.90) | 23.12 (12.03-31.71) | 22.54 (12.91-32.41) | 0.110 |
| *IL-1beta* | 4.64 (2.91-7.04) | 5.46 (2.88-7.89) | 6.17 (3.66-9.05) | 0.296 |
| *IL-2* | 4.32 (3.25-5.71) | 5.53 (3.14-8.11) | 5.92 (3.11-8.50) | ***0.044*** |
| *IL-21* | 8.36 (4.77-12.26) | 9.32 (5.27-15.49) | 9.88 (4.01-14.99) | 0.582 |
| *IL-4* | 57.38 (38.70-76.35) | 66.39 (46.32-89.06) | 73.09 (46.44-95.19) | 0.201 |
| *IL-23* | 443.20 (276.40-691.90) | 714.70 (442.80-1,011.00) | 534.60 (319.60-1,057.00) | ***0.024*** |
| *IL-5* | 9.18 (5.37-13.06) | 10.11 (7.19-16.79) | 12.36 (6.67-16.90) | 0.099 |
| *IL-6* | 252.20 (142.20-1,365.00) | 223.10 (67.56-771.00) | 160.30 (69.61-526.10) | 0.151 |
| *IL-7* | 15.34 (12.44-25.06) | 19.46 (13.61-30.57) | 23.06 (14.61-26.89) | 0.153 |
| *IL-8* | 69.31 (25.12-124.70) | 39.21 (19.84-115.30) | 26.64 (17.46-73.42) | 0.071 |
| *MIP-1alpha* | 25.01 (20.09-38.27) | 35.24 (23.04-47.94) | 29.06 (22.75-43.65) | ***0.041*** |
| *MIP-1beta* | 34.89 (26.89-50.46) | 43.92 (31.40-53.54) | 40.50 (31.58-54.38) | 0.120 |
| *TNFa* | 15.27 (10.85-19.76) | 17.54 (14.04-22.56) | 18.77 (13.13-24.65) | ***0.025*** |
| *CH50* | 65.53 (47.15-89.31) | 73.61 (49.64-97.93) | 69.59 (50.73-95.91) | 0.787 |
|  |  | **T24 (Median (IQR))** |  |  |
| *ITAC* | 20.99 (13.70-26.14) | 20.76 (14.38-31.59) | 22.05 (14.60-29.72) | 0.679 |
| *GM-CSF* | 69.44 (46.11-93.13) | 59.71 (40.80-117.10) | 77.64 (45.62-122.00) | 0.642 |
| *Fractalkine* | 129.40 (96.47-222.10) | 204.00 (110.80-281.40) | 133.20 (111.30-188.50) | 0.225 |
| *IFNgamma* | 19.10 (9.89-27.13) | 20.56 (11.54-38.28) | 21.96 (12.16-32.09) | 0.307 |
| *IL-10* | 55.56 (41.18-81.44) | 53.83 (30.69-101.80) | 64.41 (27.63-131.40) | 0.784 |
| *MIP-3a* | 32.82 (21.68-50.13) | 33.76 (18.83-64.45) | 36.93 (24.11-76.61) | 0.523 |
| *IL-12p70* | 8.22 (4.93-16.74) | 8.71 (4.35-17.19) | 8.98 (6.42-14.50) | 0.874 |
| *IL-13* | 12.98 (5.47-18.62) | 10.96 (8.06-25.65) | 13.97 (5.25-20.77) | 0.647 |
| *IL-17A* | 13.02 (8.84-22.81) | 18.18 (10.77-33.54) | 17.37 (11.01-28.10) | 0.241 |
| *IL-1beta* | 4.12 (2.66-6.11) | 4.07 (2.14-6.72) | 4.61 (3.33-6.57) | 0.791 |
| *IL-2* | 4.20 (2.37-6.11) | 4.97 (2.65-7.50) | 4.63 (3.68-6.58) | 0.321 |
| *IL-21* | 7.81 (4.46-12.36) | 6.53 (4.50-12.15) | 7.72 (4.49-12.64) | 0.906 |
| *IL-4* | 44.26 (33.52-69.62) | 47.21 (31.47-82.97) | 52.64 (33.90-74.66) | 0.813 |
| *IL-23* | 366.40 (297.80-881.20) | 618.90 (354.10-1,106.00) | 438.60 (238.20-871.20) | 0.235 |
| *IL-5* | 9.14 (5.89-13.15) | 7.88 (4.21-16.72) | 10.70 (5.19-14.88) | 0.797 |
| *IL-6* | 206.00 (95.09-466.10) | 211.10 (80.02-826.30) | 247.00 (84.61-975.20) | 0.962 |
| *IL-7* | 16.13 (12.73-24.74) | 18.50 (11.22-33.11) | 19.85 (11.69-24.09) | 0.865 |
| *IL-8* | 33.99 (21.00-54.96) | 38.01 (20.33-62.20) | 37.85 (17.73-61.05) | 0.972 |
| *MIP-1alpha* | 24.48 (19.14-40.10) | 33.90 (20.45-47.71) | 25.66 (20.27-41.38) | 0.315 |
| *MIP-1beta* | 29.40 (23.52-37.02) | 32.56 (23.64-49.71) | 33.64 (22.88-42.98) | 0.288 |
| *TNFa* | 15.74 (12.16-19.32) | 17.23 (12.05-23.91) | 16.95 (13.09-24.28) | 0.385 |
| *CH50* | 76.89 (50.52-99.14) | 71.66 (46.69-117.20) | 73.33 (51.79-103.70) | 0.991 |
|  |  | **T72 (Median (IQR))** |  |  |
| *ITAC* | 21.69 (13.95-33.39) | 24.62 (16.44-42.11) | 21.69 (13.88-35.03) | 0.380 |
| *GM-CSF* | 64.77 (46.02-99.57) | 60.91 (34.62-106.40) | 52.88 (36.17-95.40) | 0.795 |
| *Fractalkine* | 100.80 (78.42-189.50) | 169.40 (109.30-251.30) | 124.20 (82.34-188.40) | 0.065 |
| *IFNgamma* | 13.02 (9.04-28.52) | 17.78 (11.48-31.35) | 14.97 (10.58-30.12) | 0.305 |
| *IL-10* | 35.00 (18.52-48.59) | 29.44 (14.25-44.60) | 42.39 (16.31-66.45) | 0.225 |
| *MIP-3a* | 31.96 (18.68-51.47) | 37.10 (21.34-122.20) | 27.61 (21.78-55.98) | 0.313 |
| *IL-12p70* | 7.58 (3.78-17.51) | 9.67 (5.26-16.35) | 9.45 (5.60-12.40) | 0.525 |
| *IL-13* | 10.07 (4.39-18.97) | 10.89 (5.41-27.35) | 10.27 (5.01-22.81) | 0.484 |
| *IL-17A* | 14.63 (8.85-30.35) | 19.67 (10.74-40.52) | 16.67 (11.01-28.27) | 0.495 |
| *IL-1beta* | 3.14 (2.39-6.14) | 3.94 (2.72-5.44) | 3.44 (2.17-4.79) | 0.682 |
| *IL-2* | 4.12 (2.74-7.07) | 4.26 (2.10-8.88) | 3.49 (2.19-6.05) | 0.522 |
| *IL-21* | 5.51 (2.72-10.92) | 6.44 (3.44-16.46) | 6.95 (3.54-9.70) | 0.440 |
| *IL-4* | 34.93 (23.13-69.19) | 49.01 (27.54-74.40) | 40.50 (23.98-75.91) | 0.433 |
| *IL-23* | 404.20 (252.20-976.40) | 580.30 (336.50-952.00) | 351.50 (240.30-813.10) | 0.449 |
| *IL-5* | 8.58 (7.07-13.48) | 8.67 (5.47-17.32) | 8.05 (6.06-16.47) | 0.922 |
| *IL-6* | 43.66 (23.36-71.15) | 72.78 (27.93-172.70) | 67.95 (32.51-156.60) | ***0.017*** |
| *IL-7* | 16.86 (11.11-19.88) | 18.39 (10.10-28.04) | 16.24 (10.52-22.46) | 0.802 |
| *IL-8* | 18.44 (12.62-23.98) | 26.50 (16.85-41.48) | 23.05 (16.79-39.78) | ***0.011*** |
| *MIP-1alpha* | 25.64 (17.38-44.00) | 28.50 (18.58-44.47) | 27.24 (17.80-40.31) | 0.873 |
| *MIP-1beta* | 28.16 (22.41-36.24) | 35.48 (23.80-45.61) | 29.02 (22.94-41.31) | 0.120 |
| *TNFa* | 16.26 (12.70-21.83) | 19.38 (15.40-25.71) | 20.09 (15.32-27.10) | 0.054 |
| *CH50* | 143.60 (100.80-183.30) | 144.50 (97.61-169.50) | 137.00 (116.60-184.60) | 0.974 |

**Supplementary Table 6. Hemostatic and endothelial parameters measured in each study group over time.** Data from all patients with available samples, results at a given time point (T0, T6, T24, T72) between treatment groups (placebo, 2 gram TXA, 4 gram TXA). Values for each parameter are reported, with median (IQR) listed. Kruskal-Wallis tests were performed for each parameter at the given time point across treatment groups; p < 0.05 was considered significant, and bolded and italicized. Abbreviations: KNG1, Kininogen; PF1.2, prothrombin fragment 1 and 2; TAT, thrombin/anti-thrombin complex; FIB, fibrinogen; PAP, plasmin/alpha-2-antiplasmin complex; tPA, tissue plasminogen activator; D-di, D-dimer; vWF, von Willebrand factor; SDC1, syndecan-1; R-Time, reaction time; K-time, kinetics; α, alpha angle; MA, maximal amplitude; LY30, % lysis at 30 minutes.

| **Parameter** |  | **T0 (Median (IQR))** |  |  |
| --- | --- | --- | --- | --- |
|  | *Placebo* | *2 gram TXA* | *4 gram TXA* | *p-value* |
| *KNG1 (pg/mL)* | 93.00 (61.00-257.0) | 112.5 (58.00-261.0) | 114.0 (62.00-434.8) | 0.699 |
| *PF1.2 (pg/mL)* | 3986 (2694-5838) | 3087 (2677-4266) | 4158 (2367-5615) | 0.203 |
| *TAT (pg/mL)* | 126.0 (54.00-236.0) | 131.0 (64.00-305.0) | 160.5 (55.25-291.5) | 0.787 |
| *FIB (mg/dL)* | 179.5 (143.3-251.0) | 190.0 (143.0-221.8) | 182.0 (142.1-244.6) | 0.860 |
| *PAP (pg/mL)* | 2672 (1337-5522) | 2597 (962-5949) | 3695 (999.5-8325) | 0.718 |
| *tPA (pg/mL)* | 1763 (1116-3310) | 2053 (937.8-3985) | 2252 (966-3510) | 0.933 |
| *D-di (µg/mL)* | 4.830 (2.140-10.63) | 3.150 (2.000-6.180) | 3.700 (2.130-10.64) | 0.624 |
| *vWF (%)* | 187.0 (148.2-285.0) | 178.0 (131.0-254.5) | 196.0 (146.4-246.1) | 0.594 |
| *SDC1 (pg/mL)* | 1903 (1388-2708) | 2180 (1273-4096) | 2214 (1365-3447) | 0.808 |
| *R time (min)* | 6.000 (5.050-6.800) | 6.200 (5.700-7.100) | 6.200 (5.200-7.250) | 0.448 |
| *K time (min)* | 60.00 (2.250-60.00) | 60.00 (3.130-60.00) | 60.00 (2.800-60.00) | 0.872 |
| *α (°)* | 66.30 (61.43-72.15) | 67.20 (60.53-70.25) | 64.55 (47.18-71.28) | 0.489 |
| *MA (mm)* | 18.15 (14.63-25.43) | 19.00 (12.63-24.28) | 18.75 (12.08-23.38) | 0.764 |
| *LY30 (%)* | 0.00 (0.00-0.00) | 0.00 (0.00-0.00) | 0.00 (0.00-0.00) | >0.999 |
|  |  | **T6 (Median (IQR))** |  |  |
| *KNG1 (pg/mL)* | 103.0 (54.00-323.0) | 122.0 (62.75-369.5) | 133.0 (63.50-444.0) | 0.793 |
| *PF1.2 (pg/mL)* | 4377 (2691-5979) | 3941 (2969-5006) | 4632 (2918-5699) | 0.771 |
| *TAT (pg/mL)* | 74.00 (40.00-150.0) | 83.50 (41.50-163.5) | 94.00 (50.50-171.0) | 0.431 |
| *FIB (mg/dL)* | 215.5 (163.3-275.8) | 213.6 (186.0-245.0) | 216.0 (164.8-276.3) | 0.883 |
| *PAP (pg/mL)* | 2080 (1337-4312) | 2767 (1092-7645) | 3273 (1579-7329) | 0.227 |
| *tPA (pg/mL)* | 1210 (891.0-2014) | 1702 (693.8-2612) | 1320 (818.5-2086) | 0.831 |
| *D-di (µg/mL)* | 8.410 (4.690-11.60) | 3.840 (2.300-10.69) | 3.170 (2.510-11.46) | ***0.003*** |
| *vWF (%)* | 236.0 (188.0-288.1) | 234.0 (181.5-332.6) | 224.0 (180.2-320.7) | 0.811 |
| *SDC1 (pg/mL)* | 2582 (1876-4146) | 2377 (1616-5305) | 3394 (2016-4583) | 0.440 |
| *R time (min)* | 6.650 (5.050-7.200) | 7.100 (5.800-7.600) | 6.800 (5.400-7.950) | 0.267 |
| *K time (min)* | 60.00 (2.500-60.00) | 60.00 (2.450-60.00) | 60.00 (1.800-60.00) | 0.875 |
| *α (°)* | 64.50 (59.90-69.40) | 65.50 (60.05-70.30) | 67.50 (58.40-71.30) | 0.930 |
| *MA (mm)* | 19.90 (15.60-24.30) | 19.30 (14.55-24.55) | 19.90 (17.20-25.60) | 0.678 |
| *LY30 (%)* | 0.00 (0.00-0.00) | 0.00 (0.00-0.00) | 0.00 (0.00-0.00) | >0.999 |
|  |  | **T24 (Median (IQR))** |  |  |
| *KNG1 (pg/mL)* | 85.00 (50.50-228.5) | 109.0 (61.00-372.5) | 108.0 (62.75-384.3) | 0.545 |
| *PF1.2 (pg/mL)* | 3761 (2781-5831) | 3979 (2568-5745) | 3900 (2239-5461) | 0.863 |
| *TAT (pg/mL)* | 38.00 (23.00-59.50) | 37.00 (20.00-67.50) | 50.00 (28.75-77.50) | 0.298 |
| *FIB (mg/dL)* | 452.0 (363.1-492.8) | 391.0 (332.0-475.0) | 375.0 (313.1-464.5) | 0.081 |
| *PAP (pg/mL)* | 1356 (687.3-1909) | 1419 (560.0-2570) | 1700 (727.5-2646) | 0.528 |
| *tPA (pg/mL)* | 923.0 (644.0-2814) | 1166 (520.5-1966) | 844.5 (516.3-1563) | 0.228 |
| *D-di (µg/mL)* | 4.920 (3.460-11.85) | 3.250 (2.100-8.320) | 3.740 (1.950-8.020) | ***0.006*** |
| *vWF (%)* | 242.7 (193.0-314.0) | 246.0 (171.0-306.0) | 229.0 (178.0-296.9) | 0.876 |
| *SDC1 (pg/mL)* | 2211 (1620-2874) | 2526 (1325-3971) | 2724 (1826-4021) | 0.228 |
| *R time (min)* | 7.400 (6.480-8.330) | 8.200 (6.700-9.500) | 7.400 (6.080-9.830) | 0.434 |
| *K time (min)* | 1.400 (1.100-1.850) | 1.600 (1.200-4.350) | 1.850 (1.400-3.630) | 0.100 |
| *α (°)* | 70.70 (65.60-73.60) | 69.30 (61.45-73.60) | 67.55 (59.98-69.38) | 0.061 |
| *MA (mm)* | 31.50 (27.10-35.95) | 30.70 (23.50-34.15) | 29.45 (23.78-32.80) | 0.264 |
| *LY30 (%)* | 0.00 (0.00-0.00) | 0.00 (0.00-0.00) | 0.00 (0.00-0.00) | >0.999 |
|  |  | **T72 (Median (IQR))** |  |  |
| *KNG1 (pg/mL)* | 106.0 (72.00-340.0) | 136.0 (88.00-420.8) | 193.0 (77.00-476.3) | 0.593 |
| *PF1.2 (pg/mL)* | 5149 (3208-7299) | 4406 (2883-6649) | 4654 (3182-6916) | 0.676 |
| *TAT (pg/mL)* | 25.00 (15.50-42.00) | 27.50 (15.25-40.50) | 30.50 (20.50-40.75) | 0.535 |
| *FIB (mg/dL)* | 794.5 (613.8-909.5) | 691.9 (586.0-830.5) | 711.7 (626.8-853.1) | 0.321 |
| *PAP (pg/mL)* | 1675 (1258-2232) | 1954 (1286-2794) | 1921 (1325-3175) | 0.488 |
| *tPA (pg/mL)* | 874.0 (433.0-2043) | 856.5 (469.5-1768) | 766.0 (476.8-1200) | 0.865 |
| *D-di (µg/mL)* | 5.130 (3.850-7.900) | 4.740 (3.070-6.500) | 4.630 (3.470-8.350) | 0.681 |
| *vWF (%)* | 282.3 (241.9-341.0) | 324.0 (221.7-371.1) | 308.0 (246.8-394.5) | 0.771 |
| *SDC1 (pg/mL)* | 2817 (1708-3703) | 2691 (1702-3969) | 2877 (2015-4930) | 0.533 |
| *R time (min)* | 8.900 (7.150-11.70) | 8.950 (7.430-10.75) | 8.800 (7.150-10.53) | 0.958 |
| *K time (min)* | 1.400 (1.000-1.750) | 1.500 (1.200-1.800) | 1.300 (1.100-1.650) | 0.298 |
| *α (°)* | 69.70 (61.90-74.70) | 67.70 (62.03-71.83) | 71.50 (65.85-72.90) | 0.254 |
| *MA (mm)* | 46.70 (39.00-50.95) | 43.00 (38.00-48.38) | 44.45 (40.10-51.55) | 0.187 |
| *LY30 (%)* | 0.00 (0.00-0.00) | 0.00 (0.00-0.00) | 0.00 (0.00-0.00) | >0.999 |

**Supplementary Table 7. Variables used in correlation matrices.**

| **Variable** | **Definition** |
| --- | --- |
| itac_conc | ITAC (pg/mL) |
| gmcsf_conc | GM-CSF (pg/mL) |
| frac_conc | Fractalkine (pg/mL) |
| ifng_conc | IFNg (pg/mL) |
| il10_conc | IL-10 (pg/mL) |
| mip3a_conc | MIP-3a (pg/mL) |
| il12p70_conc | IL-12p70 (pg/mL) |
| il13_conc | IL-13 (pg/mL) |
| il17a_conc | IL-17A (pg/mL) |
| il1b_conc | IL-1beta (pg/mL) |
| il2_conc | IL-2 (pg/mL) |
| il21_conc | IL-21 (pg/mL) |
| il4_conc | IL-4 (pg/mL) |
| il23_conc | IL-23 (pg/mL) |
| il5_conc | IL-5 (pg/mL) |
| il6_conc | IL-6 (pg/mL) |
| il7_conc | IL-7 (pg/mL) |
| il8_conc | IL-8 (pg/mL) |
| mip1a_conc | MIP-1alpha (pg/mL) |
| mip1b_conc | MIP-1beta (pg/mL) |
| tnfa_conc | TNF-alpha (pg/mL) |
| vwf_percent | von Willebrand factor (vWF, %) |
| fib_conc | Fibrinogen (FIB, mg/dL) |
| ddi_conc | D-dimer (D-Di, ug/mL) |
| pai1_conc | Plasminogen activator inhibitor 1 (PAI-1, U/mL) |
| ch50_percent | Complement Activity (CH50, %) |
| kng1_conc | Kininogen-1 precursor (KNG-1, pg/mL) |
| pap_conc | Plasmin-antiplasmin Complex (PAP, pg/mL) |
| pf12_conc | Prothrombin fragment 1 and 2 (PF12, pg/mL) |
| syndecan1_conc | Syndecan-1 (SDC1, pg/mL) |
| tat_conc | Thrombin anti-thrombin complex (TAT, pg/mL) |
| tpa_conc | Tissue plasminogen activator (tPA, pg/mL) |
| total_pbl_number | Number of Total Peripheral Blood Leukocytes (PBL) |
| tregs_freq | Frequency of Tregs Among CD4+ T cells |
| cd69_cd4tcells_freq | Frequency of CD69+ CD4+ T cells Among CD4+ T cells |
| pd1_cd4tcells_freq | Frequency of PD-1+CD4+ T cells Among CD4+ T cells |
| hladr_cd4tcells_freq | Frequency of HLA-DR+CD4+ T cells Among CD4+ T cells |
| cd69_cd8tcells_freq | Frequency of CD69+ CD8+ T cells Among CD8+ T cells |
| pd1_cd8tcells_freq | Frequency of PD-1+CD8+ T cells Among CD8+ T cells |
| hladr_cd8tcells_freq | Frequency of HLA-DR+CD8+ T cells Among CD8+ T cells |
| hladr_nkt_freq | Frequency of HLA-DR+ NKT cells Among NKT cells |
| cd56dim_nk_freq | Frequency of CD56dim cells Among NK cells |
| cd56bright_nk_freq | Frequency of CD56bright cells Among NK cells |
| hladr_cd56dim_freq | Frequency of HLA-DR Among CD56dim cells |
| cd8_cd56dim_freq | Frequency of CD8+ Among CD56dim cells |
| hladr_cd56bright_freq | Frequency of HLA-DR Among CD56bright cells |
| cd8_cd56bright_freq | Frequency of CD8+ Among CD56bright cells |
| neut_pbl_freq | Frequency of Neutrophils Among PBL |
| lymph_pbl_freq | Frequency of Lymphocytes Among PBL |
| hladr_neut_freq | Frequency of HLA-DR+ Neutrophils Among Neutrophils |
| bcells_pbl_freq | Frequency of B cells Among PBL |
| cd4tcells_pbl_freq | Frequency of CD4 T cells Among PBL |
| cd8tcells_pbl_freq | Frequency of CD8 T cells Among PBL |
| nkt_pbl_freq | Frequency of NKT cells Among PBL |
| tregs_pbl_freq | Frequency of Tregs Among PBL |
| nk_pbl_freq | Frequency of NK cells Among PBL |
| classicmonos_pbl_freq | Frequency of Classical Monocytes Among PBL |
| nonclassicmonos_pbl_freq | Frequency of Nonclassical Monocytes Among PBL |
| cdc_pbl_freq | Frequency of conventional dendritic cells (cDC) Among PBL |
| pdc_pbl_freq | Frequency of plasmacytoid dendritic cells (pDC) Among PBL |
| baso_pbl_freq | Frequency of Basophils Among PBL |
| neut_pbl_freq_2 | Frequency of Neutrophils Among PBL |
| eos_pbl_freq | Frequency of Eosinophils Among PBL |
| hladr_bcells_mfi | HLA-DR MFI on B cells |
| hladr_classicmono_mfi | HLA-DR MFI on Classical Monocytes |
| cd86_classicmono_mfi | CD86 MFI on Classical Monocytes |
| hladr_nonclassicmono_mfi | HLA-DR MFI on Nonclassical Monocytes |
| cd86_nonclassicmono_mfi | CD86 MFI on Nonclassical Monocytes |
| hladr_cdc_mfi | HLA-DR MFI on cDC |
| cd86_cdc_mfi | CD86 MFI on cDC |
| hladr_pdc_mfi | HLA-DR MFI on pDC |
| cd86_pdc_mfi | CD86 MFI on pDC |
| cd16_classicmono_mfi | CD16 MFI on Classical Monocytes |
| cd16_nonclassicmono_mfi | CD16 MFI on Nonclassical Monocytes |
| cd11c_neut_mfi | CD11c MFI on Neutrophils |
| cd11b_neut_mfi | CD11b MFI on Neutrophils |
| cd66b_neut_mfi | CD66b MFI on Neutrophils |
| cd16_neut_mfi | CD16 MFI on Neutrophils |
| cd62l_neut_mfi | CD62L MFI on Neutrophils |
| mean_fsc_neut | Mean FSC on Neutrophils |
| mean_ssc_neut | Mean SSC on Neutrophils |

**Supplementay Table 8. Variables used in principal component analysis.**

| **Variable** | **Definition** |
| --- | --- |
| subj_num | Subject Number |
| timepoint_event_type | Time Point |
| txa_group | TXA dosing group |
| itac_conc | ITAC (pg/mL) |
| gmcsf_conc | GM-CSF (pg/mL) |
| frac_conc | Fractalkine (pg/mL) |
| ifng_conc | IFNg (pg/mL) |
| il10_conc | IL-10 (pg/mL) |
| mip3a_conc | MIP-3a (pg/mL) |
| il12p70_conc | IL-12p70 (pg/mL) |
| il13_conc | IL-13 (pg/mL) |
| il17a_conc | IL-17A (pg/mL) |
| il4_conc | IL-4 (pg/mL) |
| il23_conc | IL-23 (pg/mL) |
| il5_conc | IL-5 (pg/mL) |
| il6_conc | IL-6 (pg/mL) |
| il8_conc | IL-8 (pg/mL) |
| mip1a_conc | MIP-1alpha (pg/mL) |
| mip1b_conc | MIP-1beta (pg/mL) |
| tnfa_conc | TNF-alpha (pg/mL) |
| r_min | Reaction Time (R, min) |
| k_min | Kinetic Time (K, min) |
| angle_degree | Angle (degree) |
| ma_mm | Maximum Amplitude (MA, mm) |
| a_mm | Amplitude (A, mm) |
| a30_mm | Amplitude at 30 minutes (A30, mm) |
| a60_mm | Amplitude at 60 minutes (A60, mm) |
| vwf_percent | von Willebrand factor (vWF, %) |
| fib_conc | Fibrinogen (FIB, mg/dL) |
| ddi_conc | D-dimer (D-Di, ug/mL) |
| ch50_percent | Complement Activity (CH50, %) |
| kng1_conc | Kininogen-1 precursor (KNG-1, pg/mL) |
| pf12_conc | Prothrombin fragment 1 and 2 (PF12, pg/mL) |
| syndecan1_conc | Syndecan-1 (SDC1, pg/mL) |
| tat_conc | Thrombin anti-thrombin complex (TAT, pg/mL) |
| tpa_conc | Tissue plasminogen activator (tPA, pg/mL) |
| total_pbl_number | Number of Total Peripheral Blood Leukocytes (PBL) |
| tregs_freq | Frequency of Tregs Among CD4+ T cells |
| cd69_cd4tcells_freq | Frequency of CD69+ CD4+ T cells Among CD4+ T cells |
| pd1_cd4tcells_freq | Frequency of PD-1+CD4+ T cells Among CD4+ T cells |
| hladr_cd4tcells_freq | Frequency of HLA-DR+CD4+ T cells Among CD4+ T cells |
| cd69_cd8tcells_freq | Frequency of CD69+ CD8+ T cells Among CD8+ T cells |
| pd1_cd8tcells_freq | Frequency of PD-1+CD8+ T cells Among CD8+ T cells |
| hladr_cd8tcells_freq | Frequency of HLA-DR+CD8+ T cells Among CD8+ T cells |
| hladr_nkt_freq | Frequency of HLA-DR+ NKT cells Among NKT cells |
| cd56dim_nk_freq | Frequency of CD56dim cells Among NK cells |
| cd56bright_nk_freq | Frequency of CD56bright cells Among NK cells |
| hladr_cd56dim_freq | Frequency of HLA-DR Among CD56dim cells |
| cd8_cd56dim_freq | Frequency of CD8+ Among CD56dim cells |
| hladr_cd56bright_freq | Frequency of HLA-DR Among CD56bright cells |
| cd8_cd56bright_freq | Frequency of CD8+ Among CD56bright cells |
| neut_pbl_freq | Frequency of Neutrophils Among PBL |
| lymph_pbl_freq | Frequency of Lymphocytes Among PBL |
| hladr_neut_freq | Frequency of HLA-DR+ Neutrophils Among Neutrophils |
| bcells_pbl_freq | Frequency of B cells Among PBL |
| cd4tcells_pbl_freq | Frequency of CD4 T cells Among PBL |
| cd8tcells_pbl_freq | Frequency of CD8 T cells Among PBL |
| nkt_pbl_freq | Frequency of NKT cells Among PBL |
| tregs_pbl_freq | Frequency of Tregs Among PBL |
| nk_pbl_freq | Frequency of NK cells Among PBL |
| classicmonos_pbl_freq | Frequency of Classical Monocytes Among PBL |
| nonclassicmonos_pbl_freq | Frequency of Nonclassical Monocytes Among PBL |
| cdc_pbl_freq | Frequency of conventional dendritic cells (cDC) Among PBL |
| pdc_pbl_freq | Frequency of plasmacytoid dendritic cells (pDC) Among PBL |
| baso_pbl_freq | Frequency of Basophils Among PBL |
| neut_pbl_freq_2 | Frequency of Neutrophils Among PBL |
| eos_pbl_freq | Frequency of Eosinophils Among PBL |
| hladr_bcells_mfi | HLA-DR MFI on B cells |
| hladr_classicmono_mfi | HLA-DR MFI on Classical Monocytes |
| cd86_classicmono_mfi | CD86 MFI on Classical Monocytes |
| hladr_nonclassicmono_mfi | HLA-DR MFI on Nonclassical Monocytes |
| cd86_nonclassicmono_mfi | CD86 MFI on Nonclassical Monocytes |
| hladr_cdc_mfi | HLA-DR MFI on cDC |
| cd86_cdc_mfi | CD86 MFI on cDC |
| hladr_pdc_mfi | HLA-DR MFI on pDC |
| cd86_pdc_mfi | CD86 MFI on pDC |
| cd16_classicmono_mfi | CD16 MFI on Classical Monocytes |
| cd16_nonclassicmono_mfi | CD16 MFI on Nonclassical Monocytes |
| cd11c_neut_mfi | CD11c MFI on Neutrophils |
| cd11b_neut_mfi | CD11b MFI on Neutrophils |
| cd66b_neut_mfi | CD66b MFI on Neutrophils |
| cd16_neut_mfi | CD16 MFI on Neutrophils |
| cd62l_neut_mfi | CD62L MFI on Neutrophils |
| mean_fsc_neut | Mean FSC on Neutrophils |
| mean_ssc_neut | Mean SSC on Neutrophils |

**Appendix A –**

Clinical Trial Protocol - TAMPITI Protocol version #12_7.22.16

**Tranexamic Acid Mechanisms and Pharmacokinetics In Traumatic Injury (TAMPITI Trial)**

**Background:**

Trauma is the leading cause of death in persons younger than 40 years. Hemorrhage is the etiology in 30% of these deaths, and remains the leading cause of potentially preventable mortality (66-80%) [[1](#_ENREF_1)]. As a result, the prevention of death from hemorrhagic shock has been the subject of intensive research and effort. Death secondary to hemorrhagic shock occurs from both surgical bleeding and coagulopathy. Acute traumatic coagulopathy is characterized by a hypocoagulable state, where the net balance of coagulation is such that there is low clot forming capacity and strength, which does not allow for adequate hemostasis. Acute traumatic coagulopathy occurs early in patients who are in shock from hypoperfusion and is not due to coagulation factor consumption or dysfunction because of acidosis, moderate hypothermia, or dilution[[2](#_ENREF_2)]. However shock (oxygen debt) itself is associated with a coagulopathy that is due to the systemic activation of anticoagulant and fibrinolytic pathways[[1](#_ENREF_1), [3](#_ENREF_3)]. The protein C pathway is implicated in this process, in addition to fibrinolysis which is mediated by de-inhibition of tPA through PAI-1 consumption[[2](#_ENREF_2)]. Low levels of PAI-1, with increased plasminogen activator release from the vessel wall contributes to hyperfibrinolysis. It has been suggested that TAFI is the main driver of fibrinolysis inhibition, and that reduction in TAFI activation by the competitive binding of protein C to T-TM is the mechanism for increased fibrinolysis with activation of protein C [[1](#_ENREF_1)].

Due to the knowledge of increased fibrinolysis promoting a hypocoagulable state in severe trauma, trials have been performed to determine if antifibrinolytics such as tranexamic acid (TXA) could reduce morbidity and mortality by reducing death from hemorrhage[[4](#_ENREF_4)]. TXA is an antifibrinolytic that inhibits both plasminogen activation and plasmin activity, thus preventing clot break-down rather than promoting new clot formation. TXA occupies the lysine-binding sites on plasminogen, therefore preventing its binding to lysine residues on fibrin. This reduces plasminogen activation to plasmin. Similarly, blockade of lysine-binding sites on circulating plasmin prevents binding to fibrin and thus prevents clot break-down. TXA is excreted largely unchanged in urine and has a half-life of approximately 2 hours in circulation when studied in patients without traumatic injury. Intravenous administration of TXA was approved by the FDA in 1986 for the prevention or reduction of bleeding in patients with hemophilia undergoing dental procedures. The FDA approved use of the oral form of TXA to control heavy menstrual cyclic bleeding in 2009. Despite the extensive and routine use of TXA in many surgical populations and an increasing use in severe trauma patients, TXA does not have an FDA approved indication for patients with traumatic injuries[[1](#_ENREF_1)].

In 2010, the results of the landmark CRASH-2 (Clinical Randomization of an Antifibrinolytic in Significant Hemorrhage) trial were published, creating widespread international interest [[5](#_ENREF_5)]. This multicenter, multinational study randomized 20,211 adult trauma patients to either one gram of TXA infused over ten minutes followed by one gram of TXA infused over eight hours versus an equivalent volume of normal saline placebo administered within eight hours of injury. Inclusion criteria consisted of systolic blood pressure less than 90 mmHg and/or with a heart rate greater than 110 beats per minute, or patients deemed to be at risk of significant hemorrhage. The primary outcome measure was in-hospital death within four weeks of injury. Secondary outcomes included thromboembolic events (myocardial infarctions, cerebrovascular accidents, pulmonary emboli, and deep vein thromboses), surgical interventions, blood transfusions, and the total units of blood transfused. The study found a significant decrease in all-cause mortality (14.5 vs. 16.0%, p=0.0035) and deaths from bleeding (4.9 vs. 5.7%, p=0.0077) in patients receiving TXA compared to placebo. More recently, data from the Military Application of Tranexamic Acid in Trauma Emergency Resuscitation Study (MATTERs) has been published, providing perspective from a different and likely more severely injured patient population[[6](#_ENREF_6)]. MATTERs is a retrospective observational study that evaluated data from the Camp Bastion Surgical Hospital in Afghanistan. Patients were included in the study if they sustained combat-related injury and subsequently received a minimum of one unit of packed red blood cells. The primary outcome measures were 24 and 48-hour as well as in-hospital mortality (which was inclusive of any mortality occurring within 30 days of injury). Secondary endpoints included transfusion requirement and correction of coagulopathy based on resolution of extended prothrombin and thromboplastin times between arrival in the emergency department and arrival in the intensive care unit postoperatively. Overall, 896 consecutive patients were analyzed, with 293 receiving TXA. Patients receiving TXA were more severely injured than those not receiving the drug (ISS 25.2 vs. 22.5, p<0.001), and a greater proportion presented with severe TBI and admission systolic blood pressure ≤ 90 mmHg. Patients receiving TXA were given more packed red blood cells, fresh frozen plasma, platelet, and cryoprecipitate transfusions than patients not receiving the drug. In spite of these admission characteristics, patients receiving TXA had lower overall 48-hour (11.3 vs. 18.9%, p=0.004) as well as in-hospital (17.4 vs. 23.9%, p=0.03) mortality. Additionally, although more patients who received TXA were hypocoagulable on admission, fewer TXA patients were hypocoagulable on arrival to the ICU, and there was a significant reduction in the proportion of hypocoagulable patients in the TXA group between admission and the ICU. While these data add to the beneficial profile of TXA, MATTERs also reported a greater number of pulmonary emboli (8 vs. 2, p=0.001) and deep vein thromboses (7 vs. 1, p=0.001) in TXA-treated patients.

Despite both the CRASH-2 trial and MATTERS studies indicating TXA use reduced the risk of mortality, questions regarding mechanisms of action still remain. Interestingly, in CRASH-2, while improved survival was due to decreased death from hemorrhage, half of the patients did not require blood transfusions or surgical intervention. This would suggest that correction of coagulopathy was not the only mechanism contributing to improved outcomes in this trial. Figure 1 describes plasmin’s multiple effects to include leukocyte activation and chemokinesis. Given that TXA’s primary effect is purported to be inhibition of plasminogen activation preventing plasmin generation, evaluating monocyte and neutrophil activity and downstream effects in the adaptive immune response in the setting of TXA administration are required. The immune suppressive effects of TXA on immune function have not been thoroughly examined, especially in patients with severe traumatic injury. It is possible that the improved outcomes that have been reported with early administration of TXA in trauma patients are related to its immune suppressive effects. The study of the effects of TXA use on endothelial activation and injury is also important due to the inter-relationship between coagulation and endothelial function. Endothelial injury secondary to local hypoperfusion causes acute traumatic coagulopathy with fibrinolysis. Therefore a thorough and comprehensive evaluation of the effects of TXA on immune, coagulation, and endothelial parameters is important to allow for a better understanding of the mechanisms of action of this agent.

**Figure 1:** Trauma and fibrinolysis

**
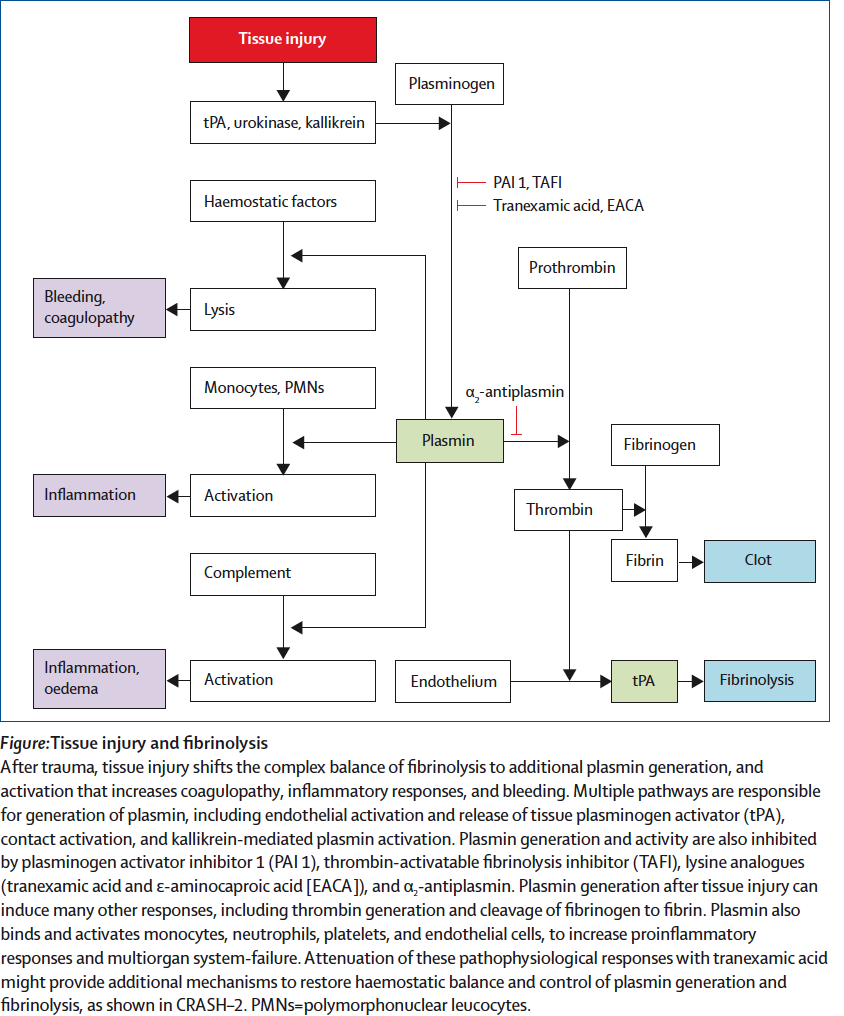
**

Optimal dosing is also unknown for patients with traumatic bleeding. The lack of pharmacokinetic studies in this population contributes to the uncertainty regarding TXA dosing for patients with traumatic injury and hemorrhage. While the CRASH-2 trial utilized a bolus dose followed by an 8-hour infusion, the MATTERS study used multiple IV bolus doses without an 8-hour infusion. The intravenous dosing of TXA for patients with hemophilia with dental bleeding, which is the only FDA approved indication for IV administration, is an IV bolus dose of 10mg/kg every 6 to 8 hours one day prior to surgery. The FDA approved indication for the intravenous form of TXA does not include a continuous infusion dose over 8 hours after a bolus dose. The common, but off label use of TXA in multiple surgical populations often utilizes an IV bolus dose followed by an 8-hour continuous infusion dose. However, in a trauma patient with potentially limited vascular access, a one-time IV bolus dose would be more practical and may facilitate increased plasma concentration in the immediate time period where hemostatic control is critical. The CRASH-2 trial utilized a total dose of 2mg/kg IV, and the MATTERS study utilized doses that ranged between 1-4 mg/kg IV (personal communication with Dr Joseph J. Debose). Therefore the analysis of multiple bolus doses (within range of current practice) on mechanisms and pharmacokinetics is important and especially relevant to the practical application of TXA. It is critical for formal and thorough pharmacokinetic analyses to be done in patients with traumatic injury with active bleeding since this population has different physiology than elective surgery patients. The pharmacokinetic analysis of multiple bolus doses in addition to mechanistic, outcomes and safety data will inform the medical community regarding the most appropriate dosing of TXA in the acutely injured, critically ill, trauma patient.

In addition to the potential risk of thrombosis from the use of TXA, seizures have also been recognized as a possible adverse effect. The proposed mechanism for seizures is the structural similarity of TXA to γ-aminobutyric acid, which has a potential to cause neurotoxicity. TXA use in cardiac surgery has been associated with an increased risk of postoperative convulsive seizures. [85] These findings are associated with TXA doses that are well above the 2 and 4-gram IV doses proposed in our trial. For a typical 70 kg patient, a 4-gram dose of TXA is 57 mg/kg. A recent study of over 1000 patients requiring cardiac surgery indicated that doses > 100mg/kg or 7 grams IV were associated with an increased risk of early seizure. [85]It is important to recognize that while the risk of early seizure was increased at doses > 100mg/kg the overall incidence of seizure was still very low at 1.3% of patients. For the typical 70 kg patient in this study that received a dose of 4 grams of TXA IV, the probability of seizure was 0.01%, with a narrow 95% CI of (0.006-0.014. [85]

A currently unpublished survey of all US trauma centers by our research group that had a 132/187 (71%) response rate, indicated 52% of level one US trauma centers incorporate TXA into their massive transfusion protocols. This data demonstrates that equipoise exists regarding its routine use in this population. Therefore it is essential and appropriate that prospective randomized control trials are performed to provide evidence regarding its mechanism of action data, pharmacokinetic information, and efficacy and safety data.

**Hypotheses:**

1. We hypothesize that early TXA use in patients with severe traumatic injuries, reduces a pro-inflammatory state and monocyte activation. To test this hypothesis in 150 patients (50 in each study group), we will determine the effects on immune function measures, at multiple time points, in all three study groups. We expect reduced inflammation, and monocyte activation in TXA treated patients compared to placebo.

2. We hypothesize that the pharmacokinetics of TXA administration are affected by the degree of shock measured by admission lactate, StO2, presence of acute renal failure, and blood products administered in patients with severe traumatic injury. We expect that the degree of shock, renal function, and blood products transfused will affect TXA pharmacokinetics.

3. We hypothesize that early use of TXA is safe and associated with improved outcomes. We expect that the use of TXA at both 2-gram IV and 4-gram IV doses will be safe and will not be associated with an increased risk of thromboembolic events, ARDS, seizures or any adverse events compared to placebo.

**Aim 1:** To determine the effects of TXA on immune parameters.

To evaluate the effects of TXA on immune function parameters we will, in a RCT, analyze samples from 150 patients (50 in each study group), at multiple time points. Parameters are:

a. Cytokines: Including but not limited to: TNF-α, IL-6, IL-10, and IFN-γ measured at approximately 0, 6, 24 and 72 hours.

b. Flow cytometric analyses and stimulation tests to determine function on the following cells: polymorphonuclear cells, lymphocytes, monocytes, and T regulatory cells measured at approximately 0, 6, 24 and 72 hours:

**Aim 2:** To determine the pharmacokinetics and pharmacodynamics of multiple TXA dosing regiments.

To determine if the pharmacokinetics of TXA administration are affected by the degree of shock measured by admission lactate, StO2 (measured by NIRS), presence of acute renal failure, and blood products administered in patients with severe traumatic injury, we will perform the following analyses in a total of 100 patients within both TXA treated groups (50 patients per TXA treatment group).

a. Perform pharmacokinetic analyses

b. Compare pharmacokinetic data between patients with varying degrees of shock (lactate and StO2 measures) and according to total amount of blood products transfused in the first 12 hours of injury.

**Aim 3:** To collect safety and clinical outcomes data on TXA use in patients with traumatic injuries.

To measure safety and outcome data in this RCT of 150 patients with severe trauma we will:

a. Determine the incidence of thromboembolic events (DVT, MI, PE, Stroke) in all three study groups. Subjects will be assessed daily by the study team until hospital discharge or up to 30 days, (which ever comes first) after receiving study drug for any signs and symptoms of a thromboembolic event (i.e. shortness of breath, chest pain, extremity swelling, fever of unknown etiology, etc.) In addition, the study team will further ensure the safety of our subjects by screening all subjects on hospital day 7 (+/- 2 days) or at hospital discharge (- 2 days), whichever comes first for DVT using duplex ultrasonography. In cases where a subject is possibly being discharged from the hospital over the weekend/holiday (communicated by the clinical care team to the study team), a duplex ultrasound will be ordered to be performed on the Friday/day before the holiday to help ensure that it is performed, regardless what hospital day it is. In addition, if a subject has a plan for discharge, he/she will have a duplex performed as soon as possible regardless of what hospital day it is. Stroke, MI, and PE will each be diagnosed clinically according to standard definitions (see appendix at end of document).

b. Determine the incidence of seizures at 24 hours in all three study groups.

c. Determine the incidence of all adverse events in all three study groups. Adverse events for up to 30 days after study drug administration will be characterized according to severity, relatedness, duration, and resolution.

d. Compare mortality, mechanical ventilation and ICU free days, incidence of multiple organ failure, Acute Kidney Injury (AKI), Acute Respiratory Distress Syndrome (ARDS) and sepsis in all three study arms.

**Aim 4:** To develop a repository of blood samples for future analysis.

These banked samples would be analyzed for measures of coagulation, white blood cells and endothelial function to include but not limited to:

1. Complement: CH50 measured at approximately 0, 6, 24 and 72 hours
2. Endothelial activation and injury: Soluble Flt-1, soluble E-Selectin, VCAM-1, and PAI-1, Ang1/Ang2, Syndecan 1, Syndecan 2 and vWF measured at approximate time points 0, 1, 6, 24 hours.
3. Coagulation and Fibrinolysis:
   1. Inhibition of plasmin activity by TXA: D-dimer, PAP, alpha2-antiplasmin, TAFI, measured at approximately 0, 1, 6, and 24 hours
   2. Activation and inhibition of fibrinolysis: tPA and PAI-1 measured at approximately 0, 1, 6, and 24 hours
   3. Activation of the coagulation system: thrombin generation assays, TAT complexes, antithrombin, fibrinogen, fibrin monomer, fibrinopeptide A and B, Factor XIII, Factor XI, measured at approximately 0, 1, 6, and 24 hours

**Methods**:

Research Design: A single center, double- blind, randomized, placebo-controlled study will be performed under an IRB approval for exception from informed written consent (with community consultation) and with IND approval from the FDA. Participants will be randomized into 1 of 3 treatment arms (1:1:1): TXA 2 gram IV bolus, TXA 4 gram IV bolus, or placebo. A transfusion guideline will be in place to standardize transfusion practice in this trial. Investigators will request that additional antifibrinolytics (i.e. TXA, Aminocaproic Acid) **NOT** be administered to participants who have been enrolled in this study and have already received study drug for safety purposes.  Administration of additional doses of antifibrinolytics will only be permitted if it is deemed necessary for the clinical care of the patient.  In such cases, the clinical care team will be required to contact a study Investigator to request their permission to allow a clinical care dose of TXA or other antifibrinolytic be administered to a study patient.  If the Investigator provides permission for the additional antifibrinolytic, the participant will remain on study and the information surrounding this event will be captured and included in the participant’s study record. Any participant who receives additional antifibrinolytics will continue participation in the study and will be followed closely for safety purposes.

Study period: The study period is from time of enrollment to hospital discharge or transfer. The study intervention will occur only once upon enrollment in the trial.

Power Analysis: Recent data indicates that in severely injured trauma patients 6.2 ± 0.8% of monocytes express CD14 at 72 hours post injury [86].  We consider a 10% decrease in CD14+/HLA-DR+ monocytes from 6 ± 0.8% to 5.4 ± 0.8% to be potentially clinically relevant. Therefore, using a two-tailed α of 0.05 and a (1-β) of 0.90, and a very conservative 20% dropout rate, a total of 47 patients per arm or a total of at least 141 patients will be required. To account for patients that may have missing data we plan to enroll 50 patients in each study group or a total of 150 patients.

Inclusion criteria:

1.) Patients with traumatic injury that are ordered to receive at least 1 blood product

and/or

2.) Patients admitted to the Emergency Department with a traumatic injury and require immediate transfer to the operating room to control the bleeding

3.) Able to receive the study drug within 2 hours from estimated time of injury

**Please note that in circumstances where the patient initially met inclusion/exclusion criteria (i.e. received blood products in the ED before a full evaluation of their injuries is complete) but is later found to only have a soft tissue involved injury or does not have a traumatic bleeding source), the Investigator may determine that the patient should not be randomized into the trial and the patient should be considered a screen failure

Exclusion criteria:

1. Patients known to be < 18 years of age
2. Suspected acute MI or stroke (thromboembolic and/or hemorrhagic) on admission
3. Known inherited coagulation disorders
4. Known past medical history of thromboembolic events (DVT, PE, MI, Thromboembolic Stroke)

- Please note that past medical history of hemorrhagic stroke is permitted, but not current admission with hemorrhagic stroke

1. Known history of seizures and/or seizure after injury/on admission related to this hospitalization
2. Suspected or known pregnancy
3. Known to be lactating
4. Suspected or known prisoners
5. Futile care
6. Known current state of immunosuppression (i.e. on high dose steroids, chemotherapeutics, etc.)
7. Unknown estimated time of injury

12.) Patients wearing an “Opt Out” TAMPITI Study bracelet

13.) Known presence of subarachnoid hemorrhage

14.) Isolated injuries to hands and/or feet (distal)

15.) Administration of antifibrinolytics pre-hospital and/or during this ED admission prior to enrollment

Randomization: This study is a placebo controlled (1:1:1), double-blinded, randomized clinical trial of 150 severely injured trauma patients. Participants will be randomized (1:1:1) to TXA dose 1 (2 gram IV bolus), TXA dose 2 (4 gram IV bolus), and matching volume of Normal Saline placebo. The Statistician will randomize patients to treatment assignment using RedCap and will provide the master randomization assignment list to the Investigational Research Pharmacy who will dispense the study drug according to treatment assignment. The study drug dispensed will only include the randomization # and the treatment assignment will remain blinded to study staff and the clinical care team. The study team member responsible for overseeing the study drug administration will verify that the study drug code matches the assigned code for the specific participant to ensure that the **right patient gets the right drug**. Once study drug is obtained by the pharmacy, it will be administered (blinded) by IV over approximately 10 minutes. Study drug will not be administered faster than over 10 minutes, but the additional time it takes to prime and flush the IV tubing for study drug administration will add a few minutes (~ 3-4 additional minutes) to the total time it takes for the study drug to be completely administered.

Blinding: Since this is a double-blinded study, study investigators and team members (with the exception of the study statistician) will be blinded to treatment assignment. In addition to the statistician, the only individuals who will know the study group assignment will be the Investigational Pharmacists and their designees and up to three unblinded study team members who will be responsible for sorting PK/PD samples in order to only analyze the samples of participants that received TXA. The Investigational Pharmacists can reveal the subject’s treatment assignment for safety or treatment concerns. Accidental and suspected un-blinding will be considered a protocol deviation and will be submitted to the IRB according to Institutional policies and procedures. Data from subjects who were unblinded will be included in the intent-to-treat analysis.

Treatment groups: The three treatment arms will be TXA dose 1 (2 gram IV bolus), TXA dose 2 (4 gram IV bolus), and matching volume of Normal Saline placebo.

Primary outcomes: Primary outcomes include differences in the proportion of activated monocytes among the 3 treatment arms (TXA dose 1, TXA dose, 2, and placebo) from time 0 to time 72 hours.

Secondary outcomes: Secondary outcomes measures include differences in cytokine profiles and leukocyte function parameters, clinical outcomes, and PK analyses between the three study groups (as detailed in Aims 1-4 below). These comparisons will be exploratory and because of the large number of such comparisons, analytic results will be interpreted cautiously.

Patient screening: During the patient recruitment phase of the trial there will be research coordinators in house 24 hours a day, 7 days a week to facilitate constant patient screening and immediate determination of eligibility, initiate the randomization process, ensure blood sample acquisition and processing, and prospective data collection. All research coordinators will carry a trauma pager and will be present in the Emergency room to screen and enroll study patients. An on-call system for additional research laboratory staff will be funded to facilitate trial execution. In order to detect any potential biases, a screening log will be maintained to record the number of eligible patients, the number of patients eligible not randomized, and the reason for their exclusion. This should allow detection of any selection bias.

Anticipated cohort and recruitment rate: Trauma Registry data from 2011 report an incidence of 150 patients per year that met eligibility criteria for this trial. These patients have a mean age of 40, ISS of 16, and in hospital mortality of 18%.

Data Collection: The schedule of events details timing of study related procedures, including timing and volume of blood samples being collected (See Appendix D). The 27 ml of blood listed for PK studies is the total volume collected in 24 hours at multiple time points. Data to be collected on all patients will include epidemiologic data, diagnoses, surgical procedures, blood products (RBC, plasma, platelets, cryoprecipitate), coagulation factor concentrates, concomitant medications, vital signs, ST02 data, clinical labs, AIS/ISS, presence of acute renal failure according to RIFLE criteria [[78](#_ENREF_78)], and clinical outcomes. Details regarding medication administration, blood products and coagulation factor concentrates will be recorded. Storage duration, additive solutions and processing methods used for blood products will also be recorded. Seizure, severe adverse thrombotic events, and all adverse events will be captured from the time of study drug administration until hospital discharge or up to 30 days, (which ever comes first) although the primary analysis of the risk of seizure and adverse thrombotic events will most likely occur within the first 24 hours and 7 days of the trial respectively. The risk of seizure secondary to TXA has never been reported to occur after the first 24 hours post TXA administration [[76](#_ENREF_76)].

Clinical outcomes measured will include mortality at 24 hours, 30 days, and hospital discharge/transfer, ventilator and ICU free days, AKI, incidence of multiple organ failure and sepsis/severe sepsis between TXA treated and placebo study groups. Multiple organ failure will be defined according to the Marshall Score (See Appendix A). Sepsis and ARDS will be defined according to standard definitions (See Appendix A). Clinical and safety data will be collected daily until day of hospital discharge or transfer.

Banking of patient samples: All samples collected for banking for future analysis will be frozen at -80 degrees Celsius. Samples will be cataloged and appropriately labeled. The repository of stored blood samples collected from participants in this study will be analyzed in the future, if additional funding is obtained, to help us understand how severe trauma effects coagulation and endothelial function and the effects TXA has on coagulation and endothelial function. Please note that banked samples will not be used for DNA analysis.

Protocol Deviations: Patients will be considered adherent to the protocol only if they receive TXA at the appropriate dose or placebo as intended by the randomization process. Patients who receive the wrong TXA dose or TXA instead of placebo or placebo instead of TXA will not be adherent to the protocol and will be classified as protocol deviations. Patients not adherent to protocol will be included in the intent-to-treat analysis but will be excluded from the per-protocol analysis. The decision to withdraw care will not be considered an exclusion criterion, if made after patient entry. These cases will be kept in the ITT analysis.

**Laboratory Methods:**

**Aim 1:** To determine the effects of TXA on immune parameters. To evaluate the effects of TXA on immune function parameters we will, in an RCT, analyze samples from 150 patients (50 in each study group), at multiple time points. Parameters to be measured are:

a. Cytokines: Including but not limited to: TNF-α, IL-6, IL-10, and IFN-γ measured at approximately 0, 6, 24 and 72 hours.

b. Flow cytometric analyses and stimulation tests to determine function on the following cells: polymorphonuclear cells, lymphocytes, monocytes, and T regulatory cells measured at approximately 0, 6, 24 and 72 hours:

Approximately 6 mL whole blood samples will be collected at approximately 0, 6, 24 and 72 hours later and stored at -80C. Samples will later be processed and measured for cytokine, etc. as described above.

Aim 1 Statistical analyses: The outcome measures in this aim are continuous variables that will be measured at multiple longitudinal time points.

**Aim 2:** To determine the pharmacokinetics and pharmacodynamics of multiple TXA dosing regiments.

To determine if pharmacokinetics of TXA administration are affected by the degree of shock measured by admission lactate, StO2 (measured by NIRS), presence of acute renal failure, and blood products administered in patients with severe traumatic injury, we will perform the following analyses in a total of 150 patients within both TXA treated groups (50 patients per TXA treatment group).

a. Perform pharmacokinetic analyses

b. Compare pharmacokinetic data between patients with varying degrees of shock (lactate and StO2 measures) and adjusting for acute renal failure and total amount of blood products transfused in the first 12 hours of injury.

To facilitate the frequent sampling of patients for this PK analysis, research staff will be present at the clinical site 24 hours a day, 7 days a week with an on-call system for additional research laboratory support when needed. The research staff will follow subjects from admission throughout their hospitalization. Patient blood sampling schedules will be alternated (in order, every other patient once enrolled: even schedule vs. odd) to reduce the volume of blood phlebotomized from patients. Research staff will only sample the even or odd number time points from individual patients. For example if a patient is sampled according to even number sampling times, blood will be drawn at the approximate time points: 0, 20 min, 1 hr, 2 hr, 4 hr, 6 hr, 8 hr, and 12 hr. A patient sampled on odd number sampling times will have samples drawn at the approximate time points: 0, 10 min, 40 min, 1.5 hr, 3 hr, 6 hr, 10 hr and 24 hr.

Each blood sample will be collected, centrifuged and the supernatant (plasma) will be stored at -80C before analysis. Samples (stripped of any identifiers and only containing the subject’s study number) will later be processed for full pharmacokinetic analysis. TXA will be extracted from plasma samples using solid phase microextraction, and the concentrations will be measured using tandem liquid chromatography- mass spectrometry [[77](#_ENREF_77)]. Pharmacokinetic data will be analyzed with NONMEM, using both the first-order and conditional non-Laplacian (with centering) estimation techniques. We will consider two- and three-compartment models, parameterized in terms of both compartment volumes and clearances (distribution and elimination). We will compare a basic model (in which pharmacokinetic parameters were independent of weight) to a model in which the pharmacokinetic parameters will be assumed to be proportional to weight. The optimal model will be selected on the basis of the objective function logarithm of the likelihood of the results) using standard criteria (NONMEM guide).

Aim 2 Statistical Analysis: The pharmacokinetic and pharmacodynamic (henceforth, PK/PD) analysis will be performed using pooled data from the entire study population. This will be accomplished by nonlinear mixed effects modeling, using the software package NONMEM (version 7.2.0). The basic features of NONMEM may be best understood by considering a typical PK experiment. In the typical pharmacokinetic experiment, the drug is given to a subject, blood samples are drawn at defined intervals after dosing, the blood concentration is determined as a function of time, and a compartment model is then “fit” to the data to derive pharmacokinetic parameters for individual patients. To understand pharmacokinetic variability among patients, this same approach will be repeated in multiple subjects. One can then simply calculate the mean of the individual pharmacokinetic parameters and also their variances or standard deviations. This approach is called the two-stage method and it is laborious, time consuming, and logistically challenging since it requires enough samples from each patient to determine their individual PK parameters. An alternate approach to determining the pharmacokinetic parameters for multiple patients and then averaging them is to draw a small number of samples from a large number of patients (after drug administration) and then perform what is termed a “mixed-effects” analysis on the pooled data. The difference between the observed and the predicted concentration is attributed to 1) measurement error and 2) the individual variation of the patient’s pharmacokinetic parameters from the mean value for the population. For any observation, we can define the “likelihood” of the difference of the observed concentration and that predicted by the model. The likelihood is essentially the probability that if the model were true given the present values of the model parameters, the present observations would have been observed. If the observations are quite unlikely for the present model, then the present model is not a very good description of the observations. If the observations are quite likely for the present model, then the present model is most likely a valid explanation of the data.

We will begin our analysis comparing one, two, and three compartment models. The minimization of the objective function (in other words, the maximization of the above expression for the likelihood) is mathematically and computationally complex. We will select as parameters for the PK model the compartment volumes and intra-compartmental and elimination clearances. The one compartment model would be characterized by a single compartment volume and the elimination clearance (two “structural” parameters), the two-compartment model by the central and peripheral compartment volumes, the intra-compartmental clearance, and the elimination clearance (four structural parameters), and comparably for the three-compartment model (which will have six structural parameters). We will compare the minimal objective function for the one, two, and three compartment models. Given that the objective function has a χ^2^ distribution, one would select a model with an additional parameter only if the objective function improves, i.e., decreases, by more than 3.84 to achieve significance at the p=0.05 level. Thus, we would select as our basic model the two-compartment model only if the objective function improves by more than 7.68 and similarly for selecting a three-compartment model rather than a two-compartment model. In addition to this basic statistical test, we will also reject more complex models if the minimization does not converge with a full covariance matrix (in general we can anticipate that parameters will not only have a variance, but that there will be covariance between parameters). We will also graphically analyze the models by plotting residuals (difference between predicted and observed concentrations) vs. time and predicted concentration to look for systematic (nonrandom) variation. With mixed-effect modeling one can determine empiric (post-hoc) Bayes estimates of individual patient parameters. These are the most probable parameter estimates given the estimate of the mean parameter, the variance of the parameter, and the observed drug concentrations in individual patients. This allows us to compare observed drug concentrations to drug concentrations predicted for the individual patient, as well as the drug concentrations predicted with mean parameters. We will reject more complex models if they result in a marked increase in residuals or systematic variation in the residuals.

In addition to determining the optimal basic model, the first stage of analysis will also require consideration of weight-based pharmacokinetic parameters. It is intuitive that pharmacokinetic parameters will be proportional with body mass in some manner. However, it is well known in PK analysis that if there is insufficient heterogeneity in the body mass of patients in the study group, weight-adjusted parameters lead to no better a fit of the data than non weight-adjusted parameters. After establishing the optimal compartmental model (1, 2, or 3 compartments), we will compare models in which we assume that compartment volumes and clearances are proportional to weight to those in which weight is not a factor.

Following determination of the optimal compartment model and whether we need weight-based modeling, we will explore the role of covariates. We will evaluate as potential covariates age, degree of shock as assessed by admission lactate and StO2, presence of renal failure, and blood products administered using this approach of graphical analysis of the relationship between the covariate and the specific PK parameter and then formal modeling of the parameter as a linear function of the covariate. In summary our PK/PD analysis will entail the following methods and steps

1. Use of pooled data and nonlinear mixed effects modeling using NONMEM

2. Initial analysis using the first-order approximation and estimation algorithm

3. Determination of the optimal compartment model (1, 2, or 3 compartments parameterized in terms of compartment volumes and clearances).

4. Selection of the optimal model using the χ^2^ distribution of the objective function and also graphical inspection of the residuals for detection of systematic variation.

5. Evaluation of weight-based analysis.

6. Analysis of covariates (age, lactate, StO2, and volume of blood products) by graphical analysis of the relationship between the relevant η and the covariate and then inclusion of the covariate in the formal model and rejection of the covariate as significant based on the χ^2^ distribution of the objective function.

7. Analysis of pharmacodynamic models using a sigmoid-E_max_ model and nonlinear mixed effects modeling.

**Aim 3:** To collect safety and clinical outcomes data on TXA use in patients with traumatic injuries.

To measure safety and outcome data in this RCT of 150 patients with severe trauma we will:

a. Determine the incidence of thromboembolic events (DVT, MI, PE, Stroke) in all three study groups. Subjects will be assessed daily by the study team (while in the hospital) for up to 30 days after receiving study drug for any signs and symptoms of a thromboembolic event (i.e. shortness of breath, chest pain, extremity swelling, fever of unknown etiology, etc.) In addition, the study team will further ensure the safety of our subjects by screening all subjects at day 7 (+/- two days) or hospital discharge (- 2days) (if prior to 7 days) for DVT using duplex ultrasonography of the lower extremities. In cases where a subject is possibly being discharged from the hospital over the weekend/holiday (communicated by the clinical care team to the study team), a duplex ultrasound will be ordered to be performed on the Friday/day before a holiday to help ensure that it is performed, regardless what hospital day it is. In addition, if a subject has a plan for discharge, he/she will have a duplex performed as soon as possible regardless of what hospital day it is. Stroke, MI, and PE will each be diagnosed clinically according to standard definitions (see appendix A).

b. Determine the incidence of seizures at 24 hours in all three study groups.

c. Determine the incidence of all adverse events in all three study groups. Adverse events will be captured daily while subject remains in the hospital or for up to 30 days, (whichever comes first) and will be characterized according to severity, relatedness, duration, and resolution.

d. Compare mortality, mechanical ventilation and ICU free days, incidence of multiple organ failure, AKI, ARDS and sepsis in all three study arms.

e. Correlate mechanistic data collected with clinical outcomes measured.

Seizure, thromboembolic events, and all adverse events will be captured from the time of study drug administration until discharge/transfer from the hospital or for up to 30 days (whichever comes first). ICU and ventilator free days will be calculated according to a 30-day model.

Aim 3 Statistical Analysis: The incidence of thromboembolic events will be determined at 7 days or on the day of hospital discharge if the hospital stay is less than 7 days. Incidence of seizures will be determined 24 hours from time of randomization. We expect that approximately 20% of the sample will being diagnosed with a thrombotic event based on previous work in a similar cohort of routinely screened transfused trauma patients by Spinella [[28](#_ENREF_28)]. Because most of these events will be asymptomatic, we will not be able to determine the specific day on which the event occurred. Thus, we will be unable to use survival models for this outcome. Instead, chi square tests will provide tests of the null hypothesis that rates are identical across groups. These will be followed by logistic regression analyses that adjust for appropriate and a priori determined covariates. To be conservative, subjects who die before 7 days will be assumed to have had a thrombotic event in our primary analysis. While there is no published literature on the risk of seizure in a cohort of transfused trauma patients, we estimate that it will occur in approximately 2% of the study population. Because we will know the day on which this occurred, survival methods will be applied to this variable, with the subject being censored on day 7 or the day of death or hospital discharge when either occurs prior to 7 days. Log rank tests will be used to compare survival curves across groups and Cox regression analyses will provide an adjustment for covariates. Secondary outcomes in this aim include mortality, which will be evaluated using the log rank test and Cox regression and the incidence of multiple organ failure and sepsis, which will be assessed using chi square tests and logistic regression. We will most likely apply Poisson regression models to outcomes such as the number of severe and related adverse events. However, a final determination of the appropriate method will have to await an evaluation of the pattern and frequency of occurrence of these events.

**Aim 4:** To develop a repository of blood samples for future analysis.

Blood samples will be drawn at the following approximate time points: 0, 1, 6, 24, and 72 hours, centrifuged and stored at -80 degrees Celsius.

These banked samples would be analyzed for measures of coagulation, White Blood Cells, and endothelial function to include but not limited to: Complement, CH50 measured at approximately 0, 6, 24 and 72 hours; Endothelial activation and injury, Soluble Flt-1, soluble E-Selectin, VCAM-1, and PAI-1, Ang1/Ang2, Syndecan 1, Syndecan 2 and vWF measured at approximate time points 0, 1, 6, 24 hours. Banked samples for future analysis at approximate times 0, 1, 6, and 24 hours; Inhibition of plasmin activity by TXA: D-dimer, PAP, alpha2-antiplasmin, TAFI, Activation and inhibition of fibrinolysis: tPA and PAI-1, Activation of the coagulation system: thrombin generation assays, TAT complexes, antithrombin, fibrinogen, fibrin monomer, fibrinopeptide A and B, Factor XIII, Factor XI.

**Adverse Events**

TXA has been used for decades and possesses a well-established safety profile. While there have been rare cases of thromboembolic events and seizures potentially associated with high dose (> 100mg/kg) intravenous TXA administration, there is no evidence that the TXA treatment regimens proposed in this trial (maximum 57mg/kg for a 70 kg patient) will be associated with an increased risk of thromboembolic events or seizures. We will collect data on these events and will report such occurrences to the DSMB according to the DSMP (see Appendix B). Emergency un-blinding will be immediately available if deemed necessary by the clinical care team, DSMB, or Investigators for the safety or treatment related decisions during the course of the study. The IDS pharmacists or their designee/s will be able to un-blind in such situations.

Adverse events will be collected daily on all subjects from study drug administration until hospital discharge or up to 30 days, (which ever comes first)**.**

**Adverse Event Definitions**

- **Adverse event (AE):** is any untoward medical occurrence in a study subject. An AE can therefore be any unfavorable and unintended sign, symptom or disease temporally associated with the use of an investigational product, whether or not considered related to the investigational product.
- **Suspected Adverse Reaction:** is any adverse event for which there is a reasonable possibility that the drug caused the adverse event. For IND safety reporting reasonable possibility means that there is evidence to suggest a causal relationship between the drug and the adverse event.
- **Adverse Reaction:** any adverse event caused by a drug**.**
- **Serious Adverse Event:** an adverse event is considered serious if it results in any of the following outcomes:
  - Results in death
  - Considered to be life-threatening
  - Requires hospitalization or prolongation of existing hospitalization
  - Results in persistent or significant disability or incapacity
  - Results in a congenital anomaly or birth defect
- **Life-threatening** refers to an event in which the subject was at risk of death at the time of the event; it does not include an event that might have caused death if it were more severe. All other events that are considered medically serious by the investigator should also be reported.

**Adverse Event Assessment and Reporting**

Participants with traumatic injuries requiring intensive care unit admissions are expected to have adverse events and abnormal laboratory findings related to their medical condition. Investigators will review adverse events to determine their relationship to the participant’s medical condition. Those adverse events associated with the underlying disease will only be reported to the HRPO, DoD HRPO, and FDA in an expedited manner if the PI or co-investigator of the study site feels that the event is UNEXPECTED AND RELATED to study drug. Adverse events expected in this population include but are not limited to:

Acute kidney injury; acute renal failure; acute lung injury; adult respiratory distress syndrome; hypotension; metabolic acidosis; hypothermia; coagulation dysfunction; decreased platelet count; diffuse intravascular coagulation; thromboembolic event (pulmonary embolus; deep vein thrombosis; superficial thrombosis; cerebral infarction); fat embolism; myocardial infarction; decreased cardiac output; cardiac arrest; hepatic injury; sepsis and/or severe sepsis; septic shock; bacteremia; pleural effusion; abdominal compartment syndrome; repeated surgeries; amputation; cardiorespiratory insufficiency; pneumonia; electrolyte imbalance; ; pneumothorax; hemothorax; pain; fever; urinary tract infection; intraabdominal infection; respiratory insufficiency and/or failure; respiratory arrest; delirium; leukocytosis; fluid imbalance (overload, oliguria); cardiac enzyme abnormalities; nausea; vomiting; agitation; ileus (including post operative ileus); multi-system organ failure; anemia; hypotension; hypertension; fluid collections/abscess; bile leak; hemorrhagic shock; enterocutaneous fistula; gastrointestinal bleed; insomnia; elevated intra-cranial pressure; mesenteric ischemia; brain herniation; sympathetic storming

This clinical trial involves ICU patients with comorbidities, surgical, procedural and pharmacologic interventions and/or severe injuries. Death of a study participant due to his or her injuries or other known medical conditions is not unexpected.

**The relationship of the investigational product to the adverse event and severity of the event must be determined using the following classifications (Please note that the following AE grading criteria are a modification of Common Terminology Criteria for Adverse Events (CTCAE) from NIH):**

- **Relationship** to investigational product characterized as:
  - Unrelated – if there is not a reasonable possibility that the study drug caused the AE.
  - Unlikely – suggesting that only a remote connection exists between the study drug and the event.
  - Possible and Probable – suggesting that there exists a reasonable temporal sequence of the AE with the study drug.
- **Severity:** The following definitions should be used to determine the severity rating of all AEs:
  - Mild: Awareness of signs or symptoms, but these are easily tolerated and are transient and mildly irritating only. There is no loss of time from normal activities, and symptoms do not require medication or a medical evaluation.
  - Moderate: Discomfort enough to cause interference with usual activities or require therapeutic intervention, such as concomitant medication.
  - Severe: Incapacity with inability to work or do usual activities.

All AEs that are unexpected and possibly or probably related occurring during the study are to be followed up in accordance with GCP guidelines and will be followed until resolved; or if a chronic condition, until fully characterized.

All Suspected Unexpected Serious Adverse Drug Reactions (SUSARs) will be subject to expedited reporting. Additionally, post-study SUSARs that occur after the subject has completed a clinical study and are reported by the Investigator to the Sponsor (or an authorized representative) qualify for expedited reporting.

Suspected, unexpected serious adverse drug reactions (SUSARs) will be reported to the FDA, and IRBs within the required timeframes of seven calendar days for SUSARs which are fatal or life-threatening, and fifteen calendar days for all other SUSARs.

Study Drug Discontinuation Rules: Any thromboembolic event (MI, PE, Ischemic stroke, or symptomatic DVT) or obvious seizure activity while the study drug is being infused will warrant immediate cessation of the study drug administration and a medical safety and DSMB review.

**Written IND Safety Reports :** A written IND Safety Report (i.e., completed FDA Form 3500 A) will be reported to the responsible new drug review division of the FDA for any observed or volunteered adverse event that is determined to be a *serious and unexpected, suspected adverse reaction*. Each IND Safety Report will be prominently labeled, “IND Safety Report”, and a copy will be provided to all participating investigators (if applicable) and sub-investigators.

Written IND Safety Reports will be submitted to the FDA as soon as possible and, in no event, later than 15 calendar days following the Investigator’s receipt of the respective adverse event information and determination that it meets the respective criteria for reporting.

For each written IND Safety Report, the Investigator will identify all previously submitted IND Safety Reports that addressed a similar suspected adverse reaction experience; and will provide an analysis of the significance of newly reported, suspected adverse reaction in light of the previous, similar report(s), or any other relevant information.

Relevant follow-up information to an IND Safety Report will be submitted to the applicable review division of the FDA as soon as the information is available; and will be identified as such (i.e., “Follow-up IND Safety Report”).

If the results of the Investigator’s follow-up investigation show that an adverse event that was initially determined to not require a written IND Safety Report does, in fact, meet the requirements for reporting; the Investigator will submit a written IND Safety Report as soon as possible—but in no event later than 15 calendar days—after the determination was made.

**Telephoned IND Safety Reports – Fatal or Life-Threatening Suspected Adverse Reactions:** In addition to the subsequent submission of a written IND Safety Report (i.e., completed FDA Form 3500A), the Investigator will notify the responsible review division of the FDA by telephone or facsimile transmission of any *unexpected, fatal or life-threatening suspected adverse reaction.*

The telephone or facsimile transmission of applicable IND Safety Reports will be made as soon as possible, but in no event later than 7 calendar days after the Sponsor-Investigator’s receipt of the respective adverse event information and determination that it meets the respective criteria for reporting.

**Reporting Adverse Events to the Responsible IRB:** In accordance with applicable policies of the Washington University, St Louis Institutional Review Board (IRB), the Sponsor-Investigator will report, to the IRB, any observed or volunteered adverse event that is determined to be: 1) *associated with the investigational drug or study treatment(s)*; 2) *serious*; and, 3) *unexpected*. Adverse event reports will be submitted to the IRB in accordance with the respective IRB procedures.

Applicable adverse events will be reported to the IRB as soon as possible and, in no event, later than 10 calendar days following the sponsor-investigator’s receipt of the respective information. Adverse events which are 1) *associated with the investigational drug or study treatment(s)*; 2) *fatal or life-threatening*; and 3) *unexpected* will be reported to the IRB within 24 hours of the Sponsor-Investigator’s receipt of the respective information.

Follow-up information to a reported adverse event will be submitted to the IRB as soon as the relevant information is available. If the results of the Sponsor-Investigator’s follow-up investigation show that an adverse event that was initially determined to not require reporting to the IRB does, in fact, meet the requirements for reporting; the Sponsor-Investigator will report the adverse event to the IRB as soon as possible, but in no event later than 10 calendar days, after the determination was made.

**Human Protections**: This trial will be conducted under the Department of Health and Humans Services 21 CFR 50.24 Exception from Informed Consent for Emergency Research. As such, we will submit this protocol to our local Institutional Review Board for their review and approval in concert with a Community Consultation Proposal. The trial Investigators have submitted and received approval for their IND application to the FDA. All key personnel involved in the design or conduct of the research involving human subjects will receive the required education on the protection of human research participants prior to conducting this study. Patients’ risks of participating in research are kept to a minimum with measures to protect confidentiality and safety monitoring. Benefits outweigh risks as TXA has been shown to improve survival in 2 prior studies and all patients enrolled in this study will have additional assessments performed, including a duplex ultrasound of the lower extremities on hospital day 7 (+/- 2 days) or at discharge (- 2 days) (whichever comes first), which may identify deep vein thrombus formation in this high risk population that otherwise may not have been identified. This additional screening is a potential benefit in all three study groups since the identification of a deep vein thrombosis will inform the patient’s treating physicians of this condition and allow them to consider initiating treatment for this potentially life-threatening condition. In addition, we will disseminate the results of this clinical trial to the community and medical community as soon as possible. **The outcomes and safety data collected in this trial will be analyzed in a meta-analysis of the three US DoD funded clinical trials examining the efficacy and safety of TXA in severe trauma patients.**

**Special Considerations for Exception from Informed Consent for Emergency Research:**

The therapeutic window for the administration of TXA is two hours post injury (see following section for justification of this window). Since severely injured trauma patients will be unable to consent themselves, attempts to find a LAR will be made. If the LAR is available, standard LAR consent procedures will be used. A written consent form that complies with the policies of the Washington University IRB will be used (see Appendix A). In cases where a LAR is not available, any family member who may have accompanied the patient to the Hospital will be asked to provide permission on behalf of the patient. If nobody is physically available to provide permission, an attempt will be made to contact a family member based on any information available and permission over the phone will be gathered when applicable. We will presumptively enroll eligible patients using the exception of informed consent process if there isn’t anyone available to speak to on behalf of the patient. Attempts to locate the LAR will continue until an appropriate representative is identified and consent to continue the study can be obtained. All attempts and efforts will be documented in the subject’s study file to reflect the effort made to obtain proper informed consent as soon as was possible.

We are committed to obtaining consent from the LAR within the protocol time window and will document our efforts whenever the EFIC mechanism is used to enroll a patient. We will attempt to obtain consent in person, by telephone, email, fax, paper, and/ or any other communication possible on every patient prior to using EFIC. We will continue to seek consent from a legally-authorized representative after EFIC has been implemented. The LAR will be informed of the patient’s inclusion into the study and of the details and risks of the study. At the time, the LAR will be given the option of allowing the patient to continue in the study, or to cease the subject’s participation then or at any time throughout the course of the study. An informed consent form signed by the LAR will be obtained when possible.

If a subject who was enrolled into the study using EFIC expires before a LAR/family member has been reached, information about the subject’s enrollment in the study will be provided to the subject’s legally authorized representative (LAR) or family member at the earliest feasible opportunity via a letter mailed to the person and address that is made available in the subject’s medical record. Please note that it will not be uncommon in this study population for instances to occur where a subject’s LAR or family member is not able to be identified as is the case if the identity of the subject is **never determined**. In such cases, the attempt to identify the subject and his/her LAR/family member will be documented in the subject’s study record. All attempts to contact subjects’ LAR/family member will be documented in the subject’s study record.

In addition, if a subject who was enrolled into the study using EFIC is discharged from the hospital before his/her consent was able to be obtained (i.e. participant remained with diminished capacity at discharge) and/or his/her LAR were unable to be consented prior to discharge from the hospital, the subject and LAR will both be notified of subject’s participation in this study by letter. A letter will be mailed to the address provided in the medical record for the subject and LAR when available.

**Community Consultation:** The content of community consultation will inform the communities that informed consent will not be obtained for most (or all) research subjects. Specifically, the goal will be to; inform members of the surrounding communities about all relevant aspects of the study including its risks and expected benefits, hear the perspective of the communities on the proposed research and address questions and concerns, and to provide information about ways in which individuals wishing to be excluded may indicate this preference (i.e. “opt out” bracelets). The type and frequency of community consultation will; provide opportunities for broad community discussion, ensure that representatives from the community(ies) involved in the research participate in the consultation process, use the most appropriate ways to provide for effective community consultation, and be based on numerous factors, including the size of the community(ies), the languages spoken within those communities, the targeted research population and its heterogeneity. We will utilize focus groups, clubs/associations that may include members at high risk for trauma with blood loss, and use web-based outreach and surveys as mechanisms to provide community consultation.  Public disclosure methods will include newspaper, posters, internet based message boards, local magazines, etc. as sources. We will supply a mechanism for as many members of the community to “opt out” of participation in this trial (i.e. “opt out” bracelets). The information collected from community consultation will be compiled and reports completed and made available to trial site IRBs, DoD HRPO, and the FDA. Once WU IRB approval and DoD HRPO approval have been obtained, we will implement the Public Disclosure Plan.

**DSMB:** A DSMB will be established that will confidentially review interim/cumulative data for evidence of study-related adverse events and for quality, completeness, and timeliness. The DSMB established for the TAMPITI trial will monitor the implementation and safety of this study. DSMB membership of this advisory committee will consist of experts not involved in the planning or the conduct of TAMPITI and will be established by the Trial Investigators. Trial Investigators will convene the DSMB and provide an executive summary. DSMB membership will be comprised of an anesthesiologist, emergency department intensivists, a blood bank specialist, a pediatric hematologist/oncologist, biostatistician, and a physician with a background in ethics. The DSMB Chair will also serve as the Research Monitor. She will facilitate discussion, integrate differing points of view and move toward consensus on recommendations to be provided to the DoD, FDA and Trial Investigators. The Data Safety Monitoring Plan (DSMP) with the full DSMB charter is attached in Appendix B.

**Research Monitor**: For research determined to be greater than minimal risk, DoDI 3216.02 requires that the IRB approve, by name, an independent research monitor with expertise consonant with the nature of risk(s) identified within the research protocol. The research monitor may discuss the research protocol with the investigators, interview human subjects, and consult with others outside of the study about the research. We have appointed Jessica Zenga, MD to the role of the Research Monitor. Dr. Zenga is a physician within the Department of anesthesia. Her duties will include but may not be limited to the following:

1. Discussing the research protocol with the investigators;

2. Be the Data Safety Monitoring Board (DSMB) Chair and schedule meetings;

3. Speak with human subjects to ensure ongoing understanding of study related procedures and their continued interest in participating or withdrawing from study, serving as their advocate;

4. Shall have authority to stop a research protocol in progress, remove individual human subjects from a research protocol, and take whatever steps are necessary to protect the safety and well-being of human subjects until the IRB can assess the monitor's report;

5. Shall have the responsibility to promptly report their observations and findings to the IRB or other designated official and the HRPO.

**Subject Withdrawal:** Subjects will be enrolled into this study under an exception from informed consent

requirement for emergency research. Informed consent will be obtained, when possible, by the subject or

legally authorized representative (LAR). The subject and LAR will be advised by the research team of their

right to withdraw from study participation at any time without penalty or adverse affect on their routine medical

care. However, subjects will be encouraged to comply with all safety evaluations to ensure their safety. If a

subject elects to withdraw from the study they will be informed that all data obtained to this point will be

maintained for data analysis purposes but no further study procedures will take place.

If a subject is put into police custody after receiving study treatment, all study related procedures will be suspended immediately and the IRB will be notified about the newly-incarcerated prisoner-subject. The subject and/or his/her LAR will be notified of the end of participation when/if possible. All data obtained up until the point of withdrawal will be maintained for data analysis purposes.

**Sponsor-Investigator Discontinuation of the Clinical Research Study:** Both the Investigator and the DoD reserve the right to terminate the study at any time. Should this be necessary, the procedures will be arranged on an individual study basis after review and consultation with both parties. In terminating the study, the DoD and the Investigator will ensure that adequate consideration is given to the protection of the subjects’ interests and safety. The WUSL IRB will be notified immediately (verbally and in writing) of this decision as will all study participants.

**Record maintenance and retention:** The Investigator will maintain all case report forms and all source documents that support the data collected from each subject; and all trial documents, as specified by applicable regulatory requirement(s). The Investigator will take measures to prevent accidental or premature destruction of these documents.

Essential documents must be retained for at least 2 years after the last approval of a marketing application worldwide, or until at least 2 years have elapsed since the formal discontinuation of clinical development of the investigational product. These documents will be retained for a longer period if required by the applicable regulatory requirements. If the responsible Investigator retires, relocates, or for other reasons withdraws from the responsibility of keeping the study records, custody must be transferred to a person who will accept the responsibility.

The Investigator will retain the specified records and reports for up to 2 years after the marketing application is approved for the investigational drug; or, if a marketing application is not submitted or approved for the investigational drug, until 2 years after investigations under the IND have been discontinued and the FDA so notified.

**Data Handling**: A Case Report Form (CRF, see Appendix 1) will be completed for each subject enrolled into the clinical study. The Investigator will review, approve and sign/date each completed CRF; the Investigator’s signature serving as attestation of the Investigator’s responsibility for ensuring that all clinical and laboratory data entered on the CRF are complete, accurate and authentic.

*Source Data* are the clinical findings and observations, laboratory and test data, and other information contained in *Source Documents*. *Source Documents* are the original records (and certified copies of original records); including, but not limited to, hospital medical records, physician or office charts, physician or nursing notes, subject diaries or evaluation checklists, pharmacy dispensing records, recorded data from automated instruments, x-rays, etc. When applicable, information recorded on the CRF shall match the *Source Data* recorded on the *Source Documents*.

**Review of Research Records:** Representatives of the Under Secretary of Defense (Personnel & Readiness) are authorized to review research records as part of their responsibility to protect human research volunteers. In the event that a HIPAA authorization is required, include the above as parties to whom private health information may be disclosed.

**Appendix A**

**Acute Kidney Injury** is defined as an abrupt (within 48 hours) reduction in kidney function based on an elevation in serum creatinine level, a reduction in urine output, the need for renal replacement therapy (dialysis), or a combination of these factors. It is classified in three stages:

| **Stage** | **Change in serum creatinine level** | **Urine output** | **Other** |
| --- | --- | --- | --- |
| 1 | Increase ≥ 0.3 mg per dL (26.52 μmol per L) or ≥ 1.5- to twofold from baseline | < 0.5 mL per kg per hour for more than six hours | — |
| 2 | Increase > two- to threefold from baseline | < 0.5 mL per kg per hour for more than 12 hours | — |
| 3 | Increase > threefold from baseline or ≥ 4.0 mg per dL (353.60 μmol per L) with an acute rise of at least 0.5 mg per dL (44.20 μmol per L) | < 0.3 mL per kg per hour for 24 hours or anuria for 12 hours | Renal replacement therapy required |

note: *Each stage is defined by the change in serum creatinine level, the change in urine output, or the need for renal replacement therapy*.

*Mehta RL, Kellum JA, Shah SV, et al. Acute Kidney Injury Network: report of an initiative to improve outcomes in acute kidney injury*. Crit Care. *2007;11(2):R31*.

Stroke defined as by the development of a new neurological deficit identified on clinical examination correlating with CT scan or MRI findings of cerebral infarction in an anatomic location corresponding to the newly identified deficit.

Myocardial infarction will be defined by the Universal Definition of Myocardial Infarction. Either of the following criteria meets the diagnosis: Detection of rise and/or fall of cardiac biomarkers (preferably troponin) with at least one value above the 99^th^ percentile of the upper reference limit together with evidence of myocardial ischemia with at least one of the following, symptoms of ischemia, EKG changes indicative of new ischemia (new ST-T changes or new left bundle branch block (LBBB)), development of pathologic Q waves in the EKG, Imaging evidence of new loss of viable myocardium or new regional wall motion abnormality, and identification of an intracoronary thrombus by angiography or autopsy.

Pulmonary embolism will be defined by any of the following, computed tomography angiography of the thoracic vasculature demonstrating thrombus in the pulmonary arterial vessels, a ventilation/perfusion scan interpreted as having high probability for pulmonary embolism in the setting of new onset tachycardia or hypoxemia, death with autopsy confirmation of the presence of a pulmonary arterial thrombus or embolus.

Seizure is defined by the development of a new transient or persisting neurologic deficit identified on clinical examination and accompanied by electroencephalographic abnormality in an anatomic location corresponding to the newly identified deficit.

Multiple Organ Dysfunction Syndrome (MODS): progressive but potentially reversible physiologic dysfunction of 2 or more organ systems that arises after resuscitation from acute life threatening events.

| **Table 5:** Multiple Organ Dysfunction Score (MODS) | | | | | | |
| --- | --- | --- | --- | --- | --- | --- |
| Organ System Values | MODS Score | | | | | Normal Value Ranges |
|  | 0 | 1 | 2 | 3 | 4 |  |
| Haematologic: Platelet Count (x10^3^/mm^3^ or 10^9^/L) | > 120 | 81-120 | 51-80 | 21-50 | ≤ 20 | *> 120* |
| Hepatic: Serum Bilirubin (μmol/L) | ≤ 20 | 21-60 | 61-120 | 121-240 | > 240 | *≤ 20* |
| Renal: Serum Creatinine (μmol/L) | ≤ 100 | 101-200 | 201-350 | 351-500 | > 500 | *≤ 100* |
| Cardiovascular: PAR | ≤ 10 | 10.1-15 | 15.1-20 | 21-30 | > 30 | *≤ 10* |
| Glasgow Coma Score | 15 | 13-14 | 10-12 | 7-9 | ≤ 6 | *15* |
| Respiratory: PO_2_ / FiO_2_ | > 300 | 226-300 | 151-225 | 76-150 | ≤ 75 | *> 300* |

Sepsis: As defined by the Surviving Sepsis Campaign[[70](#_ENREF_70)], the definition of sepsis requires a systemic response to infection i.e confirmed infection plus 2 or more of the following criteria: Temperature > 38 °C or 36 °C, Heart rate > 90 beats/min, Respiratory rate > 20 breaths/min or PaCO 2 < 32 torr  (< 4.3 kPa), WBC > 12000 cells/mm 3, < 4000 cells/mm3, or > 10% immature(band) forms.

ARDS: As defined by the Berlin Criteria published in JAMA [June 20, 2012, Vol 307, No. 23](http://jama.jamanetwork.com/issue.aspx?journalid=67&issueid=24244).

**Transfusion guidelines:** If class IV shock or physician determination that patient will need massive transfusion (> 10U of RBCs in 6 hours) then the MTP will be initiated that follows all DCR principles as outlined by Spinella to include a 1:1:1 ratio of RBCs:Plasma:Platelets [[29](#_ENREF_29)]. Cryoprecipitate will be administered if fibrinogen values are < 150 mg/dl. No factor concentrates such as rFVIIa, prothrombin or fibrinogen concentrates will be administered to patients in this trial. If a patient does not meet MTP criteria the following indications will be used for the following blood products when the patient has active bleeding:

-2 units of RBCs for Hb < 9mg/dl

-2 units of plasma for INR> 1.5

-1 unit of apheresis platelets for a platelet count < 100,000

-10 units of cryoprecipitate for fibrinogen concentration < 150mg/dl

**DVT prophylaxis Protocol:** Participants will be placed on DVT prophylaxis according to the Barnes Jewish Hospital/Washington University ACCS Patient DVT Prophylaxis Guideline by their clinical care team (see Appendix C). The research team will capture information regarding the type of DVT prophylaxis ordered and details surrounding whether or not it was received daily, while the participant remains on study as part of the data collection and will urge the clinical care team to initiate DVT prophylaxis if it is identified that it has not been ordered. A Study Investigator will be notified if the research team identifies that a participant has not had his/her DVT prophylaxis initiated. Please note that it will not be considered a protocol violation or deviation if a patient does not receive DVT prophylaxis during study participation as such prophylaxis is managed by and is the responsibility of the clinical care team.

**Appendix B Data Safety Monitoring Plan:**

Patient safety is of paramount importance in this trial and there is an extensive set of procedures in place for monitoring adverse events. These procedures are as follows:

***Data Safety Monitoring Board (DSMB)***

A Data Safety Monitoring Board (DSMB) appointed by Drs. Bochicchio and Spinella, Clinical Principal Investigators of the study, will meet approximately quarterly (either by teleconference or face to face when possible) in closed sessions to review the progress of this study (e.g., enrollment, site performance) as well as data on the safety of each arm of the study. The meetings will consist of the DSMB chair and at least 3 members of the DSMB. If this minimum requirement cannot be met, the meeting will be rescheduled at the first available opportunity. Open sessions may occur at the request of the DSMB. Before the study begins, the DSMB (in consultation with the study statistician and the PIs) will decide the content of the reports to be presented to the board. Using guidelines established by the DSMB and the investigators before the study begins, the DSMB may recommend termination of the study if either of the treatment arms (2 gram TXA or 4 gram TXA) is found to be unsafe. Additionally, the DSMB may recommend modifications to the protocol if a correctable safety issue is identified. After each meeting, the DSMB Chair will prepare a letter to the study principal investigators, Drs. Grant Bochicchio and Phillip Spinella, which will describe the safety review that took place at the meeting and that indicates whether or not there are any safety concerns. This letter will be provided to the IRB according to their policies and procedures.

We have appointed an experienced clinician for the study (Dr. Jessica Zenga). Dr. Zenga will review all adverse events in the study as they occur. Dr. Zenga will report safety concerns that arise during the trial to the PIs and study team. Dr. Zenga will lead the DSMB meetings and will discuss any concerns about adverse events. Dr. Zenga has considerable experience with clinical trial safety and is serving on other DSMBs for research trials being conducted in our institution.

***Principal Investigators***

Drs. Bochicchio and Spinella will be informed of the DSMB’s quarterly assessment of study performance and safety.

***Data Safety Monitor Board Members:***

* Dr. Jessica Zenga, Data Safety Monitoring Chair/Research Monitor

Dr. Enyo Ablordeppey

Dr. George Despotis

Dr. Melanie Fields

Dr. James Fehr (serving as the ethicist for the trial)

Dr. Philip Miller (Biostatistician)

Dr. Kevin Ward

***Events to be Reviewed by DSMB:***

- All adverse events (AEs) that are unexpected and possibly or probably related to the study, will be reviewed quarterly by the DSMB. In addition, adverse events of special interest, regardless of study drug relationship will be reviewed by the DSMB. These events include seizures and all thromboembolic events.
- All SAE's will be reviewed by an Investigator within 24 hours of their occurrence.
- SAE’s determined (by an Investigator) to be expected and unrelated will be reported to the Research Monitor within 72 hours of being identified.
- All ***Unexpected ,Serious Adverse Events*** (SUA’S) thought to be related to study drug will be reported to the PI, Research Monitor and IRB immediately. Emergency medical treatment will be provided as necessary

***Stopping Rules:***

Subjects who experience any thromboembolic event (MI, PE, Ischemic stroke, or symptomatic DVT) or obvious seizure activity while the study drug is being infused will warrant immediate cessation of the study drug administration and a medical safety/DSMB review. The Research Monitor can temporarily place the trial on hold until the DSMB can confer to discuss any study concerns.

**Appendix C**

**Barnes Jewish Hospital/Washington University**

**ACCS Patient DVT Prophylaxis Guideline**

All ACCS Patients will be placed on DVT Prophylaxis unless the following indications exist:

Heparin Induced Thrombocytopenia

Plan to go to OR within next 12 hours

Plan to go to OR with Neurosurgery within next 24 hours

Liver or spleen injury diagnosis <24 hours old or unstable

Hemodynamically unstable secondary to bleeding

Trauma patients should be placed on Enoxaparin 30 mg SQ q12 hours unless:

• Their BMI<20, then consider dosing change

• Their BMI >40 and >100 kg then place on Enoxaparin 40 mg SQ q12 hours

• Cr Cl <30 ml/min then place on Heparin Sodium 5,000 unit SQ TID or 7,500 based on BMI>40

 _They have an epidural, then place on Heparin Sodium 5,000 unit SQ TID

ACES patients should be placed on Enoxaparin 40 mg SQ qPM:

Any patient that develops DVT may be changed to treatment dose of unfractionated heparin (gtt) or fractionated heparin (1mg/kg sq BID) at Attending discretion. If they cannot be anticoagulated fully – consider an IVC filter, depending on condition and location of clot.

Note: Individualized, clinical judgment supersedes all written guidelines.

| **Appendix D**  **TAMPITI Schedule of Events** | **Screening** | **Hour0** | **Baseline** | **10 Min** | **20 Min** | **40 Min** | **1**  **HR** | **1.5 HR** | **2 HR** | **3 HR** | **4 HR** | **6 HR** | **8HR** | **10HR** | **12HR** | **24HR** | **72 HR** | **Day 7^f^**  **^+/- 2 days^** | **During Hospital Admission** | **Hospital Discharge** |
| --- | --- | --- | --- | --- | --- | --- | --- | --- | --- | --- | --- | --- | --- | --- | --- | --- | --- | --- | --- | --- |
| **Informed Consent** | **X** |  |  |  |  |  |  |  |  |  |  |  |  |  |  |  |  |  |  |  |
| **Demography** | **X** |  |  |  |  |  |  |  |  |  |  |  |  |  |  |  |  |  |  |  |
| **Medical/Surgical History** | **X** |  |  |  |  |  |  |  |  |  |  |  |  |  |  |  |  |  |  |  |
| **Inclusion/Exclusion** | **X** |  |  |  |  |  |  |  |  |  |  |  |  |  |  |  |  |  |  |  |
| **Physical Exam** | **X** |  |  |  |  |  |  |  |  |  |  |  |  |  |  | **X** | **X** | **X** | **X** | **X** |
| **Vital Signs** | **X** |  | **X** |  |  |  |  |  |  |  |  |  |  |  |  | **X** | **X** | **X** | **X** | **X** |
| **Immune Parameters^a^** |  | **X** |  |  |  |  |  |  |  |  |  | **X** |  |  |  | **X** | **X** |  |  |  |
| **Pharmacodymanic/ Pharmacokinetic Odd Schedule^b^** |  | **X** |  | **X** |  | **X** |  | **X** |  | **X** |  | **X** |  | **X** |  | **X** |  |  |  |  |
| **Pharmacodymanic/ Pharmacokinetic Even Schedule^b^** |  | **X** |  |  | **X** |  | **X** |  | **X** |  | **X** | **X** | **X** |  | **X** |  |  |  |  |  |
| **Repository Samples^c^** |  | **X** |  |  |  |  | **X** |  |  |  |  | **X** |  |  |  | **X** | **X** |  |  |  |
| **Study Drug Administration** |  |  | **X^d^** |  |  |  |  |  |  |  |  |  |  |  |  |  |  |  |  |  |
| **Concomitant Medications** | **X** | **X** | **X** | **X** | **X** | **X** | **X** | **X** | **X** | **X** | **X** | **X** | **X** | **X** | **X** | **X** | **X** |  | **X** | **X** |
| **Adverse Events** |  |  | **X** | **X** | **X** | **X** | **X** | **X** | **X** | **X** | **X** | **X** | **X** | **X** | **X** | **X** | **X** |  | **X** | **X^g^** |
| **Data Capture^e^** | **X** | **X** | **X** | **X** | **X** | **X** | **X** | **X** | **X** | **X** | **X** | **X** | **X** | **X** | **X** | **X** | **X** |  | **X** | **X** |
| **Clinical Outcomes** |  |  |  |  |  |  |  |  |  |  |  |  |  |  |  | **X** |  |  | **X** | **X** |
| **DVT Screen by Duplex** |  |  |  |  |  |  |  |  |  |  |  |  |  |  |  |  |  | **X** |  |  |
| **NIRS ST02 measurements^h^** |  | **X** |  | **X** | **X** | **X** | **X** | **X** | **X** | **X** | **X** | **X** | **X** | **X** | **X** | **X** | **X** |  |  |  |

^a^ Immune Parameters measured at approximately 0, 6, 24 and 72 hours. Up to 6 mL per sample for a total of 24mL

^b^ Up to 4.5 mL per sample at time points 0, 1, 6, and 24, and up to 2.7 mL per sample for all other draws for a total of 27 mL drawn at approximate time points

^c^ Up to 13.5 mL per sample for a total of 67.5 mL drawn at approximate time points

^d^ Administered within 2 hours from known injury time. Any thromboembolic event (MI, PE, Ischemic stroke, or symptomatic DVT) or obvious seizure activity while the study drug is

being infused will warrant immediate cessation of the study drug administration and a medical safety and DSMB review

^e^ Daily assessments for up to 30 days after receiving study drug for any S/S of thromboembolic event (i.e. shortness of breath, chest pain, extremity swelling, fever of unknown etiology, etc. in

addition to collecting data on blood product administration, clinical lab findings, and other relevant clinical data.

^f^ Or hospital discharge (- 2days) whichever occurs first.

^g^ Adverse events will only be collected through day 30 or hospital discharge, whichever occurs first.

^h^ NIRS ST02 Measurements to coincide with odd and even blood draw schedule when possible. If NIRS is not performed it will not be considered a protocol deviation.

**Please note that the following 24 hour and 72 hour study procedures may be performed within +/- 4 hours: Physical exam, vital signs, concomitant medications, AEs, data capture and clinical outcomes

**References:**

1. Cap, A.P., et al., *Tranexamic acid for trauma patients: a critical review of the literature.* J Trauma, 2011. 71(1 Suppl): p. S9-14.

2. Brohi, K., et al., *Acute traumatic coagulopathy: initiated by hypoperfusion: modulated through the protein C pathway?* Ann Surg, 2007. 245(5): p. 812-8.

3. Hess, J.R., et al., *The coagulopathy of trauma: a review of mechanisms.* J Trauma, 2008. 65(4): p. 748-54.

4. Pusateri, A.E., et al., *Tranexamic Acid and Trauma: Current Status and Knowledge Gaps with Recommended Research Priorities.* Shock, 2012.

5. collaborators, C.-t., et al., *Effects of tranexamic acid on death, vascular occlusive events, and blood transfusion in trauma patients with significant haemorrhage (CRASH-2): a randomised, placebo-controlled trial.* Lancet, 2010. 376(9734): p. 23-32.

6. Morrison, J.J., et al., *Military Application of Tranexamic Acid in Trauma Emergency Resuscitation (MATTERs) Study.* Arch Surg, 2012. 147(2): p. 113-9.

7. Levy, J.H., *Antifibrinolytic therapy: new data and new concepts.* Lancet, 2010. 376(9734): p. 3-4.

8. Brohi, K., et al., *Acute coagulopathy of trauma: hypoperfusion induces systemic anticoagulation and hyperfibrinolysis.* J Trauma, 2008. 64(5): p. 1211-7; discussion 1217.

9. Raza, I., et al., *The incidence and magnitude of fibrinolytic activation in trauma patients.* J Thromb Haemost, 2012.

10. Winfield, R.D., et al., *Obese patients show a depressed cytokine profile following severe blunt injury.* Shock, 2012. 37(3): p. 253-6.

11. Winfield, R.D., et al., *Differences in outcome between obese and nonobese patients following severe blunt trauma are not consistent with an early inflammatory genomic response.* Crit Care Med, 2010. 38(1): p. 51-8.

12. Bokesch, P.M., et al., *A phase 2 prospective, randomized, double-blind trial comparing the effects of tranexamic acid with ecallantide on blood loss from high-risk cardiac surgery with cardiopulmonary bypass (CONSERV-2 Trial).* J Thorac Cardiovasc Surg, 2012. 143(5): p. 1022-9.

13. Levy, J.H., J.M. Bailey, and M. Salmenpera, *Pharmacokinetics of aprotinin in preoperative cardiac surgical patients.* Anesthesiology, 1994. 80(5): p. 1013-8.

14. Lu, W., et al., *Pharmacokinetics of recombinant transgenic antithrombin in volunteers.* Anesth Analg, 2000. 90(3): p. 531-4.

15. Abou-Diwan, C., et al., *Plasma and cerebral spinal fluid tranexamic acid quantitation in cardiopulmonary bypass patients.* J Chromatogr B Analyt Technol Biomed Life Sci, 2011. 879(7-8): p. 553-6.

16. Haywood-Watson, R.J., et al., *Modulation of syndecan-1 shedding after hemorrhagic shock and resuscitation.* PLoS One, 2011. 6(8): p. e23530.

17. Kozar, R.A., et al., *Plasma restoration of endothelial glycocalyx in a rodent model of hemorrhagic shock.* Anesth Analg, 2011. 112(6): p. 1289-95.

18. Pati, S., et al., *Protective effects of fresh frozen plasma on vascular endothelial permeability, coagulation, and resuscitation after hemorrhagic shock are time dependent and diminish between days 0 and 5 after thaw.* J Trauma, 2010. 69 Suppl 1: p. S55-63.

19. Letourneau, P.A., et al., *Fresh frozen plasma increases adhesion molecule expression on human pulmonary endothelial cells.* J Surg Res, 2010. 163(2): p. 317-22.

20. Maegele, M., P.C. Spinella, and H. Schochl, *The acute coagulopathy of trauma: mechanisms and tools for risk stratification.* Shock, 2012. 38(5): p. 450-8.

21. Cap, A.P., et al., *Timing and location of blood product transfusion and outcomes in massively transfused combat casualties.* J Trauma Acute Care Surg, 2012. 73(2 Suppl 1): p. S89-94.

22. Spinella, P.C., et al., *Fresh whole blood use for hemorrhagic shock: preserving benefit while avoiding complications.* Anesth Analg, 2012. 115(4): p. 751-8.

23. Spinella, P.C., et al., *Constant challenges and evolution of US military transfusion medicine and blood operations in combat.* Transfusion, 2012. 52(5): p. 1146-53.

24. Holcomb, J.B., et al., *Increased platelet:RBC ratios are associated with improved survival after massive transfusion.* J Trauma, 2011. 71(2 Suppl 3): p. S318-28.

25. Cap, A.P. and P.C. Spinella, *Severity of head injury is associated with increased risk of coagulopathy in combat casualties.* J Trauma, 2011. 71(1 Suppl): p. S78-81.

26. Peiniger, S., et al., *Balanced massive transfusion ratios in multiple injury patients with traumatic brain injury.* Crit Care, 2011. 15(1): p. R68.

27. Perkins, J.G., et al., *Comparison of platelet transfusion as fresh whole blood versus apheresis platelets for massively transfused combat trauma patients (CME).* Transfusion, 2011. 51(2): p. 242-52.

28. Spinella, P.C., et al., *Duration of red blood cell storage is associated with increased incidence of deep vein thrombosis and in hospital mortality in patients with traumatic injuries.* Crit Care, 2009. 13(5): p. R151.

29. Spinella, P.C. and J.B. Holcomb, *Resuscitation and transfusion principles for traumatic hemorrhagic shock.* Blood Rev, 2009. 23(6): p. 231-40.

30. Spinella, P.C., et al., *Warm fresh whole blood is independently associated with improved survival for patients with combat-related traumatic injuries.* J Trauma, 2009. 66(4 Suppl): p. S69-76.

31. Holcomb, J.B., et al., *Increased plasma and platelet to red blood cell ratios improves outcome in 466 massively transfused civilian trauma patients.* Ann Surg, 2008. 248(3): p. 447-58.

32. Spinella, P.C., *Warm fresh whole blood transfusion for severe hemorrhage: U.S. military and potential civilian applications.* Crit Care Med, 2008. 36(7 Suppl): p. S340-5.

33. Niles, S.E., et al., *Increased mortality associated with the early coagulopathy of trauma in combat casualties.* J Trauma, 2008. 64(6): p. 1459-63; discussion 1463-5.

34. Spinella, P.C., et al., *Effect of plasma and red blood cell transfusions on survival in patients with combat related traumatic injuries.* J Trauma, 2008. 64(2 Suppl): p. S69-77; discussion S77-8.

35. Spinella, P.C., et al., *The effect of recombinant activated factor VII on mortality in combat-related casualties with severe trauma and massive transfusion.* J Trauma, 2008. 64(2): p. 286-93; discussion 293-4.

36. Borgman, M.A., et al., *The ratio of blood products transfused affects mortality in patients receiving massive transfusions at a combat support hospital.* J Trauma, 2007. 63(4): p. 805-13.

37. Spinella, P.C., et al., *Risks associated with fresh whole blood and red blood cell transfusions in a combat support hospital.* Crit Care Med, 2007. 35(11): p. 2576-81.

38. Holcomb, J.B., et al., *Damage control resuscitation: directly addressing the early coagulopathy of trauma.* J Trauma, 2007. 62(2): p. 307-10.

39. Blackbourne, L.H., et al., *Military medical revolution: deployed hospital and en route care.* J Trauma Acute Care Surg, 2012. 73(6 Suppl 5): p. S378-87.

40. Montgomery, R.K., et al., *Enhanced shear-induced platelet aggregation due to low-temperature storage.* Transfusion, 2012.

41. Cap, A.P. and J.G. Perkins, *Lyophilized platelets: challenges and opportunities.* J Trauma, 2011. 70(5 Suppl): p. S59-60.

42. Pidcoke, H.F., et al., *Ten-year analysis of transfusion in Operation Iraqi Freedom and Operation Enduring Freedom: increased plasma and platelet use correlates with improved survival.* J Trauma Acute Care Surg, 2012. 73(6 Suppl 5): p. S445-52.

43. Bochicchio, G., et al., *Nosocomial Infections in Elderly Trauma Patients: Incidence and Microbiology.* Infections in Medicine, 2002. 19(11): p. 512-516.

44. Bochicchio, G., et al., *Use of a modified chitosan dressing in a hypothermic coagulopathic grade V liver injury model.* Am J Surg, 2009. 198(5): p. 617-22.

45. Bochicchio, G.V., et al., *Acute glucose elevation is highly predictive of infection and outcome in critically injured trauma patients.* Ann Surg, 2010. 252(4): p. 597-602.

46. Bochicchio, G.V., et al., *Early hyperglycemic control is important in critically injured trauma patients.* J Trauma, 2007. 63(6): p. 1353-8; discussion 1358-9.

47. Bochicchio, G.V., et al., *Impact of community-acquired infection on acquisition of nosocomial infection, length of stay, and mortality in adult blunt trauma patients.* Surg Infect (Larchmt), 2002. 3(1): p. 21-8.

48. Bochicchio, G.V., et al., *Impact of nosocomial infections in trauma: does age make a difference?* J Trauma, 2001. 50(4): p. 612-7; discussion 617-9.

49. Bochicchio, G.V., M. Joshi, and T. Scalea, *Community-acquired infections in the geriatric trauma population.* Shock, 2000. 14(3): p. 338-42.

50. Bochicchio, G.V., et al., *Evaluation of a new hemostatic agent in a porcine grade V liver injury model.* Am Surg, 2010. 76(3): p. 317-20.

51. Bochicchio, G.V., et al., *Persistent systemic inflammatory response syndrome is predictive of nosocomial infection in trauma.* J Trauma, 2002. 53(2): p. 245-50; discussion 250-1.

52. Bochicchio, G.V., et al., *Admission preoperative glucose is predictive of morbidity and mortality in trauma patients who require immediate operative intervention.* Am Surg, 2005. 71(2): p. 171-4.

53. Bochicchio, G.V., et al., *Pilot study of a web-based antibiotic decision management guide.* J Am Coll Surg, 2006. 202(3): p. 459-67.

54. Bochicchio, G.V., et al., *Persistent hyperglycemia is predictive of outcome in critically ill trauma patients.* J Trauma, 2005. 58(5): p. 921-4.

55. Brenner, M., et al., *Long-term impact of damage control laparotomy: a prospective study.* Arch Surg, 2011. 146(4): p. 395-9.

56. De Castro, G.P., et al., *New hemostatic dressing (FAST Dressing) reduces blood loss and improves survival in a grade V liver injury model in noncoagulopathic swine.* J Trauma, 2011. 70(6): p. 1408-12.

57. Guzzo, J.L., et al., *Prediction of outcomes in trauma: anatomic or physiologic parameters?* J Am Coll Surg, 2005. 201(6): p. 891-7.

58. Huskins, W.C., et al., *Intervention to reduce transmission of resistant bacteria in intensive care.* N Engl J Med, 2011. 364(15): p. 1407-18.

59. Kilbourne, M., et al., *Hemostatic efficacy of modified amylopectin powder in a lethal porcine model of extremity arterial injury.* Ann Emerg Med, 2009. 53(6): p. 804-10.

60. Kilbourne, M., et al., *Novel model of frontal impact closed head injury in the rat.* J Neurotrauma, 2009. 26(12): p. 2233-43.

61. Ruben, F.L., et al., *Clinical infections in the noninstitutionalized geriatric age group: methods utilized and incidence of infections. The Pittsburgh Good Health Study.* Am J Epidemiol, 1995. 141(2): p. 145-57.

62. Scalea, T.M., et al., *Tight glycemic control in critically injured trauma patients.* Ann Surg, 2007. 246(4): p. 605-10; discussion 610-2.

63. Scalea, T.M., et al., *Early aggressive use of fresh frozen plasma does not improve outcome in critically injured trauma patients.* Ann Surg, 2008. 248(4): p. 578-84.

64. Simard, J.M., et al., *Key role of sulfonylurea receptor 1 in progressive secondary hemorrhage after brain contusion.* J Neurotrauma, 2009. 26(12): p. 2257-67.

65. Stansbury, L.G., et al., *Controversy in trauma resuscitation: do ratios of plasma to red blood cells matter?* Transfus Med Rev, 2009. 23(4): p. 255-65.

66. Stein, D.M., et al., *Relationship of serum and cerebrospinal fluid biomarkers with intracranial hypertension and cerebral hypoperfusion after severe traumatic brain injury.* J Trauma, 2011. 70(5): p. 1096-103.

67. Sutton, E., et al., *Long term impact of damage control surgery: a preliminary prospective study.* J Trauma, 2006. 61(4): p. 831-4; discussion 835-6.

68. Xiao, Y., et al., *Video-based training increases sterile-technique compliance during central venous catheter insertion.* Crit Care Med, 2007. 35(5): p. 1302-6.

69. Pidcoke, H.F., et al., *Primary hemostatic capacity of whole blood: a comprehensive analysis of pathogen reduction and refrigeration effects over time.* Transfusion, 2013. 53(S1): p. 137S-149S.

70. Levy, M.M., et al., *Outcomes of the Surviving Sepsis Campaign in intensive care units in the USA and Europe: a prospective cohort study.* Lancet Infect Dis, 2012. 12(12): p. 919-24.

71. Casserly, B., et al., *Low-dose steroids in adult septic shock: results of the Surviving Sepsis Campaign.* Intensive Care Med, 2012. 38(12): p. 1946-54.

72. Dellinger, R., et al., *Surviving Sepsis Campaign: International Guidelines for Management of Severe Sepsis and Septic Shock: 2012.* Critical Care Medicine, 2013. 41(2): p. 580-637.

73. Karski, J.M., et al., *Prevention of bleeding after cardiopulmonary bypass with high-dose tranexamic acid. Double-blind, randomized clinical trial.* J Thorac Cardiovasc Surg, 1995. 110(3): p. 835-42.

74. Bernet, F., et al., *Reduction of blood loss and transfusion requirements after coronary artery bypass grafting: similar efficacy of tranexamic acid and aprotinin in aspirin-treated patients.* J Card Surg, 1999. 14(2): p. 92-7.

75. Wong, B.I., et al., *Aprotinin and tranexamic acid for high transfusion risk cardiac surgery.* Ann Thorac Surg, 2000. 69(3): p. 808-16.

76. Kalavrouziotis, D., et al., *High-dose tranexamic acid is an independent predictor of early seizure after cardiopulmonary bypass.* Ann Thorac Surg, 2012. 93(1): p. 148-54.

77. Bojko, B., et al., *Determination of tranexamic acid concentration by solid phase microextraction and liquid chromatography-tandem mass spectrometry: first step to in vivo analysis.* J Chromatogr B Analyt Technol Biomed Life Sci, 2011. 879(32): p. 3781-7.

78. Bellomo, R., et al., *Acute renal failure - definition, outcome measures, animal models, fluid therapy and information technology needs: the Second International Consensus Conference of the Acute Dialysis Quality Initiative (ADQI) Group.* Crit Care, 2004. 8(4): p. R204-12.

79. Weingart S, Meyers CM. (March 1, 2008.) *Thoughts on the resuscitation of the critically ill trauma*

*patient*. EMCrit Blog. Retrieved Feb. 10, 2014, from [www.emcrit.org/podcasts/trauma-resus-part-i/](http://www.emcrit.org/podcasts/trauma-resus-part-i/).
80. Ho AM, Karmakar MK, Contardi LH, et al. *Excessive use of normal saline in managing traumatized*

*patients in shock: A preventable contributor to acidosis*. J Trauma. 2001;51(1):173–177.
81. Brohi K, Singh J, Heron M, et al. *Acute traumatic coagulopathy*. J Trauma. 2003;54(6):1127–1130.

82. McLeod JB, Lynn M, McKenney MG, et al. *Early coagulopathy predicts mortality in trauma*. J Trauma.

2003;55(1):39–44.
83. Maegele M, Lefering R, Yucel N, et al. *Early coagulopathy in multiple injury: An analysis from the*

*German Trauma Registry on 8,724 patients* . Injury. 2007;38(3):298–304.
84. Lewis AM. *Trauma triad of death emergency*. Nursing. 2000;30(3):62–64.

85. Kalavrouziotis D, Voisine P, Mohammadi S, et al. *High-dose tranexamic acid is an*

*Independent predictor of early seizure after cardiopulmonary bypass*nn Thorac Surg 2012;93:148

–55.

86. [Kirchhoff C](http://www.ncbi.nlm.nih.gov/m/pubmed/?term=Kirchhoff%20C%5BAuthor%5D)^1^, [Biberthaler P](http://www.ncbi.nlm.nih.gov/m/pubmed/?term=Biberthaler%20P%5BAuthor%5D), [Mutschler WE](http://www.ncbi.nlm.nih.gov/m/pubmed/?term=Mutschler%20WE%5BAuthor%5D), et al.Crit Care. 2009;13(3):R88. doi: 10.1186/cc7914. Epub

2009 Jun 11.

**Appendix B**

ICMJE forms
